# Abundant ammonia and nitrogen-rich soluble organic matter in samples from asteroid (101955) Bennu

**DOI:** 10.1038/s41550-024-02472-9

**Published:** 2025-01-29

**Authors:** Daniel P. Glavin, Jason P. Dworkin, Conel M. O’D. Alexander, José C. Aponte, Allison A. Baczynski, Jessica J. Barnes, Hans A. Bechtel, Eve L. Berger, Aaron S. Burton, Paola Caselli, Angela H. Chung, Simon J. Clemett, George D. Cody, Gerardo Dominguez, Jamie E. Elsila, Kendra K. Farnsworth, Dionysis I. Foustoukos, Katherine H. Freeman, Yoshihiro Furukawa, Zack Gainsforth, Heather V. Graham, Tommaso Grassi, Barbara Michela Giuliano, Victoria E. Hamilton, Pierre Haenecour, Philipp R. Heck, Amy E. Hofmann, Christopher H. House, Yongsong Huang, Hannah H. Kaplan, Lindsay P. Keller, Bumsoo Kim, Toshiki Koga, Michael Liss, Hannah L. McLain, Matthew A. Marcus, Mila Matney, Timothy J. McCoy, Ophélie M. McIntosh, Angel Mojarro, Hiroshi Naraoka, Ann N. Nguyen, Michel Nuevo, Joseph A. Nuth, Yasuhiro Oba, Eric T. Parker, Tanya S. Peretyazhko, Scott A. Sandford, Ewerton Santos, Philippe Schmitt-Kopplin, Frederic Seguin, Danielle N. Simkus, Anique Shahid, Yoshinori Takano, Kathie L. Thomas-Keprta, Havishk Tripathi, Gabriella Weiss, Yuke Zheng, Nicole G. Lunning, Kevin Righter, Harold C. Connolly, Dante S. Lauretta

**Affiliations:** 1 Solar System Exploration Division, NASA Goddard Space Flight Center (GSFC), Greenbelt, MD USA; 2 Earth and Planets Laboratory, Carnegie Institution for Science, Washington, DC USA; 3 Department of Geosciences, Pennsylvania State University, University Park, PA USA; 4 Lunar and Planetary Laboratory, University of Arizona, Tucson, AZ USA; 5 Lawrence Berkeley National Laboratory, Berkeley, CA USA; 6 Astromaterials Research and Exploration Science Division, NASA Johnson Space Center, Houston, TX USA; 7 NASA Headquarters, Washington, DC USA; 8 Center for Astrochemical Studies, Max Planck Institute for Extraterrestrial Physics, Garching, Germany; 9 Department of Chemistry, Catholic University of America, Washington, DC USA; 10 Center for Research and Exploration in Space Science and Technology, NASA GSFC, Greenbelt, MD USA; 11 ERC, Inc., JETS/Jacobs, Houston, TX USA; 12 California State University San Marcos, San Marcos, CA USA; 13 Center for Space Sciences and Technology, University of Maryland Baltimore County, Baltimore, MD USA; 14 Department of Earth Science, Tohoku University, Sendai, Japan; 15 Space Science Laboratory, University of California, Berkeley, CA USA; 16 Southwest Research Institute, Boulder, CO USA; 17 Robert A. Pritzker Center for Meteoritics and Polar Studies, Negaunee Integrative Research Center, Field Museum of Natural History, Chicago, IL USA; 18 Department of the Geophysical Sciences, University of Chicago, Chicago, IL USA; 19 Jet Propulsion Laboratory, California Institute of Technology, Pasadena, CA USA; 20 Department of Earth, Environmental, and Planetary Science, Brown University, Providence, RI USA; 21 Amentum, JSC Engineering and Technical Support (JETSII) Contract, NASA Johnson Space Center, Houston, TX USA; 22 Biogeochemistry Research Center, Japan Agency for Marine-Earth Science and Technology (JAMSTEC), Natsushima, Yokosuka, Japan; 23 Technical University Munich, Freising, Germany; 24 Research Unit Analytical Biogeochemistry, Helmholtz Munich, Neuherberg, Germany; 25 National Museum of Natural History, Smithsonian Institution, Washington, DC USA; 26 Oak Ridge Associated Universities, Oak Ridge, TN USA; 27 Department of Earth and Planetary Sciences, Kyushu University, Fukuoka, Japan; 28 NASA Ames Research Center, Moffett Field, CA USA; 29 Institute of Low Temperature Science, Hokkaido University, N19W8 Kita-ku, Sapporo, Japan; 30 Department of Physics, Technische Universität München, Muenchen, Germany; 31 Institute for Advanced Biosciences, Keio University, Kakuganji, Tsuruoka, Yamagata, Japan; 32 Barrios, JETS/Jacobs, Houston, TX USA; 33 Southeastern Universities Research Association, Washington, DC USA; 34 Department of Earth and Environmental Sciences, University of Rochester, Rochester, NY USA; 35 Department of Geology, School of Earth and Environment, Rowan University, Glassboro, NJ USA; 36 Department of Earth and Planetary Sciences, American Museum of Natural History, New York, NY USA

**Keywords:** Asteroids, comets and Kuiper belt, Astrobiology

## Abstract

Organic matter in meteorites reveals clues about early Solar System chemistry and the origin of molecules important to life, but terrestrial exposure complicates interpretation. Samples returned from the B-type asteroid Bennu by the Origins, Spectral Interpretation, Resource Identification, and Security–Regolith Explorer mission enabled us to study pristine carbonaceous astromaterial without uncontrolled exposure to Earth’s biosphere. Here we show that Bennu samples are volatile rich, with more carbon, nitrogen and ammonia than samples from asteroid Ryugu and most meteorites. Nitrogen-15 isotopic enrichments indicate that ammonia and other N-containing soluble molecules formed in a cold molecular cloud or the outer protoplanetary disk. We detected amino acids (including 14 of the 20 used in terrestrial biology), amines, formaldehyde, carboxylic acids, polycyclic aromatic hydrocarbons and N-heterocycles (including all five nucleobases found in DNA and RNA), along with ~10,000 N-bearing chemical species. All chiral non-protein amino acids were racemic or nearly so, implying that terrestrial life’s left-handed chirality may not be due to bias in prebiotic molecules delivered by impacts. The relative abundances of amino acids and other soluble organics suggest formation and alteration by low-temperature reactions, possibly in NH_3_-rich fluids. Bennu’s parent asteroid developed in or accreted ices from a reservoir in the outer Solar System where ammonia ice was stable.

## Abstract (web summary)

Rocks and dust from the asteroid Bennu contain some of the molecular building blocks of life on Earth, such as amino acids and nucleobases. They also carry ammonia that formed billions of years ago in cold, distant regions of our Solar System.

## Main

Primitive asteroids—those whose bulk chemistry was established in the protoplanetary disk—record processes that occurred during the formation and evolution of the early Solar System. The transport and delivery of organic compounds from these bodies could have been a source of molecules available for the emergence of life on Earth and potentially elsewhere.

Carbonaceous chondrite (CC) meteorites are samples of primitive carbon-rich bodies. In particular, the CI, CM, CR, CY and C2_ung_ (Ivuna-like, Mighei-like, Renazzo-like, Yamato-like and ungrouped-type-2, respectively) CCs have experienced moderate to extensive aqueous alteration (reactions with liquid water) in their parent bodies and typically contain ~1–3 wt% total carbon, with rare instances up to ~5 wt% (ref. ^1^). Organic carbon is primarily found in structurally complex insoluble organic matter and a diverse mixture of soluble organic matter (SOM) that contains prebiotic organic molecules (ref. ^2^ and the references therein). However, it is often unclear which Solar System objects are the parent bodies of CCs^3^. Furthermore, they experience alteration upon exposure to the terrestrial environment^4^, making interpretation challenging. The Origins, Spectral Interpretation, Resource Identification, and Security–Regolith Explorer (OSIRIS-REx) mission collected pristine material from the well-characterized surface of primitive B-type asteroid (101955) Bennu and delivered it to Earth under controlled conditions to minimize contamination and protect against atmospheric entry effects^5^.

Spacecraft observations made in proximity to Bennu corroborated preflight predictions^6,7^ of a carbon-rich composition, including strong aliphatic and aromatic organic carbon features at 3.4 μm, consistent with carbon abundances up to ~2.5 wt% and a low-temperature (<100 °C) aqueous alteration history^8,9^. A much weaker spectral feature observed at 3.1 μm could be consistent with some NH-bearing phases^9^, such as ammonium salts or N-rich organic matter. The remote sensing data also confirmed that Bennu is a rubble pile^7^, consisting of reaccumulated fragments of a larger, catastrophically disrupted asteroid (hereafter, parent body).

The spacecraft collected regolith (unconsolidated granular material) from as deep as ~0.5 m in Hokioi crater^10^, which is thought to be a recent impact site on Bennu based on its redder than average spectral slope^11^, and it delivered a total sample mass of 121.6 g to Earth^5^. Early laboratory analyses found C contents of 4.5–4.7 wt% and N contents of 0.23–0.25 wt% (ref. ^5^). The regolith’s hydrated mineralogy^5^ suggests that Bennu’s parent body accreted ices, which condensed from the outer protoplanetary disk.

Given Bennu’s compositional resemblance to aqueously altered CIs and CMs^5–9^, we hypothesized^12^ that the samples would contain a similar suite of organic compounds—including molecules found in biology, such as protein amino acids with left-handed enantiomeric excesses^13^, carboxylic acids, purines, pyrimidines and their precursors—and similar abundances and distributions of SOM. To test these hypotheses and explore the implications for Bennu’s parent body, we analysed organic matter in four aggregate (unsorted bulk) Bennu samples: two samples consisting of mostly fine particles (<100 μm) retrieved from spillover onto the avionics deck of the sample return canister^5,12^ and two samples containing a mixture of fine and intermediate (100–500 μm) particles removed from inside the Touch-and-Go Sample Acquisition Mechanism (TAGSAM)^14^ (Methods).

## Results

**Fig. 1:**
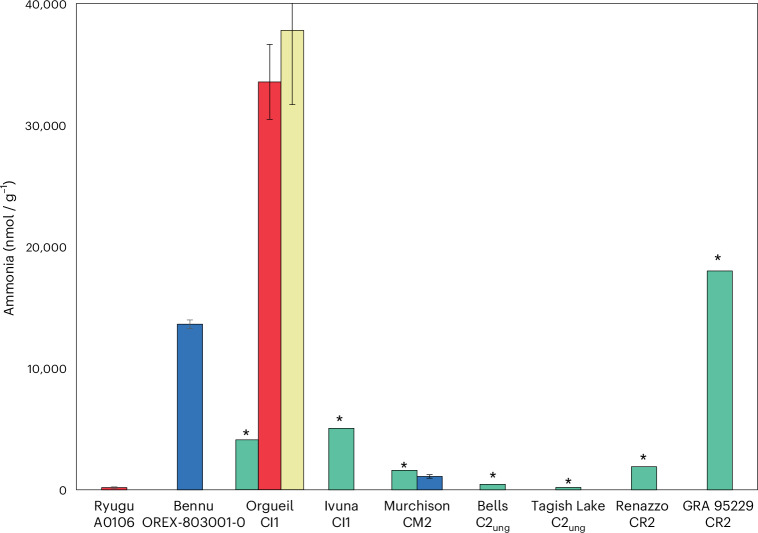
Concentrations of free ammonia measured in the extracts of Bennu (sample OREX-803001-0), Ryugu (sample A0106) and selected CCs. Data from this study (Extended Data Table 2) for the hot-water extracts of Bennu and Murchison (blue bars); data from hot-water extracts of Ryugu and Orgueil^21^ (red bars); data from cold-water leachates of Orgueil^22^ (yellow bar); and data from water and dichloromethane:methanol (9:1 v/v) extracts of Orgueil, Ivuna, Murchison, Bells, Tagish Lake (lithology not specified), Renazzo and GRA 95229 (ref. ^16^) (green bars). Data is presented as mean values ± the standard error of the mean. Estimated concentration of free ammonia is indicated by an asterisk taken from the data shown in Fig. 1a in ref. ^16^ and did not include errors. The large difference in ammonia concentrations measured in the Orgueil meteorite extracts could be due to differences in the extraction and analytical methods used^16,21,22^ and/or sample heterogeneity. Source Data

We conducted elemental analyser–isotope ratio mass spectrometry (EA-IRMS) measurements of Bennu aggregates, including a hot-water extract and solid residue (Methods), and found comparable total abundances of C (4.5–4.7 wt%) and N (0.23–0.25 wt%) as the early analyses^5^ (Extended Data Table 1). The hot-water extract was enriched in ^15^N (+180 ± 47‰) (Extended Data Table 1 and Supplementary Table 4) and had a high concentration of ammonia ~13.6 μmol g^−^^1^ (Fig. 1 and Extended Data Table 2). The ammonia concentration corresponded to ~40% of the estimated total N in the Bennu hot-water extract before dry-down (Extended Data Table 1 and Supplementary Table 4). The large ^15^N indicates that the ammonia was not derived from comparatively ^15^N-depleted spacecraft hydrazine propellant (δ^15^N = +4.7‰; Supplementary Information).

**Fig. 2:**
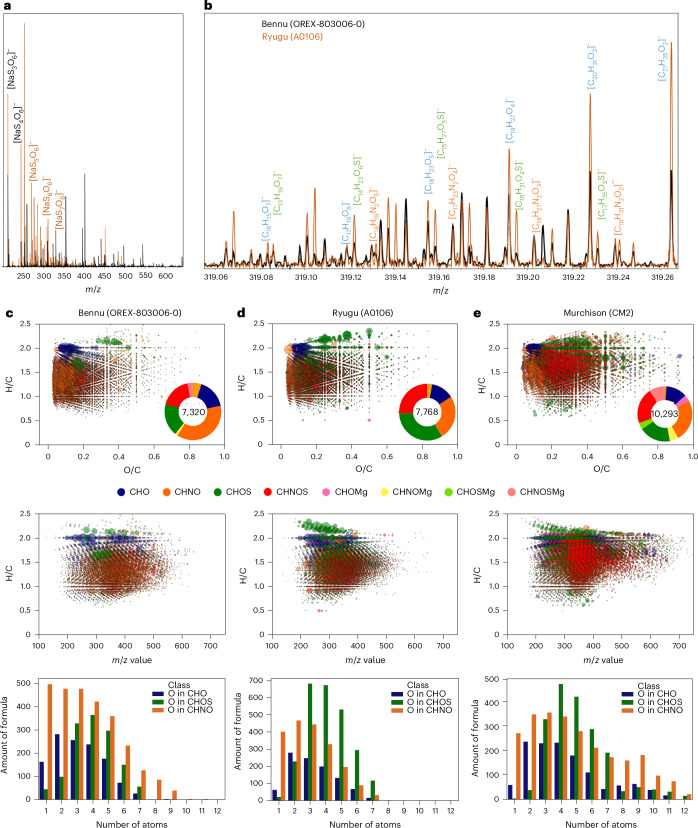
FTICR-MS data in electrospray ionization mode of the methanol extracts from Bennu (OREX-803006-0), compared with Ryugu (A0106) and Murchison. **a**, Mass spectra of Bennu (black) and Ryugu (orange) samples showing the relative abundance of polythionates with three to seven S atoms. **b**, Detail around *m*/*z* = 319 with major annotated elementary compositions (complete annotation can be found in Supplementary Fig. 3). **c**–**e**, Data visualization of the chemical compositions and number of molecules in Bennu (**c**) compared with Ryugu (**d**) and Murchison (**e**). Top, the Van Krevelen diagrams of H/C versus O/C atomic ratios of the compositional data as obtained from exact mass analysis. Coloured annuli enclose the total number of molecules assigned by mass, with colours indicating the relative abundances of the chemical families. Individual data points use the same colours to specify each family, and the size of each bubble reflects the intensity of the signal from the mass spectrum. Middle, the H/C atomic ratios as a function of *m*/*z* from 100 to 700. Bottom, the number of molecular formulae as a function of number of oxygen atoms in the CHO, CHOS and CHNO chemical families. Source Data

**Extended Data Fig. 1:**
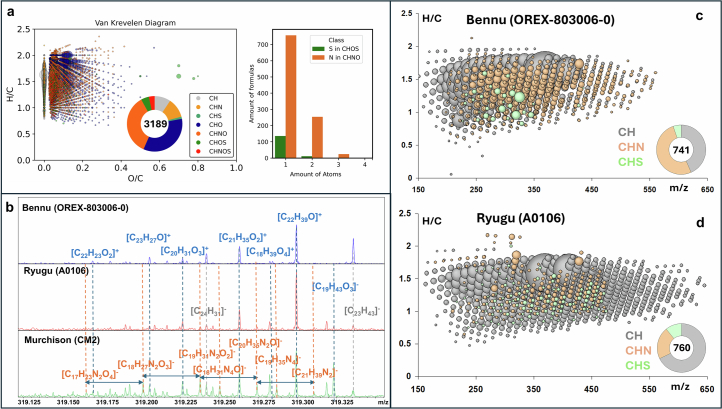
APPI(+) ionization FTICR-MS analysis of Bennu (OREX-803006-0) compared to Murchison and Ryugu (A0106). **a**, The low oxygenated molecules observed in the Bennu methanol extract as illustrated in the Van Krevelen diagram. **b**, Details around nominal mass *m/z* = 319 with the annotation of some *m/z* signals. The H/C versus *m/z* diagrams of **c**, Bennu and **d**, Ryugu, showing the lower molecular mass and higher relative abundance of CHN in the Bennu sample also in oxygen poor molecules. Colored annuli enclose the total number of molecules assigned by mass, with colors indicating the relative ratios of the chemical families. Source data

We performed untargeted analyses of methanol extracts of Bennu aggregate using Fourier-transform ion cyclotron resonance–mass spectrometry (FTICR-MS; Methods). The mass spectra of the extracts contained tens of thousands of compounds with mass-to-charge ratios (*m*/*z*) between 100 and 700 that correspond to ~16,000 molecular formulae consisting of C, H, N, O, S and Mg (Fig. 2). We identified a continuum of molecular sizes, with a range of carbon oxidation states, from non-polar or slightly polar—including polycyclic aromatic hydrocarbons, alkylated polycyclic aromatic hydrocarbons and a homologous series of unsaturated substituted aliphatic molecules—to more polar small molecules containing only CHO, CHNO, CHOS or CHNOS (Fig. 2, Extended Data Fig. 1 and Supplementary Fig. 15). The SOM is characterized by its nitrogen-rich chemistry, with up to seven nitrogen atoms per molecule detected by means of photoionization (APPI^+^) and electrospray ionization (ESI^−/+^), respectively (Fig. 2 and Extended Data Fig. 1).

**Fig. 3:**
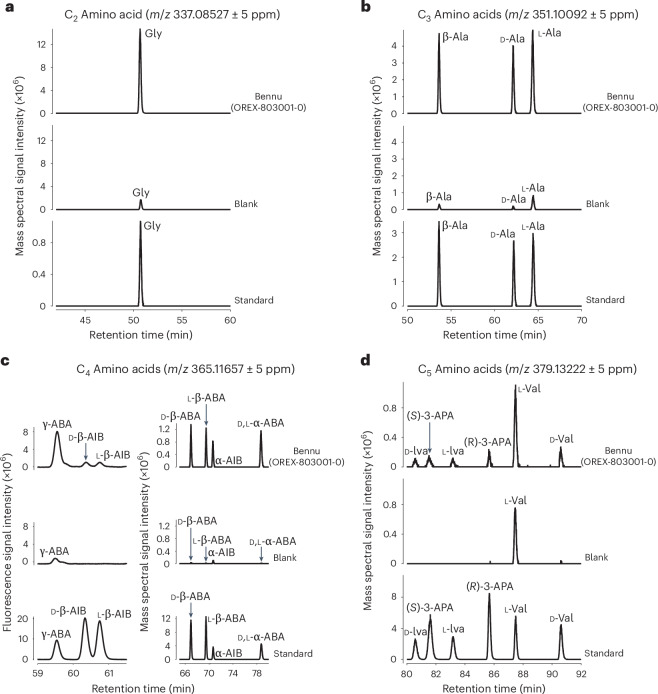
Amino acids identified by liquid chromatography mass spectrometry in the acid-hydrolysed, hot-water extract of Bennu (OREX-800031-0). **a**–**d**, Partial chromatograms obtained by LC-FD/HRMS after analysis of the standard and the 6 M HCl-hydrolysed water extracts of the FS-120 (blank) and Bennu (OREX-803001-0). **a**, Single-ion mass chromatograms at *m*/*z* 337.08527 corresponding to the C_2_ amino acid glycine (Gly). **b**, Single-ion mass chromatograms at *m*/*z* 351.10092 corresponding to the C_3_ amino acids β-alanine (β-Ala), d-alanine (d-Ala) and l-alanine (l-Ala). **c**, Right, single-ion mass chromatograms at *m*/*z* 365.11657 corresponding to the C_4_ amino acids d-β-amino-*n*-butyric acid (d-β-ABA), l-β-amino-*n*-butyric acid (l-β-ABA), α-aminoisobutyric acid (α-AIB) and d,l-α-amino-*n*-butyric acid (d,l-α-ABA). Left, the UV fluorescence separation and detections of the C_4_ amino acids γ-amino-*n*-butyric acid (γ-ABA) and d- and l-β-aminoisobutryic acids (d- and l-β-AIB). These C_4_ amino acids were also detected in the single-ion chromatogram at *m*/*z* 365.11657; however, a large *o*-phthaldialdehyde/N-acetyl-l-cysteine (OPA/NAC) derivative peak eluted at a similar time as these amino acids, suppressing amino acid peak intensities. **d**, Single-ion mass chromatograms at *m*/*z* 379.13222 corresponding to the C_5_ amino acids d-isovaline (d-Iva), l-isovaline (l-Iva), (*S*)-3-aminopentanoic acid (*S*-3-APA), (*R*)-3-aminopentanoic acid (*R*-3-APA), l-valine (l-Val) and d-valine (d-Val). The amino acids detected in the blank are likely to be derived from the solvents and derivatization reagents used for sample processing and analysis. Source Data

**Extended Data Fig. 2:**
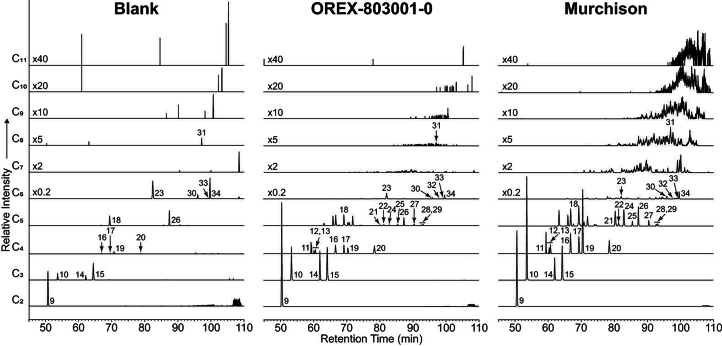
LC-HRMS chromatograms showing the elution of C_2_ to C_11_ amino acids in the acid-hydrolyzed, hot-water extracts of the procedural blank, Bennu (OREX-803001-0), and Murchison. The 45–110 min regions of the LC-HRMS single-ion mass chromatograms corresponding to the *o*-phthaldialdehyde/*N*-acetyl-l-cysteine (OPA/NAC) derivatives of C_2_ to C_11_ aliphatic primary amino acids in positive ion mode via heated electrospray ionization and a 5 ppm mass accuracy with corresponding *m/z* values as follows: **C2**: *m/z* = 337.08527; **C3**: *m/z* = 351.10092; **C4**: *m/z* = 365.11657; **C5**: *m/z* = 379.13222; **C6**: *m/z* = 393.14787; **C7**: *m/z* = 407.16352; **C8**: *m/z* = 421.17917; **C9**: *m/z* = 435.19482; **C10**: *m/z* = 449.21047; and **C11**: *m/z* = 463.22612. Similar single-ion chromatograms were obtained for the non-hydrolyzed water extracts. Peaks were identified by comparisons of their retention times and exact monoisotopic masses with those in the amino acid standard analyzed on the same day, and are designated by peak number as follows: (9) glycine, (10) β-alanine, (11) γ-amino-*n*-butyric acid, (12) d-β-aminoisobutyric acid, (13) l-β-aminoisobutyric acid, (14) d-alanine, (15) l-alanine, (16) d-β-amino-*n*-butyric acid, (17) l-β-amino-*n*-butyric acid, (18) δ-aminovaleric acid, (19) α-aminoisobutyric acid, (20) d,l-α-amino-*n*-butyric acid, (21) d-isovaline, (22) (S)-3-aminopentanoic acid, (23) ε-amino-*n*-caproic acid, (24) l-isovaline, (25) (R)-3-aminopentanoic acid, (26) l-valine, (27) d-valine, (28) d-norvaline, (29) l-norvaline, (30) l-isoleucine, (31) 8-aminooctanoic acid, (32) d-isoleucine, (33) d-leucine, and (34) l-leucine.­­ Note: the C_7_–C_11_ traces for the procedural blank and Bennu analyses feature spurious signal spikes that are due to instrumental background noise and do not represent analyte peaks. Source data

We surveyed for amino acids using pyrolysis gas chromatography–triple quadrupole–mass spectrometry (pyGC-QqQ-MS; Methods and Supplementary Fig. 14). We then determined the abundances and enantiomeric ratios of amino acids in a hot-water extract by means of liquid chromatography with ultraviolet (UV) fluorescence detection and mass spectrometry (LC-FD/MS; Fig. 3, Methods, Extended Data Fig. 2 and Supplementary Figs. 8 and 9).

A total of 33 amino acids were identified in the Bennu aggregates along with an uncounted suite of C_6_ and C_7_ aliphatic amino acids that were also detected but were not identified by name with standards (Fig. 3, Extended Data Tables 2 and 3 and Extended Data Fig. 2). These included 14 of the 20 standard protein amino acids used in terrestrial biology (Supplementary Table 12), all previously reported in meteorites^13^. Glycine was the most abundant amino acid (44 nmol g^−1^), with the majority in a free form, that is, without acid hydrolysis (Extended Data Table 3). Methionine, tyrosine and asparagine were tentatively detected at trace levels above background near the 0.1 nmol g^−1^ detection limit (Supplementary Table 12).

In addition, 19 non-protein amino acids were identified (Extended Data Table 3 and Supplementary Figs. 6, 8 and 9). All possible isomers of the C_3_ to C_5_ primary aliphatic amino acids were identified in the hot-water extract, as well as leucine, isoleucine and ε-amino-*n*-caproic acid at trace levels (Fig. 3, Extended Data Table 3 and Supplementary Figs. 8 and 9).

All chiral non-protein amino acids that could be enantiomerically resolved, including isovaline, norvaline, β-amino-*n*-butyric acid, β-aminoisobutyric acid and 3-aminopentanoic acid, were present as racemic or near racemic mixtures (equal abundances of d- and l-enantiomers) within analytical uncertainties (Extended Data Table 4). The detection of racemic alanine and aspartic acid within error indicates that the sample was pristine, with negligible biological l-protein amino acid contamination. An l-valine excess of ~34% was measured in the same hot-water extract after acid hydrolysis (Extended Data Table 4); however, we also observed elevated levels of l-valine in the procedural blank (Fig. 3), so laboratory contamination is a possible explanation. Isotopic measurements of valine will be needed to constrain the origin of the measured l-excess in the Bennu extract.

**Fig. 4:**
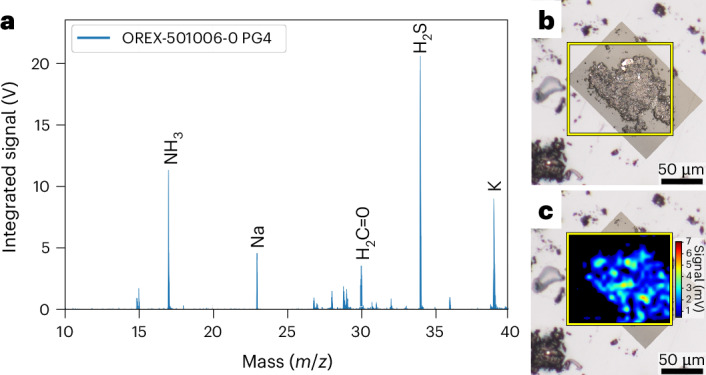
Ammonia and formaldehyde in Bennu (OREX-501006-0) identified by μ-L^2^MS. **a**, Summed mass spectrum acquired from several ~100 μm grains mounted on a KBr window with mass peaks for ammonia (NH_3_), sodium (Na), formaldehyde (H_2_C=O), hydrogen sulfide (H_2_S) and potassium (K) indicated. Spectrum acquired by μ-L^2^MS using a vacuum UV photoionization at 118 nm. **b**, Optical mosaic of particle with yellow box demarking region mapped by μ-L^2^MS. **c**, Spatial map of ammonia distribution overlaid over optical image. The μ-L^2^MS laser beam spot size was 5 μm. Source Data

Ammonia and formaldehyde are potential precursors for the synthesis of amino acids and other soluble organic molecules and were key targets for this investigation. Ammonia was independently identified, along with formaldehyde, in an avionics deck sample using micro two-step laser mass spectrometry (μ-L^2^MS) (Fig. 4 and Methods). Ammonia was also heterogeneously distributed in these particles at the ~5 μm scale (Fig. 4). Most of the ammonia in the Bennu aggregate samples was likely to have been originally retained as salts or bound to clay minerals or organic matter^15,16^ because highly volatile free ammonia is prone to loss. Volatile methylamine (914 nmol g^−1^) and ethylamine (121 nmol g^−1^), which are derivatives of ammonia, dominated the 16 aliphatic primary amines identified in the hot-water extract (Extended Data Table 2 and Supplementary Fig. 6) and were also likely to be present as salts.

Nine C_1_–C_7_ monocarboxylic acids and two dicarboxylic acids were identified in the hot-water extract by GC-QqQ-MS (Extended Data Table 5 and Supplementary Fig. 10). Formic (4,106 nmol g^−1^) and acetic (1,436 nmol g^−1^) acids were the two most abundant carboxylic acids detected.

At least 23 different N-heterocycles, including all five canonical biological nucleobases (adenine, guanine, cytosine, thymine and uracil) (Extended Data Table 6), were identified in an acid extract by high-performance liquid chromatography with electrospray ionization and high-resolution mass spectrometry (HPLC/ESI-HRMS; Methods and Supplementary Figs. 11–13). Many of these N-heterocycles were also detected in aggregate material using wet chemistry pyGC-QqQ-MS (Supplementary Fig. 14).

## Discussion

### Evidence for extraterrestrial soluble organic matter

The diversity of SOM in the Bennu methanol extract (Fig. 2) is inconsistent with terrestrial biology, which has a much simpler distribution^17^. The large ^15^N enrichment (δ^15^N = +180‰; Extended Data Table 1) in the hot-water extract that consisted of ammonia, amines, amino acids, N-heterocycles and other N-containing molecules falls well outside the δ^15^N terrestrial organics range of –10‰ to +20‰ (ref. ^18^). The complex distribution of amines, carboxylic acids and mostly racemic amino acids, including several non-protein amino acids that are rare or non-existent in biology (Extended Data Tables 2–5), strongly supports an extraterrestrial origin of these molecules. The violation of Chargaff’s rules (1:1 ratio of purine and pyrimidine bases should exist in the DNA of any organism) and diversity of N-heterocycles, including biologically uncommon molecules (Extended Data Table 6), also indicate a non-terrestrial origin.

### Bennu’s volatile-rich nature compared with other astromaterials

The Bennu aggregate samples analysed in this study had a higher mass-weighted average abundance of total C and N than previously studied CI and CM meteorites^19^ and aggregate samples from Ryugu (Supplementary Table 3). This high volatile content may be related to the formation environment and/or alteration history of Bennu’s parent body. Although Bennu's mineralogy and elemental composition are similar to those of the extensively altered CI1 chondrites^5^, the bulk H and N isotopic compositions (Extended Data Table 1 and Supplementary Fig. 3) suggest a closer affinity to less aqueously altered type-2 chondrites, such as Tagish Lake and Tarda.

The diversity of SOM in the Bennu aggregates is comparable with that in Ryugu samples and the CM2 meteorite Murchison^17,20^, though with a lower mass range and carbon oxidation state than Murchison (Fig. 2 and Extended Data Fig. 1). The nitrogen-rich composition of the Bennu aggregates analysed so far contrasts with the sulfur-rich chemistry of the Ryugu samples^20^, reflecting low-temperature aqueous processing on Bennu’s parent body and a nitrogen-rich organic chemistry distinct from that of the most aqueously altered CI and CM chondrites.

**Fig. 5:**
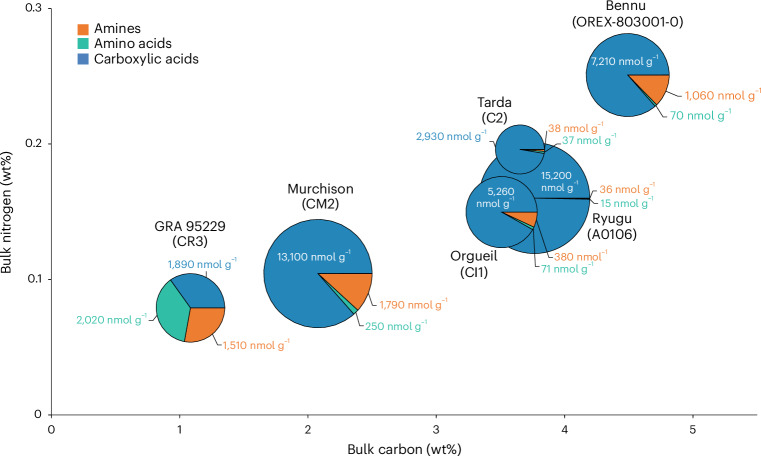
Distribution and total abundances of amines, amino acids and carboxylic acids in Bennu (OREX-803001-0) compared with Ryugu (A0106) and selected CCs. Relative percentages of amines (orange), amino acids (green) and carboxylic acids (blue) are given in the individual pie charts with their overall size proportional to the total sum of the abundances of the three soluble organic compound classes. The pie charts are plotted on a bulk N versus C diagram to illustrate the total C and N abundance differences between the samples in weight percentage. The values for the total sum of the abundances of the molecules detected in each compound class are also given in nanomoles per gram for each pie slice. Although N-heterocycles were also quantified in Bennu and Murchison, these data were excluded from this figure due to their low abundances relative to the other compound classes and incomplete data for the other samples. The N-heterocycle abundance data for Bennu, Murchison, Orgueil and Ryugu are included in Extended Data Table 6. Other water-soluble organic compounds such as aldehydes and ketones, hydroxy acids, cyanides and amides have been identified in CCs^2^ but were not analysed in this study and are therefore also not included in the figure. The amine, amino acid and carboxylic acid data for Murchison and Bennu are from this investigation (Extended Data Tables 2, 3 and 5). Previously published data from Ryugu^20^ and GRA 95229 (ref. ^15^) are also shown. For Orgueil, published amine^58^ and amino acid^59^ data were used, whereas the carboxylic acid data are from this study (Extended Data Table 5). The data for Tarda from Extended Data Tables 2, 3 and 5 were also measured in this study. Source Data

The water-extracted ammonia abundance that we measured for Bennu was 12 times higher than in Murchison and 75 times higher than in Ryugu (Fig. 1 and Extended Data Table 2). It is exceeded only by that of the CR2 Graves Nunataks (GRA) 95229 (ref. ^16^) and the CI1 Orgueil^21,22^. A different Orgueil sample extract^16^ had much lower free ammonia abundances compared with Bennu (Fig. 1). Because hydrothermal treatment at 300 °C and 100 MPa releases additional insoluble organic matter-bound ammonia from these CCs^16^, the abundance of ammonia in the Bennu hot-water extract is likely to represent a lower limit (Extended Data Table 2). Ammonium salts have also been identified in comet 67P/Churyumov–Gerasimenko^23,24^ and the dwarf planet Ceres^25^. Carboxylic acids are typically among the most abundant soluble organic compound classes in CCs (Fig. 5)^26^, and these molecules could have served as the counterions to any ammonium salts in Bennu (for example, ammonium formate as observed in comet 67P (refs. ^23,24^)).

The C_1_–C_6_ amine distribution follows the trend of decreasing concentration with increasing size observed in CI1, CM2 and C2_ung_ chondrites (Extended Data Table 2). However, the higher overall abundance and broader distribution of amines compared with Ryugu and Orgueil (Extended Data Table 2) could be explained by a lower degree of aqueous activity during organic synthesis in Bennu’s parent body. Ryugu samples exhibit a much higher abundance of isopropylamine versus the less thermally stable *n*-propylamine^27^, whereas the straight-chain and branched amine abundances we measured for Bennu were similar to each other (Extended Data Table 2). This observation may also be indicative of less extensive hydrothermal alteration in Bennu’s parent body.

The Bennu hot-water extract displays greater structural diversity of monocarboxylic acids compared with Ryugu with nine C_1_–C_7_ carboxylic acids identified (Extended Data Table 5). Although the Ryugu aggregate has much higher total abundances of formic and acetic acids compared with Bennu, no other monocarboxylic acids were reported in the Ryugu extract above 0.1 nmol g^−1^ (Extended Data Table 5). This difference could be a result of a more acidic pH of the fluids on Ryugu’s parent body^21^ compared with Bennu’s, leading to their evaporation and/or more extensive aqueous alteration, ultimately decomposing or altering carboxylic acids^28^. The structural diversity of carboxylic acids in Bennu is consistent with an origin through stochastic low-temperature free radical reactions on interstellar dust grains^26^. Isotopic analyses of carboxylic acids are needed to further constrain the abiotic origins of these molecules in Bennu.

**Fig. 6:**
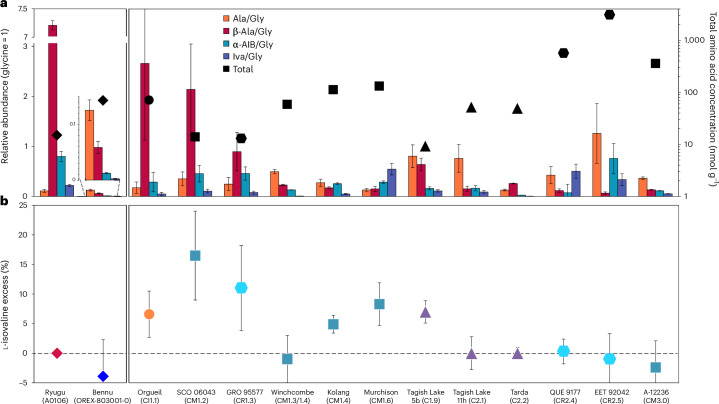
Relative abundances and total concentrations of amino acids and percentage of l-isovaline enantiomeric excesses measured by liquid chromatography mass spectrometry in Bennu (OREX-803001-0) compared with Ryugu (A0106) and selected CCs. **a**, The relative molar abundances (left *y* axis, glycine = 1) of alanine (Ala), β-alanine (β-Ala), α-aminoisobutyric acid (α-AIB) and isovaline (Iva) are shown as coloured bars with uncertainties determined by standard error propagation of the absolute errors. The total concentrations of identified C_2_ to C_6_ amino acids in the 6 M HCl-hydrolysed, hot-water extracts of the samples are designated by the black data points (right *y* axis, logarithmic scale). **b**, l-isovaline excesses (%l_ee_ = %l − %d) and associated standard errors were calculated from the average individual abundances of d- and l-isovaline in the same extracts. The amino acid data for Bennu are the average values reported in Extended Data Tables 3 and 4. Published amino acid data from Ryugu, Orgueil, SCO 06043, GRO 95577, Murchison, Tagish Lake 5b and 11h, QUE 99177, EET 92042 and A-12236 are shown for comparison^20,27,32,59,60^. The Winchcombe, Kolang and Tarda-RV data are newly published here. The CCs are ordered from least to most aqueously altered (right to left) as inferred from their petrologic type assignments shown in parentheses based on the abundance of H in OH/H_2_O (ref. ^56^). The colours and symbols used were selected to differentiate between asteroids Bennu and Ryugu, and the different CC groups (CI, CM, C2_ung_ and CR). Source Data

The total abundance of identified C_2_ to C_6_ protein and non-protein amino acids in the Bennu hot-water extract (~70 nmol g^−1^) was lower by a factor of 3.6 than that in Murchison but 4.7 times higher than in Ryugu extracts (Fig. 6 and Extended Data Table 3). Bennu’s total amino acid abundance resembles that of CI1 and some less altered CM2 and C2_ung_ chondrites (Fig. 6a). However, the amino acid distribution is dominated by glycine, with lower relative abundances of α-alanine, β-alanine, α-aminoisobutyric acid and isovaline compared with CCs (Fig. 6a). The high relative abundances of glycine and racemic mixtures of most α-amino acids suggest a formation by means of HCN polymerization and/or Strecker-cyanohydrin synthesis during aqueous alteration in Bennu’s parent body^2^. However, alternate amino acid formation mechanisms^2,29^ are required to explain the formation of the β-, γ- and δ-amino acids observed in the hot-water extract (Extended Data Fig. 2 and Extended Data Table 3).

The low β-alanine/glycine ratio (~0.08) that we measured for Bennu is unexpected based on trends observed in CCs (Fig. 6a) and Bennu’s CI-like elemental composition and mineralogy^5^. Higher β-alanine/glycine ratios, such as those found in type-1 chondrites and Ryugu samples (>2.7; Fig. 6a), align with extensive hydrothermal alteration, whereas the lower ratio in Bennu is closer to the less aqueously altered type-2 chondrites (Fig. 6a).

Bennu’s amino acid distribution indicates a distinct chemical composition and/or lower-temperature aqueous alteration history of its parent body compared with those of Ryugu and the aqueously altered CCs. Future analyses of the distribution and stable isotopic compositions of amino acids in Bennu samples, including their precursors and related structures, will provide further insight into the formation and evolution of these prebiotic molecules.

The measurement of racemic isovaline (Fig. 6b) and other amino acids, within analytical error, was also unexpected (Extended Data Table 4). Based on the evidence for extensive water activity in Bennu’s parent body^5,30,31^, we predicted that Bennu samples would show some l-isovaline excess, following the empirical trend of higher extraterrestrial l-isovaline excesses in more aqueously altered CCs^13^. Furthermore, substantial l excesses in aspartic and glutamic acids (up to ~60%) measured in some lithologies of the Tagish Lake meteorite and attributed to amplification by crystallization of conglomerate-forming amino acids during parent body aqueous alteration^32^, were not observed in Bennu aggregate either (Extended Data Table 4). The source of the meteoritic l-amino acid enrichments remains a mystery. At least for now, the lack of any amino acid enantiomeric excesses of confirmed extraterrestrial origin in the Bennu material analysed here, as well as in samples from Ryugu^20,27^ and some lithologies of Tagish Lake and Tarda (Extended Data Table 4), challenges the hypothesis that the emergence of left-handed protein-based life on Earth was influenced by an early Solar System bias toward l-amino acids^13^.

The total abundance of N-heterocycles identified in Bennu samples (~5 nmol g^−1^; Extended Data Table 6) is 5–10 times higher than reported in Ryugu^33^ and Orgueil^34^. The elevated abundances and more complex distribution of N-heterocycles again may reflect a lower degree of hydrothermal alteration in Bennu’s parent body during organic synthesis compared with Ryugu’s, which is consistent with trends that have been observed in aqueously altered CI and CM chondrites^34^. However, the ratio of purines to pyrimidines is much lower in the Bennu extract (0.55; Extended Data Table 6) than in Murchison (~2.8) and Orgueil (~1.1). This elevated abundance of pyrimidines over purines may be related to differences in the parent bodies’ chemical composition, formation pathways and/or aqueous alteration histories. N-heterocycles can be readily synthesized from ammonia and formaldehyde, especially under alkaline conditions^35^. Because pyrimidines are preferentially formed over purines in interstellar ice–analogue irradiation experiments^36^, it is also possible that Bennu’s N-heterocycles and/or their chemical precursors were inherited from a cold molecular cloud environment. The unusual richness of N-heterocycles may be of relevance for prebiotic chemistry. Further studies of nucleobase chemistry in samples returned from Bennu, from precursors to nucleic acids, are warranted.

### Bennu’s origin and implications for prebiotic chemistry

The elevated volatile content, the large ^15^N enrichments of ammonia and other water-soluble N-containing molecules and the high abundance of N-rich isotopically anomalous organic matter^37^ observed in the Bennu samples suggest that the parent body accreted ices from a reservoir in the outer Solar System, where ammonia ice was stable (beyond Jupiter’s current orbit). Dynamical simulations predict that Bennu derived from a secondary parent body in the inner main belt (2.1–2.5 au) that broke up 730–1,550 Myr ago^38^. The parent body may have originated in the outer Solar System, perhaps emplaced into the asteroid belt during giant-planet migrations, as has previously been proposed for CI chondrites and Ryugu^39^. Alternatively, ices may have migrated inward by means of pebble drift, a process where small, icy pebbles drift inward from the outer Solar System and accrete onto forming planetesimals in the asteroid belt. This mechanism would allow material from more distant, colder regions, where ammonia and other volatile ices are stable, to be incorporated into bodies forming closer to the Sun^40^.

B-type asteroids such as Bennu^41^, so named for their blue spectral slopes, and other small bodies that emit particles^42^ have been hypothesized^43^ to be fragments of extinct comets sampling a continuum of objects, from dry planetesimals that formed close to the Sun to volatile-rich icy bodies that formed well beyond the water snow line. There is some evidence of low-temperature aqueous activity in comets, including the spectroscopic detection of hydrated minerals and carbonates in the impact ejecta of comet 9P/Temple 1 by the Spitzer Space Telescope^44^ and cubanite in samples from comet 81P/Wild^45^. Nevertheless, the phyllosilicate-dominated bulk mineralogy of Bennu samples^5^ and the spacecraft observations of metre-sized carbonate-rich veins^31^ on Bennu both imply large-scale hydrothermal activity for millions of years that may not be consistent with a cometary parent body origin.

Alternatively, the presence of ammonium and carbonate salts, high organic carbon content and evidence of rock-fluid interactions observed in Ceres^25,46,47^ suggest that Bennu may consist of fragments of a Ceres-like primitive icy body that experienced extensive low-temperature aqueous activity. Petrologic data from Bennu samples indicate that late-stage fluid in the parent body sequentially precipitated evaporite minerals, starting with Ca and Mg carbonates, progressing to phosphates, followed by Na carbonates and concluding with halides and sulfates^48^. These minerals strongly imply alkaline pH, substantial concentrations of dissolved inorganic carbon and fluid temperatures below ~55 °C (ref. ^48^). This dynamically changing environment in Bennu’s parent body is likely to have fostered intricate interactions among brine fluid chemistry, soluble organics and freshly exposed mineral surfaces. High concentrations of ammonium salts in Bennu’s parent body could have created liquid brines at very low temperatures (NH_3_–H_2_O eutectic of 176 K), and thus provided an aqueous environment for organic chemistry to continue even as the abundances of short-lived radionuclides responsible for internal heating were exhausted^49^. For example, a suite of amino acids dominated by glycine and the purines adenine and guanine were produced in dilute NH_4_CN kept at 195 K for 25 yr (ref. ^50^). Eutectic freezing, phyllosilicate catalysis, low-temperature template polymerization and Mg salts are employed in the laboratory polymerization of activated nucleotides^51^.

Additional analyses of Bennu samples, coupled with laboratory analogue experiments and future sample return missions from a comet and Ceres, will be important to further understand the origin and evolution of prebiotic organic matter in Bennu and potential chemical links between volatile-rich asteroids and primitive icy bodies. Regardless of their origins, asteroids such as Bennu could have been a source of N-rich volatiles and compounds of biological importance, including ammonia, amino acids, nucleobases, phosphates and other chemical precursors that contributed to the prebiotic inventory that led to the emergence of life on Earth.

## Methods

Before these investigations, validation of the analytical methods used in this study were performed on Murchison and Sutter’s Mill as part of OSIRIS-REx sample analysis readiness tests^52–54^.

### Samples used in this investigation

The Bennu aggregates studied (Supplementary Fig. 1) consisted of a mixture of mostly fine (<100 μm) to intermediate (100–500 μm) sized particles with some coarse (>500 μm) grains dominated by hydrous silicate minerals (~80% phyllosilicates by volume) with lower abundances (≤10%) of sulfides, magnetite, carbonates, anhydrous silicates (olivine and pyroxene) and other minor phases^5^. The Bennu sample nomenclature as well as the detailed processing and analytical flow of the aggregate samples are summarized in Supplementary Table 1 and Supplementary Fig. 2.

Two aggregate samples (OREX-500002-0 and OREX-500005-0) that were included as part of the ‘quick-look’ (QL)^5,12^ analyses were removed from the avionics deck surface, weighed and then containerized under N_2_ in the curation glovebox^55^ at the NASA Johnson Space Center (JSC). OREX-500002-0 (~22 mg) consisted primarily of dark fines and some intermediate-sized particles, with some bright and highly reflective particles, and numerous (>5) white fibres thought to be derived from the sample return capsule aluminized Kapton multilayer insulation filled with fibreglass. The sample was sealed under N_2_ between two glass concavity slides and shipped from NASA JSC to the Carnegie Institution for Science (CIS). This sample was inspected under an optical microscope at CIS, and the fibres were physically removed from the sample with organically clean stainless-steel tweezers. A 1.1 mg subsample of the aggregate (OREX-501029-0) was then transferred from the concavity slide to a separate glass pyrolysis tube at CIS and carried by hand to the NASA Goddard Space Flight Center (GSFC) for targeted amino acid and N-heterocycle analyses using wet chemistry and pyGC-QqQ-MS (more detail about the method in the ʽCoordinated analyses of organics in the aggregate samplesʼ section below). The remaining ~20 mg were further split into multiple subsamples for elemental and stable isotopic analyses of bulk carbon, nitrogen and hydrogen, using an EA-IRMS instrument at CIS (details in the next section).

OREX-500005-0 consisted of mostly dark fines with an average grain size <100 μm, but with some particles up to ~500 μm. Some bright and highly reflective particles were also present in this aggregate sample; however, no fibres were observed. OREX-500005-0 (~88 mg) was sealed under N_2_ inside a glass vial with a Viton stopper and crimped aluminium lid. It was subsampled to obtain OREX-501006-0 (<1 mg) for coordinated optical and UV fluorescence microscopy and μ-L^2^MS analyses at NASA JSC (see ʽCoordinated analyses of organics in the aggregate samplesʼ section for more detail about the methods below).

Bulk material from the touch-and-go sample acquisition mechanism (TAGSAM) was subsampled to obtain TAGSAM aggregate (TA) samples OREX-800031-0 (~52 mg) and OREX-800044-0 (~109 mg). OREX-800031-0 was shipped from JSC to GSFC in a glass concavity slide (Supplementary Fig. 1) that was hermetically sealed under high purity N_2_ inside an Eagle Stainless container. The container was opened inside an ISO 5 HEPA-filtered laminar flow hood housed in an ISO ~7 white room and the aggregate sample was subsampled and distributed for multiple analyses following a coordinated analysis scheme (Supplementary Fig. 2). A similar mass of a powdered sample of the CM2 Murchison from the University of Illinois, Chicago, and a powdered sample of fused silica (FS-120, HP Technical Ceramics) that had been previously ashed at 500 °C in air overnight to remove organic contaminants were also processed in parallel with the Bennu OREX-800031-0 aggregate sample. Procedural solvent blanks were also processed in parallel and analysed. OREX-800044-0 was shipped from JSC to Hokkaido University in Japan in a glass concavity slide (Supplementary Fig. 1) that was hermetically sealed under high purity N_2_ inside an Eagle Stainless container. It was further subsampled in a glass concavity slide to OREX-800044-101 (17.75 mg) and shipped to Kyushu University in Japan.

### Bulk C, N and H contents and their isotopic compositions

The elemental abundances of carbon (C, wt%), nitrogen (N, wt%) and hydrogen (H, wt%) and their isotopic compositions δ^13^C, in parts per thousand relative to the Vienna Peedee Belemnite, δ^15^N, in parts per thousand relative to Earth atmospheric nitrogen, and δD, in parts per thousand relative to the Vienna Standard Mean Ocean Water isotope reference, were analysed in subsamples of OREX-500002-0 and OREX-803007-0 and in a sample of the Murchison meteorite processed in parallel with OREX-803007-0 at GSFC after extraction in water at 100 °C for 24 h. These measurements were made with: (1) a Thermo Scientific Delta V^plus^ isotope ratio mass spectrometer (IRMS) connected to a Carlo Erba elemental analyser (EA) by means of a Conflo III interface for C and N analyses; and (2) a Thermo Scientific Delta Q IRMS connected to a Thermo Finnigan Thermal Conversion elemental analyser by means of a Conflo IV interface for H analyses using previously described methods^56^. The subsample masses used for H and C + N analyses were ~1–1.5 mg and 5.5 mg, respectively. Subsamples were placed in an Ar-flushed glovebox and heated to 120 °C for 48 h to reduce the amount of adsorbed atmospheric water before analysis (Supplementary Table 2). The reported uncertainties for the elemental and isotopic analyses correspond to a 1σ standard deviation, which was determined based on either replicate analyses of standards or analyses of at least two aliquots of individual samples, with the larger error reported.

Small aliquots (~2.5% of total extracted volume) of the hot-water extracts from TA OREX-803001-0 (designated split OREX-803001-112) and the parallel processed Murchison meteorite were transferred to separate tin capsules, acidified with 2 μl 6 M HCl, and then evaporated to dryness under vacuum at room temperature in a Labconco CentriVap Concentrator at GSFC. The capsules were crimped and analysed in series along with appropriate procedural blanks and standards using the nano EA-IRMS instrument at Pennsylvania State University (PSU) to determine the total C and N abundances as well as the δ^13^C and δ^15^N values following published methods^57^. These analyses were performed after verification of the analytical method with the predicted concentration of ammonia in the TA water extract and corresponding volume of NH_4_OH. The nano EA-IRMS system at PSU employed a Flash IRMS elemental analyser that was coupled by means of a ConFlo IV Universal Interface to a Thermo Scientific Delta V^plus^ Plus IRMS with a universal triple collector. Additional description of the nano EA-IRMS data processing methods and mass balance calculations can be found in the Supplementary Information.

### Coordinated analyses of organics in the aggregate samples

OREX-800031-0 was subsampled for multiple analyses (Supplementary Fig. 2). OREX-803007-0 (23.6 mg) was allocated for bulk H, C and N measurements using EA-IRMS analyses at CIS. OREX-803006-0 (3.3 mg) was allocated for non-targeted molecular profiling of soluble organics in a methanol extract using FTICR-MS at Helmholtz-Zentrum in Munich, Germany. OREX-803004-0 (1.0 mg) was heated at 85 °C for 1.5 h in a sealed pyrolysis tube containing a 5 μl solution (4:1 v/v) of *N*-(*tert*-butyldimethylsilyl)-*N*-methyl-trifluoroacetamide and *N,N*-dimethylformamide and the sample was then analysed directly for the *N*-(*tert*-butyldimethylsilyl)-*N*-methyl-trifluoroacetamide derivatives of amino acids and N-heterocycles by pyGC-QqQ-MS at NASA GSFC. The remaining mass of OREX-803001-0 (25.6 mg) was flame-sealed in a glass ampoule with 1 ml Milli-Q ultrapure water (18.2 MΩ, <3 ppb total organic C) and heated at 100 °C for 24 h.

After water extraction, OREX-803001-0 was centrifuged (5 min at 3,000 rpm), and the water supernatant was separated from the solid residue. Some of the solid residue (OREX-803001-103, 22.9 mg) after water extraction was dried under vacuum at room temperature and sent to CIS for bulk H, C and N measurements. 17.5% of the OREX-803001-0 hot-water extract was analysed directly for free ammonia, hydrazine, aliphatic amines and protein amino acids. These analyses were performed by AccQ•Tag derivatization and liquid chromatography with UV fluorescence detection and either time-of-flight mass spectrometry (LC-FD/ToF-MS) or triple quadrupole mass spectrometry.

Approximately 2.5% of the OREX-803001-0 water extract was analysed by nano EA-IRMS for total C and N at PSU, as previously described. The remaining 80% of the OREX-803001-0 water extract was split equally, with 40% of the water extract desalted by cation exchange chromatography followed by *o*-phthaldialdehyde/*N*-acetyl-l-cysteine (OPA/NAC) derivatization and analysis using both LC-FD/ToF-MS and liquid chromatography with UV fluorescence detection and high-resolution mass spectrometry (LC-FD/HRMS) to determine the free amino acid abundances in the extract. The other 40% was dried, acid-hydrolysed under 6 M HCl vapour at 150 °C for 3 h and then desalted to determine the average total (free + bound) amino acid abundances using both LC-FD/ToF-MS and LC-FD/HRMS. We also measured the distribution and abundances of the 2-pentanol derivatives of free mono- and dicarboxylic acids in the water wash collected during desalting (cation exchange) of the non-hydrolysed water extract of Bennu (OREX-803001-0) using GC-QqQ-MS.

A separate 17.75 mg aggregate sample (OREX-800044-101), subsampled from Bennu OREX-800044-0, was extracted in HCl (Tama Chemicals Co., Ltd.) and analysed for N-heterocycles using HPLC/ESI-HRMS at Kyushu University in Japan. A 14.4 mg ashed sample of sea sand (FUJIFILM Wako Pure Chemical Corporation; 30–50 mesh) was used as a processing blank for OREX-800044-101. Procedural solvent blanks were also processed in parallel and analysed.

A small subsample (<1 mg) of the QL aggregate (OREX-501006-0) was prepared at NASA JSC for coordinated analysis by optical and UV fluorescence microscopy and μ-L^2^MS. Approximately a dozen grains of the QL aggregate were transferred to an infrared grade potassium bromide (KBr) window and gently pressed into the KBr surface using an optical grade sapphire window. The sample mount was imaged optically and under UV fluorescence using an Olympus BX-60 microscope equipped with a tungsten halogen and Hg-arc illumination sources. Native fluorescence images were obtained using 330–385 nm excitation and 420 nm long-pass emission filters. After optical and UV fluorescence imaging, the sample was transferred to a μ-L^2^MS instrument and in situ mass spectra were acquired at a 5 μm spatial resolution using an infrared laser (CO_2_; 10.6 mm) for desorption, a vacuum ultraviolet laser (Nd:YAG 9th harmonic; 118 nm) for photoionization and a reflectron time-of-flight mass spectrometer for mass analysis. Additional details of the imaging and μ-L^2^MS analyses can be found in the Supplementary Information.

## Supplementary information

Supplementary Information Supplementary Discussion and Refs. 65–102, Figs. 1–15 and Tables 1–14.

## Source data

Source Data Fig. 1 Excel file containing all data points used to generate the plot shown in Fig. 1.

Source Data Fig. 2 Excel file containing all data points used to generate the plots shown in Fig. 2a–e.

Source Data Fig. 3 Excel file containing all data points used to generate the plots shown in Fig. 3a–d.

Source Data Fig. 4 Excel file containing all data points used to generate the plots shown in Fig. 4a.

Source Data Fig. 5 Excel file containing all data points used to generate the plot shown in Fig. 5.

Source Data Fig. 6 Excel file containing all data points used to generate the plots shown in Fig. 6a,b.

Source Data Extended Data Fig. 1 Excel file containing all data points used to generate the plots shown in Extended Data Fig. 1a–d.

Source Data Extended Data Fig. 2 Excel file containing all data points used to generate the plots shown in Extended Data Fig. 2.

## Figures and Tables

**Extended Data Table 1:**
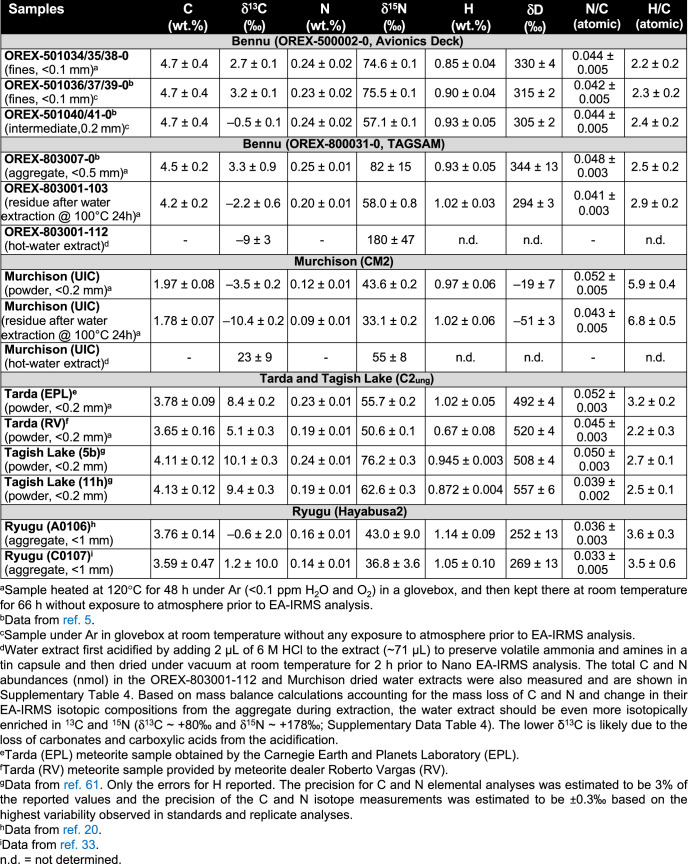
Total C, N, and H contents and stable isotopic compositions of Bennu aggregate samples, compared with selected CCs and Ryugu samples (A0106 and C0107)

**Extended Data Table 2:**
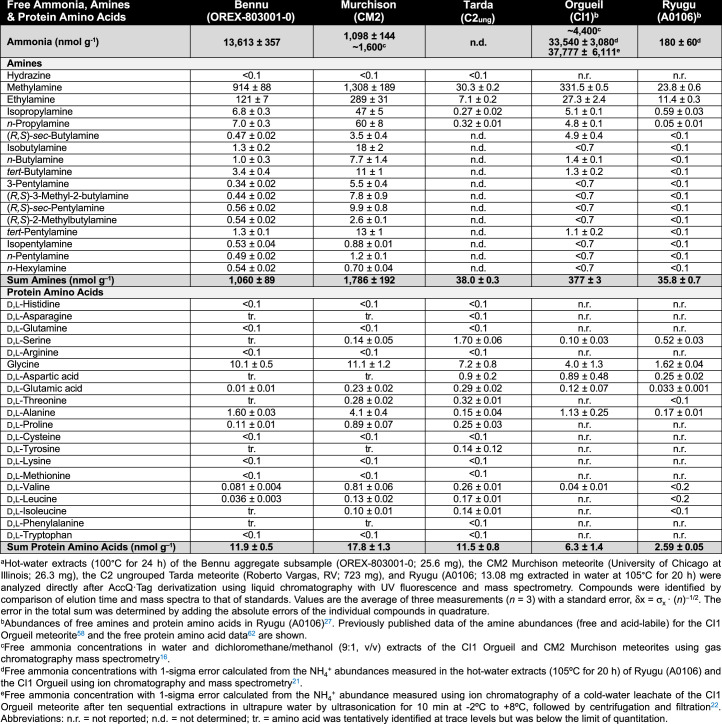
Blank-subtracted free abundances of ammonia, amines, and amino acids, as measured by LC-FD/ToF-MS, in the hot-water extract of Bennu (OREX-803001-0), compared with selected CCs and Ryugu (A0106)^a^

**Extended Data Table 3:**
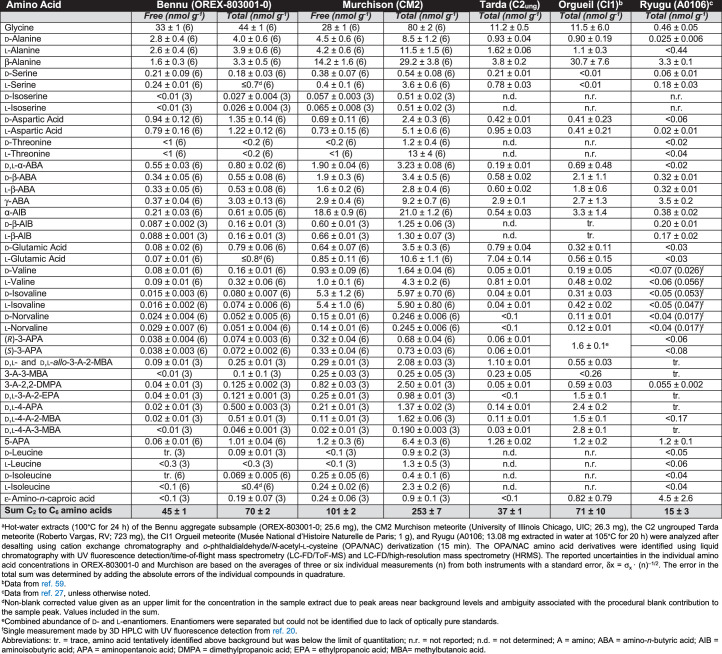
Free and total amino acid abundances, as measured by LC-FD/ToF-MS and LC-FD/HRMS, in the hot-water extract of Bennu (OREX-803001-0), compared with selected CCs and Ryugu (A0106)

**Extended Data Table 4:**
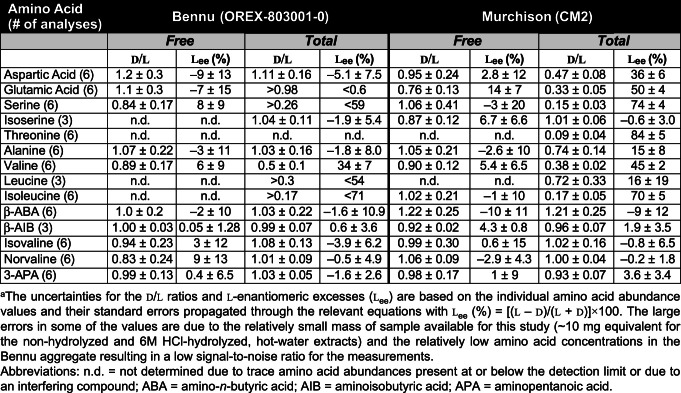
Summary of the d/l ratios and corresponding l-enantiomeric excesses (l_ee_) of protein and non-protein amino acids measured in Bennu (OREX-803001-0) and Murchison hot-water extracts

**Extended Data Table 5:**
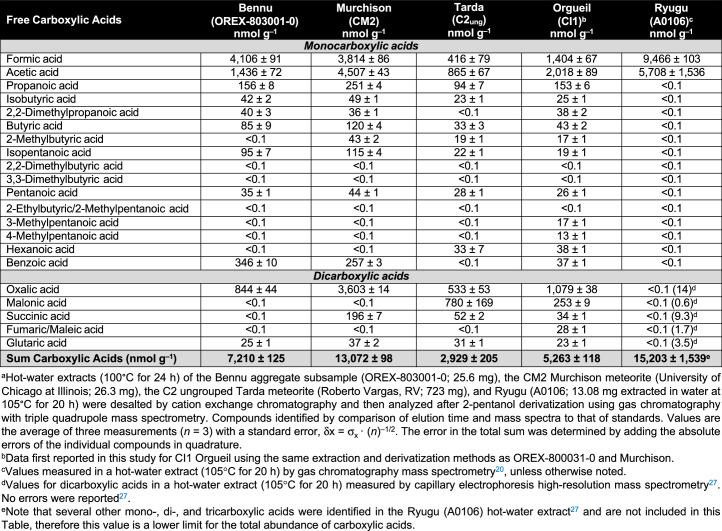
Blank-subtracted free abundances of carboxylic acids identified by GC-QqQ-MS analyses of the hot-water extract of Bennu (OREX-803001-0), compared with selected CCs and Ryugu (A0106)^a^

**Extended Data Table 6:**
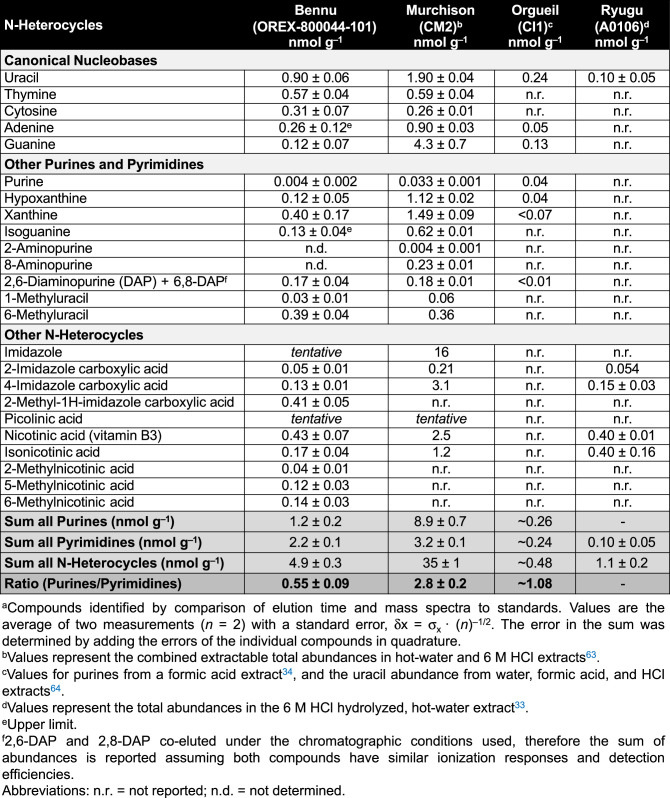
Blank-subtracted abundances of N-heterocycles identified by HPLC/HRMS analyses of a 6 M HCl extract of Bennu (OREX-800044-101), compared with selected CCs and Ryugu (A0106)^a^

## Data Availability

The instrument data supporting the experimental results in this study are available at https://astromat.org at the DOIs given in Supplementary Table 14 and/or within the manuscript and its Supplementary Information. Source data are provided with this paper.
