# Supplementary material for: Abundant ammonia and nitrogen-rich soluble organic matter in samples from asteroid (101955) Bennu
Source: Nat Astron. 2025 Jan 29;9(2):199–210. doi: 10.1038/s41550-024-02472-9 (PMC11842271; doi:10.1038/s41550-024-02472-9)
Supplement: Supplementary file 1 — Supplementary Discussion and Refs. 65–102, Figs. 1–15 and Tables 1–14. [file 41550_2024_2472_MOESM1_ESM.pdf]

nature astronomy

Supplementary information https://doi.org/10.1038/s41550-024-02472-9

Abundant ammonia and nitrogen-rich soluble organic matter in samples from asteroid (101955) Bennu

In the format provided by the authors and unedited

Supplementary information

Materials and analytical methods

Brief overview of the composition of the Bennu aggregates. The Bennu aggregate samples studied consisted of a mixture of mostly fine (<100 μm) to intermediate (100–500 μm) sized particles with some coarse (>500 μm) grains dominated by hydrous silicate minerals (~80% phyllosilicates by volume) with lower abundances (≤ 10%) of sulfides, magnetite, carbonates, anhydrous silicates (olivine and pyroxene), and minor phases including phosphates and phosphides consistent with extensive aqueous alteration on the Bennu parent body[5]. The bulk elemental compositions of the Bennu aggregate samples are similar to the Ryugu aggregate materials and CI carbonaceous chondrites. However, enrichments of some fluid mobile elements in the "quick-look" (QL) samples that are distinct from solar composition may indicate a unique chemical component in the avionics deck material whose source has yet to be identified[5]. The insoluble organic matter in the Bennu aggregate is present in the form of plates and veins, carbonaceous–mineral aggregates, and discrete micrometer-scale nanoglobules that are abundant and widespread throughout the Bennu mineral matrix[5].

Bennu samples and controls. Images of two of the Bennu aggregate samples analyzed as part of this investigation are shown in Supplementary Fig. 1. The sample nomenclature and details of the processing and analytical flow of the Bennu aggregate samples are summarized in Supplementary Table 1 and Supplementary Fig. 2. Two aggregate samples (OREX-500002-0 and OREX-500005-0) that were studied as part of the QL analyses were removed from the avionics deck surface, weighed, and then containerized under N_2 in the curation glovebox. OREX-500002-0 (22 mg) consisted primarily of dark fines (<100 μm) and some intermediate-sized particles (100–500 μm), with some bright and highly reflective particles, and numerous (>5) fibers thought to be derived from spacecraft blanketing material. The sample was sealed under N_2 between two glass concavity slides and shipped from the NASA Johnson Space Center (JSC) to the Carnegie Institution for Science (CIS). This sample was inspected under an optical microscope at CIS, and the fibers were physically removed from the sample with ashed tweezers. A 1.1 mg subsample (OREX-501029-0) of the aggregate was then transferred from the concavity slide to an ashed glass pyrolysis tube and then hand carried to the NASA Goddard Space Flight Center (GSFC) for wet chemistry and analysis by pyrolysis gas chromatography coupled to triple quadrupole mass spectrometry (*py*-GC-QqQ-MS). The remaining ~20 mg were further split into multiple subsamples for elemental and isotopic analyses of total carbon, nitrogen, and hydrogen using an elemental analyzer–isotope ratio mass spectrometer (EA-IRMS). A second aggregate sample collected from the avionics deck (OREX-500005-0) consisted of mostly dark fines with an average grain size <100 μm, but with some particles up to ~500 μm. Some bright and highly reflective particles were also present in the aggregate sample. OREX-500005-0 was sealed under N_2 inside a glass vial with a Viton stopper and crimped aluminum lid. Although the initial mass of the sample was 88 mg, only ~1 mg of the sample was used as part of a coordinated analysis of organic matter at NASA Johnson Space Center (JSC).

A ~52 mg aggregate sample (OREX-800031-0) removed from the OSIRIS-REx TAGSAM sample collector consisting of a mixture of fine- to intermediate-sized particles was shipped under N_2 to GSFC in between two glass concavity slides (Supplementary Fig. 1) where it was subsampled for multiple analyses per the analytical scheme shown in Supplementary Fig. 2. A similar mass of a crushed sample of the Murchison CM2 (Mighei-type) carbonaceous chondrite from the University of Illinois Chicago (UIC) and a powdered sample of fused silica (FS-120, HP Technical Ceramics, Sheffield, UK) that had been previously ashed at 500°C in air overnight to remove organic contaminants were processed in parallel with the Bennu aggregate. A 23 mg aliquot of the Bennu sample (OREX-803007-0) was allocated for bulk H, C, and N analyses at CIS; 3.3 mg was dedicated for nontargeted molecular profiling of soluble organics using Fourier-transform ion cyclotron resonance mass spectrometry (OREX-803006-0) at Helmholtz Zentrum in Munich, Germany; and 1 mg (OREX-803004-0) was allocated for wet-chemistry *py*-GC-QqQ-MS analysis at NASA GSFC for additional characterization of the SOM composition. Most of the remaining mass of each sample was extracted in hot water at 100°C for 24 h, and aliquots of the water supernatant were split and analyzed for amino acids, aliphatic amines, and ammonia using liquid chromatography with UV fluorescence detection and time-of-flight mass spectrometry (LC-FD/ToF-MS) and carboxylic acids using GC-QqQ-MS. A 25.6 mg subsample (OREX-803001-0) of the Bennu aggregate OREX-800031-0 was used for the hot-water extraction, and in parallel, a 26.3 mg sample of Murchison and a 27.4 mg sample of the FS-120 were also processed. The solid residues of both Murchison and the Bennu aggregate (OREX-803001-103) after hot-water extraction were dried under vacuum and analyzed for bulk H, C, and N by EA-IRMS at CIS. The total C and N abundance and isotopic composition of a portion of the Bennu water extract (OREX-803001-112) and Murchison that was acidified with HCl and dried under vacuum was determined using the nano EA-IRMS instrument at Penn State University (PSU). The details of the hot-water extraction and analytical procedures used in this investigation are described in the section that follows.

A separate 17.75 mg sample of Bennu (OREX-800044-101), subsampled from Bennu aggregate OREX-800044-0 from the TAGSAM sample collector head (Supplementary Fig. 1), was extracted in HCl and analyzed for N-heterocycles using high performance liquid chromatography with high-resolution mass spectrometry (HPLC-HRMS) at Kyushu university in Japan. A 14.4 mg ashed sample of sea sand (FUJIFILM Wako Pure Chemical Corporation, Japan; 30–50 mesh) was used as a processing blank for OREX-800044-101. Procedural solvent blanks were also processed in parallel and analyzed.

Samples of the carbonaceous chondrites Winchcombe (CM1.3/1.4, UA 2925,12, mass 171.5 mg), Kolang (CM1.4, ASU 2147, mass 450.6 mg) and the C2 ungrouped Tarda (C2.2, mass 723.0 mg) that had been crushed using ashed ceramic mortar and pestles were also extracted in hot water and analyzed separately for amino acids using the same extraction and LC-FD/ToF-MS analytical procedures as the Bennu TA subsample OREX-803001-0 previously described. Separate aliquots (~15–20 mg) of each meteorite powder were sent to the Carnegie Institution for Science for bulk H, C, and N analyses using the same methods described in the following section.

Analytical measurements and discussion

EA-IRMS analysis of the bulk C, N, and H abundances and isotopic compositions in the solid samples

Sample preparation and analytical details. The elemental and isotopic analyses of the bulk H, C, and N were carried out at the Earth and Planets Laboratory, following previously established protocols[19,56,65]. Three samples were analyzed: (i) one recovered from the avionics deck (OREX-500002-0, 19 mg) as part of the quick-look phase of the Sample Analysis Plan[12], and (ii) (one retrieved from inside the OSIRIS-REx TAGSAM sample collector (OREX-803007-0, 23 mg; parent sample OREX-800031-0), and (iii) the dried solid residue of Bennu aggregate from the sample collector after extraction in water at 100°C for 24 h (OREX-803001-103, 23 mg). The samples consisted of a mixture of fine- to intermediate-sized particles, and subsampling commenced without any further particle size homogenization. However, a subsample of intermediates (~200 μm) was hand-picked from the fines and analyzed separately in the case of OREX-500002-0. The bulk H, C, and N elemental and isotopic compositions of these samples were determined within four days of sample delivery.

Subsamples of each aggregate were weighted into Ag capsules for H (0.877–2.161 mg), or into Sn capsules for C and N analyses (2.281–5.553 mg). Afterwards, they were put in an Ar-flushed glovebox and subjected to 120°C heating for 48 hours in order to decrease the quantity of adsorbed atmospheric H_2O[66,67]. By evaluating the δD composition of bulk Murchison pretreated at 50°C to 200°C in a series of control experiments, the adopted protocol was established. The samples were reweighed and loaded into a Zero-Blank Costech autosampler (for H analysis) while in the glovebox prior to analysis. While Ar was continuously flushing, the autosampler was transferred to the elemental analyzer–isotope ratio mass spectrometer. The samples were exposed to an Ar atmosphere for a duration exceeding 66 hours before being analyzed. A set of subsamples from the OREX-500002-0 were not exposed to the heat treatment. Blank measurements in every analytical run always included Ag and Sn capsules.

The C and N elemental abundances and isotopic compositions of the Bennu bulk aggregate samples were measured with a Thermo Scientific Delta V^{Plus} mass spectrometer interfaced with a Carlo Erba (NA 2500) elemental analyzer. As shown in Supplementary Fig. 3 and Supplementary Table 2, the Bennu aggregate samples are enriched in total C (4.5 to 4.7 wt.%)[5] and total N (0.23 to 0.25 wt.%)[5] compared to the mass weighted average values of all bulk abundances published to date on aggregate samples collected from asteroid Ryugu by the Hayabusa2 mission shown in Supplementary Table 3 (C = 3.81 wt.%, N = 0.13 wt.%)[20,33,68-71] and most unheated petrologic type 1 and 2 carbonaceous chondrites[2,3]. The combination of H and N isotopic enrichments in these Bennu aggregate samples (δD = +305 to 365 ‰, δ^{15}N = +57 to 106 ‰: Supplementary Table 2) is also distinct from the mass weighted averages for Ryugu shown in Supplementary Table 3 (δD = +200 ‰, δ^{15}N = +33 ‰)[20,33,68-71] and most unheated petrologic type 1 and 2 carbonaceous chondrites[19,56].

For the Bennu aggregate bulk H analyses, we used a Thermo Scientific Delta Q mass spectrometer connected to a Thermo Finnigan Thermal Conversion elemental analyzer (TC/EA) operating at 1400°C. N_2, CO_2 and H_2 reference gases were introduced via a Conflo III and Conflo IV interface, respectively. Reference gases and in-house standards (acetanilide, stearic acid) have been calibrated against international standard gases and

solids of known isotopic composition (Oztech Trading Company, Isoanalytical Laboratory, National Bureau of Standards-22, IAEA-60, Standard Mean Ocean Water). By analyzing internal working gas standards during analysis, the internal precision of the measured isotopic ratios and elemental compositions throughout the run was monitored regularly. To calibrate and correct the data, in-house standards, including both liquid and solid materials, were analyzed at regular intervals between samples. An H_3^+ correction determined by H-linearity tests was applied to the H measurements[14]. The reported uncertainties for the elemental and isotopic analyses correspond to a 1σ deviation, which is determined based on either replicate analyses of standards or analyses of at least two aliquots of individual samples, whichever is the larger. The replicate samples were analyzed sequentially to check for sample heterogeneity, and in the case of H, to evaluate small memory effects on δD measurements known to occur with D-enriched samples[19,73]. Blanks were run between different samples to reduce the memory effects. Memory effects were also monitored by in-house standards within the course of an analytical run. There is no memory effect for the C and N analyses[19].

Bulk elemental ratios. The bulk elemental ratios (atomic) for OREX-500002-0 and OREX-803007-0 ranged from N/C ~ 0.04 to 0.05 and H/C ~ 2.2 to 2.5 (Extended Data Table 1). The $\delta^{13}C$ and N/C values suggest that up to ~10% of the total carbon in these Bennu aggregates (~0.45 to 0.47 wt.% C) could be in carbonate[74]. The estimated abundance of carbonate C in these aggregate samples from the EA-IRMS data is in good agreement with an XRD measurement of another Bennu aggregate sample (OREX-500005-0)[5] that contained 3 ± 1 wt.% carbonate (predominately dolomite, $CaMg(CO_3)_2$), which is equivalent to 0.39 ± 0.13 wt.% C from dolomite. The composition of a ~0.2 mm intermediate-sized particle was depleted in ^{13}C ($\delta^{13}C$ = –0.5‰, Extended Data Table 1, Supplementary Table 2) compared to finer grained material from the same sample, revealing some degree of heterogeneity which may be due to a difference in the abundance of carbonate phases between the finer and coarser grained fractions. The significant contributions of H from hydrated minerals in the samples can explain the elevated bulk H/C values in these Bennu samples compared to the predicted H/C values of ~0.3 to 0.6 of the IOM based on the average 3.42 μm band depth from organic rich IR spectra of Bennu[9].

C and N mass balance estimates. The bulk C and N measurements of a Bennu aggregate sample (OREX-803001-103), after extraction in hot water, indicated that the abundances of C and N in the residue were both lower and more isotopically depleted (Extended Data Table 1, Supplementary Table 2) compared to the aggregate sample prior to water extraction. Based on mass balance calculations accounting for the mass loss of bulk C and N and change in the isotopic composition ($\delta^{13}C$ and $\delta^{15}N$ values) of the solid residue after water extraction (Extended Data Table 1), the water extract from the Bennu aggregate should be isotopically enriched in both ^{13}C and ^{15}N, with average $\delta^{13}C$ and $\delta^{15}N$ values of +80 ± 77‰ and +178 ± 86‰, respectively. The same calculations also suggested a heavy average C and N isotopic composition of the Murchison water extract ($\delta^{13}C$ ~ +61‰ and $\delta^{15}N$ ~ +80‰), but they were not as enriched as Bennu. To confirm the predicted C and N isotopic composition of the water extracts based on the mass balance

calculations, we measured the $\delta^{13}C$ and $\delta^{15}N$ values of the water extracts using the nano EA-IRMS instrument at PSU (Supplementary Table 4).

Nano EA-IRMS analysis of C and N abundances and isotopic compositions in the water extracts

Analytical details and data processing methods. Nano EA-IRMS analyses were conducted at PSU following previous methods[57]. Briefly, the nano EA-IRMS system employed a Flash™ IRMS elemental analyzer that was coupled via a ConFlo IV Universal Interface to a DELTA V Plus isotope ratio mass spectrometer with a universal triple collector. The Flash IRMS EA consisted of a Costech zero blank autosampler, a custom-made narrow-bore combination combustion–reduction reactor (18 mm O.D., 10 mm I.D., and 45.4 cm long) filled with WO3 and reduced copper grains and operated at 1,020°C, a custom-made water trap (glass tube, 15 cm long, 3.81 mm I.D.) containing magnesium perchlorate, and a carbonPLOT capillary GC column (15 m, 0.32 mm I.D., and 1.5 μm film thickness).

For any given isotope measurement, the measured value reflects a weighted average of the sample isotope value and the procedural blank:

$$A_{meas}\delta_{meas} = A_S\delta_S + A_B\delta_B\,, \qquad \text{(Equation 1)}$$

where δ_{meas} and A_{meas} are the measured isotope value and peak area, δ_S and A_S are the sample isotope value and peak area, and δ_B and A_B are the isotope value and peak area of the procedural blank. If the size, composition, and variability of the procedural blank can be measured directly, results of individual analyses (δ_{meas}) can be corrected (δ_C) for the blank contribution using experimentally determined values for δ_B and A_B ($\overline{\delta}_B$ and $\overline{A}_B$):

$$\delta_C = \frac{A_{meas}\delta_{meas} - \overline{A}_B\overline{\delta}_B}{A_{meas} - \overline{A}_B}. \qquad \text{(Equation 2)}$$

The blank-corrected isotope value uncertainty depends on the uncertainty in $\overline{A}_B$ and $\overline{\delta}_B$:

$$\sigma^2_{\delta_S} = \frac{1}{N^2}\left(\left(\frac{E^2}{N^2}\right)\left(A_B^2\sigma^2_{A_{meas}} + A^2_{meas}\sigma^2_{A_B}\right) + A^2_{meas}\sigma^2_{\delta_{meas}} + A_B^2\sigma^2_{\delta_B}\right), \qquad \text{(Equation 3)}$$

where $N = A_{meas} - A_B$ and $E = \delta_{meas} - \delta_B$.

For direct measurement of the blank, with A_B and δ_B calculated as the mean of *n* measurements of $\overline{A}_B$ and $\overline{\delta}_B$, uncertainty in the area (σA_B) and isotope measurement ($\sigma\delta_B$) is calculated as:

$$\sigma = stdev / n^{1/2} \qquad \text{(Equation 4)}$$

The uncorrected carbon and nitrogen isotope data from the Bennu (OREX-803001-112) and Murchison meteorite extracts and blanks are shown in Supplementary Table 5 and Supplementary Fig. 4. Four different types of blanks were analyzed: (a) empty tin capsules (n = 2), (b) tin capsules exposed to 75 μL Millipore water and 2 μL 6 M hydrochloric acid (HCl) dried under vacuum at room temperature in a centrivap (n = 10), (c) one 73.5 μL procedural blank water extract, and (d) one 68.9 μL fused silica (FS-120) procedural blank water extract (both c and d water extracts were processed in parallel with the Bennu (OREX-803001-112) and Murchison water extracts and were also treated with 2 μL 6 M HCl and dried at room temperature using a centrivap). 71.7 μL of the Bennu water extract and 71.1 μL of the Murchison water extract, each volume representing ~2.6% of the total extracted sample, were pipetted into individual tin capsules along with 2 μL 6 M HCl and then dried under vacuum at room temperature for 2 hours in a centrivap.

Nano EA-IRMS isotope data were corrected for blank contribution by mass balance (Equation 2) using the statistical means of the peak areas and isotope values for tin capsules treated with Millipore water and HCl (capsules + water + acid). The carbon and nitrogen peak areas and isotope values varied among the 10 blanks (Supplementary Table 5). Blanks with peak areas larger than the measured sample overestimate possible background contributions to the sample. Therefore, only the blank analyses with peak areas smaller than the samples were used for the blank correction (Supplementary Table 5 and Supplementary Fig. 4). The uncertainty in blank-corrected isotope values was calculated using Equation 3, where the uncertainty in the blank area and isotope measurements were calculated using Equation 4. For carbon, A_B = 27.8, δ_B = –26.2, σA_B = 1.5, and $\sigma\delta_B$ = 0.2 for OREX-803001-112, and A_B = 30.4, δ_B = –26.2, σA_B = 2.0, and $\sigma\delta_B$ = 0.1 for CM2 Murchison. For nitrogen, A_B = 1.5, δ_B = 3.6, σA_B = 0.2, and $\sigma\delta_B$ = 1.1 for OREX-803001-112, and A_B = 0.8, δ_B = 6.8, σA_B = 0.1, and $\sigma\delta_B$ = 1.8 for CM2 Murchison for nitrogen.

US Geological Survey (USGS, Reston, VA, USA) reference standards 63 (caffeine) and 40 (L-glutamic acid), Urea #1 (Indiana University, Bloomington, IN, USA), and PSU in-house standard Peru mud were used as calibration standards to evaluate the carbon data. Measured $\delta^{13}C$ values were normalized to the VPDB scale with a two-point calibration using USGS 40 and Urea #1. International Atomic Energy Agency (IAEA, Vienna, Austria) standards N-2 and 305B (ammonium sulfate), USGS 40, 63, and 25 (ammonium sulfate), and Urea #1 were used to evaluate the nitrogen data, with IAEA-305B and USGS 25 serving as calibration standards. The detection limit of the nano EA-IRMS system is approximately 30 nmol of nitrogen (~2Vs) and 22 nmol of carbon (~34Vs). CM2 Murchison (UIC) nitrogen was below detection limit (<2Vs).

Nitrogen abundances and isotopes. The abundance of free ammonia (13,613 nmol g^{-1}) in the hot-water extract of Bennu (OREX-803001-0) was more than 12× higher than the ammonia abundance measured in the Murchison hot-water extract (Fig. 1, Extended Data Table 2). The elevated ammonia abundance in the Bennu extract is similar to the free ammonia abundances reported for Orgueil[16,21,22] and a bulk sample of the CR3 meteorite GRA 95229[16]. An elevated amount of ammonia in the Bennu extract was also inferred from the nitrogen loss from the sample following water extraction (100°C for 24 h) calculated from the difference in measured quantities of total nitrogen in the Bennu aggregate before and after hot-water extraction (i.e., before extraction: OREX-803007-0,

0.25 ± 0.01 wt.% N; after extraction: OREX-803001-103, 0.20 ± 0.01 wt.% N, Extended Data Table 1).

The predicted and measured amounts of nitrogen recovered from the dried Bennu water extract (OREX-803001-112) were similar. The mass balance estimate (24 ± 5 nmol N) and the measured amount (6 ± 35 nmol N) were indistinguishable given the range of analytical uncertainties (Supplementary Table 4). Similarly, the calculated estimate of the nitrogen isotope abundance of the water extract ($\delta^{15}N_{AIR}$ = +178 ± 86‰) was similar to the measured value ($\delta^{15}N_{AIR}$ = +180 ± 47‰), within uncertainties (Supplementary Table 4). The Bennu water extract is significantly ^{15}N enriched relative to the hydrazine used for the propulsion system on the OSIRIS-REx spacecraft ($\delta^{15}N_{AIR}$ = 4.7 ± 1.5‰)[75]. Additional information about ammonia as a byproduct of the hydrazine thrusters used on the OSIRIS-REx spacecraft and the potential for sample contamination is discussed later.

Our measured nitrogen isotope value for Bennu (OREX-803001-112) is consistent with pristine meteorites and an outer solar system origin. Ammonia in CR2 (GRA 95229), CM2 (Murchison, Murray), CI1 (Orgueil, Ivuna), and ungrouped C2 chondrites (Tagish Lake, Bells) meteorites all exhibit ^{15}N enrichment over a notable range in ammonia released after hydrothermal treatment of the IOM in the meteorite samples ($\delta^{15}N_{NH4,AIR}$ = +50 to +455‰)[16,65]. The range in $\delta^{15}N$ values for carbonaceous meteorites likely indicates heterogeneity in process associated with the earliest history of the solar system. In contrast, the nitrogen isotope signature of hydrogen cyanide from the CM2 Murchison is more typical of Earth-like values ($\delta^{15}N_{HCN,AIR}$ = +1 to + 11‰)[16,76], indicating HCN had a source distinct from ammonia associated planetary processes[76]. For Bennu (OREX-800031-112), the elevated ^{15}N signature is in the range of carbonaceous meteorites, indicates an outer solar system source, and is inconsistent with a planetary (or spacecraft) source. The elevated amount of ammonia in samples returned from Bennu is important because of its chemical reactivity in prebiotic chemistry and potential for delivery of a reactive source of nitrogen by carbonaceous asteroids to the early Earth[15].

Carbon abundances and isotopes. The measured amount of carbon in the dried extraction water (OREX-803001-112) was significantly lower than predicted by mass balance calculations (Supplementary Table 4). The mass balance estimate for carbon in the water (166 ± 155 nmol C) is nearly an order of magnitude more than was measured in the aliquot (25 ± 53 nmol C). Similarly, carbon isotope abundances did not match between the isotope mass balance estimate ($\delta^{13}C_{VPDB}$ = +80 ± 77‰) and the measured value ($\delta^{13}C_{VPDB}$ = –9 ± 3‰). The low abundance of carbon likely reflects the evolution and loss of CO_2 when the water aliquot was acidified with HCl in order to retain ammonia as ammonium chloride salt as it dried. By mass balance, we estimate the carbon lost (141 nmol C) was ^{13}C-enriched ($\delta^{13}C_{VPDB}$ = +96‰).

The elevated isotopic signature inferred for the lost carbon is similar to carbonate carbon isotope data from the carbonaceous meteorite, Tagish Lake[77], and the range of elevated values observed for CM carbonaceous chondrites[66,78]. The ^{13}C-depleted signature measured in the water extract most likely reflects soluble forms of organic carbon. The value is consistent with average NanoSIMS measurements of carbon in Bennu aggregate insoluble organic matter ($\delta^{13}C$ ~ –11‰). We note the amount of carbon lost as CO_2 indicates that most of the difference in wt.% C observed between OREX-803007-0 and OREX-803001-103 (i.e., before and after hot-water extraction) can be

attributed to carbonate dissolution. This implies just slightly less than 0.3 wt.% C, about 7% of the carbon, measured by EA-IRMS in Bennu OREX-803007-0, was originally present in water-soluble carbonate phases.

Isotopic composition of the hydrazine used in the OSIRIS-REx spacecraft thrusters and ammonia as a byproduct

The OSIRIS-REx spacecraft uses high-purity hydrazine monopropellant thrusters. At sample collection the spacecraft performed a 25.7 s burn with four 4.5 N thrusters[79] canted away from the TAG site to depart the surface of Bennu. OSIRIS-REx was required to impinge less than 180 ng/cm^2 of hydrazine on the TAGSAM surface; a calculation was made under worst case with 0.05% residual hydrazine and an 80.25 s back-away burn[75]. Since monopropellant exhaust is approximately 25% N_2, 25% H_2, and 50% NH_3, the ammonia abundance should be about 1,000 times the molar value for hydrazine from previous plume modeling. Then adjusting for the shorter duration yields 2 µmol/cm^2 (33 µg/cm^2) NH_3 impinging on TAGSAM. This value could be higher or lower depending on the complex dynamics of the plume with regolith lofted by the TAG and back-away[10]. The TAGSAM head was observed to have externally adhering particles, covering a small fraction of the TAGSAM head exterior[10]. It is possible that these particles could have adsorbed 2 µmol/cm^2 NH_3. TAGSAM 6061 aluminum and 304L stainless steel is expected to have lost any NH_3 adsorbed on the metal. Ammonia on 304L stainless steel in vacuum[80] only has a 4% sticking coefficient at 0°C. If the aluminum and stainless steel behave similarly, there would be virtually no NH_3 remaining on TAGSAM metal surfaces when the sample was stowed 8 days after sampling. Thus, any residual NH_3 would not have an opportunity to react with the bulk sample. The N- and H-isotope compositions of the hydrazine as loaded into the OSIRIS-REx spacecraft propellant tanks was measured to be $\delta^{15}N_{AIR}$ = +4.7 ± 1.5‰ and δD_{VSMOW} = +154 ± 23‰[75]. The NH_3 produced by the thruster at high temperature should have similar isotopic values. Future analyses should investigate if the NH_3 in the bulk sample as well as any NH_3-mediated reactions retain the isotopic signature of this monopropellant. We did not detect any hydrazine in the Bennu aggregate water extract OREX-803001-0 using LC-FD/MS (Supplementary Fig. 5, Supplementary Table 6) above the 0.1 nmol g^{-1} level (Extended Data Table 2).

LC-FD/QqQ-MS analysis of the AccQ•Tag derivatives of ammonia, hydrazine, aliphatic amines, and protein amino acids

Standards and reagents. All commercially purchased reagents used were acquired from Sigma-Aldrich, Fisher Scientific, Acros Organics, Combi-Blocks, Bachem, Tokyo Chemical Industry, and Waters Corporation. Amino Acid Hydrolysate H from Waters was utilized along with stock amino acids prepared by dissolving individual analyte crystals (purities ranged from 96 to 100%) in ultrapure water. Stock amino acid standard solutions were made with concentrations ranging from 0.01 to 1 M. Once the individual standard solutions of each species were made, they were combined to facilitate the analysis of all target analytes in a single run. The stock solution for the amines was made to be between 0.01 and 2 M. This solution was then diluted to make 9 standards to facilitate the analysis of all target analytes in a single run.

Sample extraction and AccQ·Tag derivatization method. The OREX-800031-0, Murchison, FS-120, and procedural blank samples were flame-sealed in glass ampoules in 1 mL of Milli-Q ultrapure water and then heated at 100°C for 24 h. The samples were centrifuged, and the water supernatants removed and transferred to separate vials with the following total volumes based on mass: 2,776.5 µL for OREX-800031-0, 2,764.7 µL for Murchison, 2,773.6 µL for the FS-120 fused silica, and 2,950.0 µL for the procedural blank. Approximately 0.35% of the water supernatants was then removed with volumes corresponding to 10.4 µL of the procedural blank, 9.8 µL of OREX-803001-0, 9.8 µL of Murchison, and 9.8 µL of the FS-120 sample. Then sodium borate was added directly to the water extracts to bring the total amount up to 80 µL, 20 µL of the AccQ·Tag derivatization agent was added, and the sample was heated to 55°C for 10 minutes as previously described[81].

As previously reported[32], laboratory experiments designed to study the impact of the 100°C for 24 h water extraction procedure on pure amino acid mixtures have shown that most amino acids do not thermally decompose or racemize during hot water extraction. However, unsurprisingly we have observed in testing with standards that some more fragile protein amino acids, such as asparagine, are not stable in hot water. Asparagine can undergo deamidation to succinimide followed by hydrolysis to aspartic acid and isoaspartic acid with rates that are dependent on the temperature and pH[54]. Glutamine and cysteine are also unstable in hot water and these protein amino acids could have also decomposed during the hot water extraction procedure as noted in a footnote in Supplementary Table 12. The previous experiments with standards to test for amino acid decomposition during hot water extraction were not done in the presence of an inorganic matrix, therefore we are unable to rule out the possibility that additional modification of the amino acid content in the Bennu aggregate and meteorite samples could have occurred due to the presence of minerals. However, previous studies have been performed with pure aliphatic amine standards mixed with serpentine (a hydrated magnesium silicate used as a meteorite analog) to test the impact of the 100°C for 24 h water extraction procedure on amines and no measurable effects on their molecular distributions or isotopic compositions were reported[82].

LC-FD/QqQ-MS analyses. The AccQ·Tag derivatized free ammonia, amino acids and amines in the water extracts were then analyzed via the commercial Waters AccQ·Tag protocol on a Xevo TQS-Micro triple quadrupole mass spectrometer equipped with an electrospray ionization source (positive ion mode) using multiple reaction monitoring (MRM) mode. The Xevo TQ-S Micro capillary voltage was set to 1.0 keV, the sampling cone was set to 40°C, the source temperature was set to 150°C, the cone gas flow was set to 50 L/h, the desolvation temperature was set to 500°C, and the desolvation gas flow was set to 1000 L/h. Samples were introduced via a Waters Acquity H-Class plus UHPLC with a fluorescence detector.

For the UHPLC analyses of amines and amino acids, a 250 µL syringe, 50 µL loop, and 15 µL needle were used. UHPLC separations were performed using two AccQ·Tag Ultra C18, 1.7 µm. 2.1 × 150 mm columns in series. Amino acid target analytes were eluted using the following gradient: 0–15min: 99.9% eluent A, 15–20 min: 99.9–95% eluent A, 20–22 min: 5% eluent A, 22–29 min: 95–90% eluent A, 29–35 min: 90–78.8% eluent A, 35–38 min: 78.8% eluent A, 38–45 min: 78.8–40.4% eluent A, 45–55 min: 40.4%

eluent A, 55–57 min: 40.4–99.9% eluent A, 57–60 min: 99.9% eluent A. The autosampler temperature was maintained at 25°C, the injection volume was 10 µL, the eluent flow rate was held at a constant 0.15 mL min^{-1}, and the column was maintained at 55°C. Amine target analytes were eluted using the following gradient: 0–2.49 min: 0–10% eluent B, 2.49–7 min: 10–20% eluent B, 7–7.99 min: 20–50% eluent B, 8–8.99 min: 100% eluent B, 8.99–9 min: 100–0% eluent B, 9–10 min: 0% eluent B. The autosampler temperature was maintained at 25°C, the injection volume was 1 µL, the eluent flow rate was held at a constant 0.7 mL min^{-1}, and the column was maintained at 55°C. The fluorescence detector was operated with an excitation wavelength of 266 nm and an emission wavelength of 473 nm. For the UHPLC analysis of ammonia and hydrazine, a 250-µL syringe, 50-µL loop, and 15-µL needle were used. UHPLC separations were performed using one AccQ·Tag Ultra C18, 1.7 µm × 2.1 × 150 mm column. Ammonia and the protein amino acids were eluted using the following gradient: 0–0.54 min: 0.1% eluent B, 0.54–5.74 min: 0.1–10% eluent B, 5.74–7.74 min: 10–21.2% eluent B, 8.04–8.64 min: 59.6% eluent B, 8.64–8.73 min: 59.6–0.1% eluent B, 8.73–10.00 min: 0.1% eluent B.

Typical LC-FD chromatograms of the AccQ·Tag derivatives of the free amino acids and amines in the standards and the hot-water extracts of Bennu (OREX-803001-0), Murchison, and the procedural blank are shown in Supplementary Fig. 6. Similarly, the LC-FD/QqQ-MS chromatograms showing the identification of the AccQ·Tag derivative of ammonia in the Bennu and Murchison water extracts are shown in Supplementary Fig. 7. Selected MRM transitions were used for the abundance quantifications for ammonia, amines, and amino acids in these analyses (Supplementary Tables 7 and 8). A linear least-square model was fitted to ammonia and each protein amino acid and amine in the standard calibration set, and these calibration curves were used to quantify the analytes in the samples. A sample of pure water that was carried through the same preparation and analytical procedures as the meteorites was used as a blank to determine the procedural and laboratory backgrounds. All derivatized extracts were analyzed in triplicate, and the average blank-corrected ammonia, protein amino acid, and amine concentrations of the samples were determined from the standard calibration set and the extracted sample mass.

LC-FD/ToF-MS analysis of the OPA/NAC derivatives of amino acids and their enantiomeric ratios

Standards and reagents. All glassware, ceramics, and sample handling tools used in sample processing were rinsed with Milli-Q ultrapure water (18.2 MΩ·cm, <3 ppb total organic carbon), wrapped in aluminum foil, and then heated in a furnace at 500°C in air overnight. Most of the chemicals and reagents were purchased from Sigma-Aldrich. A stock amino acid solution (1 × 10^{-6} M) was prepared by mixing individual amino acid standards (97–99% purity) in Milli-Q ultrapure water. All chiral amino acid standards were purchased as racemic mixtures (D = L), except for D- and L-threonine (Sigma-Aldrich, >98% purity, *allo*-free) and D- and L-isovaline (Acros Organics, >99% purity) which were prepared as racemic mixtures by mixing the appropriate masses of each compound in Milli-Q ultrapure water to the standard mixture. Acid vapor hydrolysis used Tamapure-AA-10-HCl 20% (metallic impurity level < 10 pg/mL). Cation-exchange resin (AG50W-X8, 100–200 mesh, hydrogen form, BIO-RAD) was used for removal of salts and interfering

ions from samples. During the desalting protocol, 1.5 N HCl, 2 M sodium hydroxide (NaOH), and 2 M ammonium hydroxide (NH_4OH) were used. The 2 M NaOH was produced by dissolution of 32 g of NaOH pellets (Sigma-Aldrich, anhydrous, ≥97%) in 400 mL Milli-Q ultrapure water, and the 2 M NH_4OH was prepared from Milli-Q ultrapure water and ammonia gas (Air Products) in vacuo. Pre-column derivatization of samples prior to LC-FD/ToF-MS analyses involved the use of 0.1 M sodium borate, *o*-phthaldialdehyde/*N*-acetyl-L-cysteine (OPA/NAC), and 0.1 M hydrazine hydrate. Sodium borate was generated by heating solid sodium borate decahydrate at 500°C, in air, for 3 h, prior to dissolution in Milli-Q ultrapure water. The OPA/NAC derivatization reagent was prepared by first generating 0.1 M OPA via dissolving 0.1 g OPA in 7.5 mL methanol (Optima Grade), then generating 0.5 M NAC via dissolving 0.408 g NAC in 5 mL Milli-Q ultrapure water, and then mixing 300 µL of 0.1 M OPA with 30 µL of 0.5 M NAC and 670 µL of 0.1 M sodium borate. The 0.1 M hydrazine (NH_2NH_2) solution was prepared by vacuum distillation of concentrated anhydrous hydrazine (98% purity) and subsequent dilution in Milli-Q ultrapure water.

Extraction and derivatization methods for amino acid analyses. The OREX-800031-0, Murchison, FS-120, and procedural blank (empty glass ampoule) were flame-sealed in pre-scored glass ampoules each containing 1 mL of Milli-Q ultrapure water, and the sealed ampoules were placed in a heating block inside an oven set at 100°C for 24 h. After heating, the glass ampoules were removed from the oven, allowed to cool to room temperature, centrifuged inside polypropylene Falcon tubes at 3,000 rpm for 5 min to separate solid particles from the liquid. The ampoules were opened, and the water supernatants were transferred from the ampoules by pipetting into pre-weighed amber glass vials. Another 1 mL of Milli-Q ultrapure water was added to each glass ampoule, the ampoules were re-centrifuged, and the supernatant was transferred to the sample amber glass vials (this process was repeated one final time to maximize the recovery of the water extracts). The total masses of the combined water supernatants transferred from each sample in the amber vials were determined using a balance with masses as follows: 2,776.5 mg for OREX-800031-0, 2,764.7 mg for Murchison, 2,773.6 mg for the FS-120, and 2,950.0 mg for the procedural blank. After extraction, 40% of the supernatant was dried under vacuum and subsequently subjected to a 6 M HCl vapor hydrolysis at 150°C for 3 h to determine total hydrolysable amino acid content. The sample was divided by volume. Since we were using water as the carrier, and water has a density of 1 g/cc, we used 1,110 µL of OREX-80031-0, 1,106 µL of Murchison, 1,110 µL of FS-120 fused silica, and 1,180 µL of the procedural blank. The HCl acid-hydrolyzed, hot-water extracts were then desalted by using cation-exchange resin (AG50W-X8, 100–200 mesh, hydrogen form, BIO-RAD), and the amino acids recovered by elution with 2 M NH_4OH (prepared from Millipore ultrapure water and NH_3(g) (AirProducts) *in vacuo*). An additional 40% of the remaining non-hydrolyzed water extracts of the samples were dried down under vacuum and taken through the identical desalting procedure in parallel with the acid-hydrolyzed extracts to determine the abundances of the free amino acids. After desalting, the samples were dried under vacuum and brought up in 100 µL of water, and 30 µL were taken and dried down with 20 µL of pH = 9 sodium borate buffer. After drying down, the samples were brought up in 20 µL of water and 5 µL of 0.1 M OPA/NAC

derivatization agent and allowed to react for 15 minutes at room temperature before being quenched with 75 µL of 0.1 M hydrazine.

LC-FD/ToF-MS analyses. Amino acid abundances, distribution, and enantiomeric ratios were determined by LC-FD/ToF-MS. The amino acids in the NH_4OH eluates were derivatized with OPA/NAC for 15 minutes at room temperature followed by their separation and analysis using a Waters ACQUITY UPLC and Waters Xevo G2-XS Q-ToF-MS operating in positive ion mode. C_2 to C_6 amino acids were chromatographically resolved using a Waters BEH C18 column (2.1 × 50 mm, 1.7 µm bead) and a Waters BEH phenyl column (2.1 × 150 mm, 1.7 µm bead) in series. Both columns were maintained at 30.0°C. The mobile phase conditions for amino acid separations were as follows: flow rate, 150 µL/min; gradient, time in minutes (%B): 0 (0), 35 (55), 45 (100). C_5 amino acid isomers and enantiomers were chromatographically separated using the same chromatography conditions as for the C_2 to C_6 amino acids but required the implementation of a different gradient. The gradient used for C_5 amino acid isomers and enantiomers was structured via time in minutes (%B): 0 (15), 25 (20), 25.06 (35), 44.5 (40), 45 (100).

During the Xevo G2-XS analysis, a dual electrospray ionization (ESI) system was used for the purpose of implementing lock mass corrections. The primary ESI source was operated using the following parameters: capillary voltage, 3.0 kV; sampling cone voltage, 40 V; source temperature, 120°C; desolvation gas (N_2) temperature, 350°C; cone gas (N_2) flow, 50 L h^{-1}, desolvation gas flow rate, 750 L h^{-1}. Due to the possibility that minor variations in the mass-to-charge (m/z) scale may occur during the course of executing experimental runs after instrument calibration is performed, a reference ESI source was implemented to supply an independent leucine enkephalin standard signal. The reference ESI source was operated using a sample infusion rate of 20 µL min^{-1}, a sample fill volume of 250 µL, a lockspray infusion rate of 10 µL min^{-1}, a capillary voltage of 3.0 kV, a reference cone voltage of 30 V, and a collision energy of 6.0 V. The ToF analyzer was operated in "Sensitivity mode," which used a reflectron to provide a full width at half maximum resolution of <22,000 based on the $[M+H]^+$ of leucine enkephalin, m/z 556.2771.

The amino acid abundances and their enantiomeric ratios in the meteorite extracts and controls were determined by comparison of the peak areas generated from the sample and control UV fluorescence chromatograms (LC-FD, λ_{ex} = 340 nm, λ_{em} = 450 nm) of their OPA/NAC derivatives to the corresponding peak areas of amino acid standards run under the same chromatographic conditions and included peak identification confirmation by accurate mass using a match tolerance of 10 ppm (ToF-MS, see Supplementary Tables 9 and 10). Typical LC-FD chromatograms of the OPA/NAC derivatives of amino acids in the Bennu (OREX-803001-0), Murchison, and procedural blank acid-hydrolyzed, hot-water extracts is shown in Supplementary Fig. 8. The LC-ToF-MS chromatograms showing separation of the C_5 amino acids in the same extracts is also shown in Supplementary Fig. 9.

LC-FD/HRMS analysis of the OPA/NAC derivatives of amino acids and their enantiomeric ratios

Standards and reagents. Chemical reagents used for these analyses were either procured from Mann Research Laboratories, Sigma-Aldrich, Fisher Chemical, Acros Organics, or Honeywell Research Chemicals. Amino acid crystals that were used to produce individual stock analytical standards had purities ≥96.8%. All other chemicals used for these analyses had purities of ≥95%, unless otherwise stated. Individual amino acid standard solutions were made at concentrations of 10^{-3} M to 10^{-1} M by separately dissolving crystals from each amino acid into Milli-Q ultrapure water. These individual amino acid standards were then combined to create a mixed amino acid standard that facilitated the analysis of all targeted amino acids in a single run. All chiral amino acids included in this mixed standard were prepared as racemic mixtures.

Preparation of OPA/NAC derivatization reagents was conducted as detailed in the LC-FD/ToF-MS analytical section, with the exception that the 0.1 M sodium borate used during derivatization was prepared using sodium tetraborate, as opposed to sodium borate decahydrate. The sodium tetraborate used during the analyses described here was first baked out overnight at 500°C, in air. Next, 2.03 g of baked-out sodium tetraborate was dissolved in 100 mL of Milli-Q ultrapure water to reach a final concentration of 0.1 M.

Liquid chromatography analyses of C_2–C_{11} amino acids relied on the use of two mobile phases: A) 35 mM ammonium formate with 7% methanol, pH adjusted to 9.0 and B) LC-MS grade methanol. Mobile phase A) was prepared by combining 780 mL of LC-MS grade water with 1.51 mL of LC-MS grade formic acid, followed by titrating this solution to pH 9.0 using 2 M aqueous ammonium hydroxide, and lastly adding 64 mL of LC-MS grade methanol. The 2 M aqueous ammonium hydroxide solution was prepared by diluting a 7.5 M stock solution of aqueous ammonium hydroxide (assay = 29.3%, ammonia in water) with LC-MS grade water to obtain a 2 M concentration. Mass calibrations of the high-resolution mass spectrometer were performed using the Thermo Scientific Pierce LTQ Velos ESI positive ion calibration mix. This calibration mix was an aqueous solution that included methanol, acetic acid, and acetonitrile. The calibration analytes in this mix were Ultramark 1621, MRFA (Met-Arg-Phe-Ala), and caffeine.

Sample preparation. Samples, blanks, and standards were derivatized as detailed in the LC-FD/ToF-MS analytical section, with the exception that standards were derivatized by first drying down 10 µL aliquots of the standard with 20 µL aliquots of 0.1 M sodium borate, as opposed to samples and blanks in which 30 µL aliquots of each blank and sample were dried down with separate 20 µL aliquots of 0.1 M sodium borate. Prior to analysis, blanks, samples, and standards were each derivatized once. Following analysis, all derivatization vials were stored at –80°C and reused for subsequent injections to perform replicate analyses. This cold storage approach was used to mitigate derivative degradation between replicate injections.

LC-FD/HRMS analyses. Amino acids were analyzed using a Thermo Fisher Scientific Vanquish Horizon liquid chromatograph coupled to a Thermo Fischer Scientific Vanquish fluorescence detector, and a Thermo Fisher Scientific Q Exactive hybrid quadrupole-Orbitrap mass spectrometer. Amino acid identifications were made by the observation of the following three measurable properties in comparison to a mixed amino acid standard: 1) chromatographic retention time, 2) optical fluorescence, and 3) accurate mass. The analyses implemented a mass tolerance of 3 ppm (Supplementary Tables 9 and 10). The

LC-HRMS chromatograms showing the elution of the C_2 to C_{11} amino acids in the acid hydrolyzed, hot-water extracts of the procedural blank, Bennu (OREX-803001-0), and the CM2 Murchison meteorite are shown in Extended Data Fig. 2. After LC-HRMS identification, amino acid quantitation was executed via manual integration of analyte peak areas using the Thermo FreeStyle software program.

Chromatographic separation was achieved using a 2.1 × 5 mm, 1.7-μm particle size Waters ACQUITY UPLC Peptide BEH C18 VanGuard Pre-column, followed by the following three stationary phases in series: 1) 2.1 × 150 mm, 1.7-μm particle size Waters ACQUITY UPLC CSH Phenyl-Hexyl, 2) 2.1 × 150 mm, 1.7-μm particle size Waters ACQUITY UPLC CSH C18, and 3) 2.1 × 150 mm, 1.7-μm particle size Waters ACQUITY UPLC CSH Phenyl-Hexyl. The C_2–C_8 amino acids were eluted using the following gradient: 0–60 min, 0–33% eluent B, 60–70 min, isocratic at 33% eluent B, 70–75 min, 33–45% eluent B, 75–80 min, isocratic at 45% eluent B, 80–100 min, 45–83% eluent B, 100–100.1 min, 83–100% eluent B, 100.1–105 min, isocratic at 100% eluent B, 105–105.1 min, 100–0% eluent B, 105.1–120 min, isocratic at 0% eluent B. The eluent flow rate was 0.11 mL min^{-1}. The stationary phases were maintained at 34°C. A pre-column heater was used, which was also kept at 34°C. The injection volume was 10 μL, and the autosampler was held at a temperature of 5°C. The fluorescence detector utilized an excitation wavelength of 340 nm and an emission wavelength of 450 nm. The fluorescence detector was kept at a constant temperature of 34°C.

The HRMS system was configured with a heated electrospray ionization (HESI) source and was operated using the following parameters: spray voltage = 3.50 kV, sheath gas (N_2) flow rate = 36 arb. unit, auxiliary gas (N_2) flow rate = 10 arb. unit, sweep gas (N_2) flow rate = 1 arb. unit, capillary temperature = 250°C, auxiliary gas heater temperature = 220°C, and S-lens RF level = 50.0%. The HRMS system was operated in Full MS–SIM scan mode according to the following parameters: polarity = positive, scan range = 100–1,500 *m/z*, mass resolution setting = 70,000 (at full-width-half-maximum for *m/z* 200), automatic gain control target = 1×10^6 ions, and maximum injection time = 200 ms. The HRMS system was calibrated daily over the 50–2,000 *m/z* range, which facilitated a mass accuracy of <2 ppm.

GC-QqQ-MS analysis of carboxylic acids

The portions of the hot-water extract allocated for analysis of carboxylic acids were basified with 20 μL of 2 M NaOH, dried under vacuum, and then derivatized with 2-pentanol using previously described methods[26,83]. The dry residues were suspended in 20 μL of 6 M HCl, 30 μL of 2-pentanol, 200 μL of DCM, and heated at 100°C for 16 h in sealed PFTE-lined screw cap vials in a heating block. After cooling to room temperature, the derivatized samples were passed through a short plug of aminopropyl silica gel (25 mm length × 5 mm I.D.), rinsed using ~3 mL of dichloromethane (DCM), dried with flowing N_2, and dissolved in 150 μL of DCM for analysis. We quantified the concentrations of carboxylic acids in the samples and procedural blank by gas chromatography coupled to triple-quadrupole mass spectrometry detection (GC-QqQ-MS). The abundances of carboxylic acids were quantified from the peak areas generated using the average value of three separate GC-QqQ-MS measurements on the same sample.

The derivatized carboxylic acids were analyzed using a Thermo Trace 13100 GC equipped with a 5 m base-deactivated fused silica guard column (Restek, 0.25 mm I.D.), two Rxi-5ms (30 m length × 0.25 mm I.D. × 0.5 µm film thickness; capillary columns connected in series using SilTite µ-union connectors, Restek), and coupled to a Thermo TSQ electron-impact triple-quadrupole mass spectrometer (ion source set at 220°C and 70 eV). The oven program used started with the temperature held at 40°C for 1 min, then ramped at 15°C min^{-1} to 110°C, ramped at 10°C min^{-1} to 140°C and held for 2 min, ramped at 10°C min^{-1} to 145°C, and finally ramped at 30°C min^{-1} to 300°C with a final hold time of 5 min. The carrier gas used was ultrahigh purity grade helium (5.0 grade) at 4.2 mL min^{-1} for carboxylic acids. Triplicate injections of derivatives were made in split mode (split flow: 5 mL min^{-1}, held for 1 min) in aliquots of 1 µL. The GC-QqQ-MS mass chromatograms of carboxylic acids identified in the Bennu and Murchison hot-water extracts and in the procedural blank and standards is shown in Supplementary Fig. 10. The mass spectra were used to identify and quantify the carboxylic acid derivatives by comparison to reference standards and application of calibration curves as described elsewhere[83].

As previously noted for amino acids, we cannot exclude the possibility that some carboxylic acids could have degraded during the hot water extraction procedure as has been shown for malonic acid[20,28]. However, previous studies have been performed with pure standards mixed with serpentine (a hydrated magnesium silicate used as a meteorite or asteroid analog) to test the impact of the 100°C 24 h water extraction procedure on monocarboxylic acids and no measurable effects on their molecular distributions or isotopic compositions were reported[26].

HPLC-HRMS analysis of N-heterocycles

Standards and reagents. Authentic standards for the nucleobases and other N-heterocyclic compounds were purchased from Tokyo Chemical Industry, Sigma-Aldrich, FUJIFILM Wako Pure Chemical, Combi-Blocks, Toronto Research Chemicals, and BLD Pharmatech Ltd. Stock standard solutions of N-heterocyclic compounds were prepared by dissolving individual analyte crystals (purities ranged from 96 to 100%) in MilliQ-water (Millipore Milli-Q grade, 18.2 MΩ·cm). Ultrapure water and 6 M hydrochloric acid (HCl) (Tama Chemicals Co., Ltd., Japan; Tama pure AA-10 grade), 3 M NaOH solution (Kanto Chemical Industry Co., Ltd., Japan; ultrapur™ grade), and ammonia solution (Kanto Chemical Industry Co., Ltd., Japan; ultrapur™ grade, 28.0%~30.0% in water) were obtained for the extraction and purification procedures. Solutions of 1 M and 0.1 M HCl, 1 M NaOH, and 10% ammonia in water (NH_4OH) were prepared and diluted from the above-mentioned solvents using ultrapure water. Ultrapure water, acetonitrile (ToF-MS grade), and formic acid (LC-MS grade; >99.5% purity) were sourced from FUJIFILM Wako Pure Chemical for HPLC/ESI-HRMS analyses. All the glassware and sea sand (FUJIFILM Wako Pure Chemical Corporation, Japan; 30–50 mesh) used for procedural blanks were rinsed with MilliQ-water, wrapped in aluminum foil, and subsequently heated at 450°C for 5 h in air prior to use.

Extraction and purification of N-heterocycles. 17.75 mg of fine- to intermediate-sized particles of the Bennu sample OREX-800044-101 were soaked in 300 µL of 6 M

HCl in a glass vial. After purging with dry N_2 gas to remove O_2 in the headspace, the glass vial was flame-sealed and heated at 110°C for 12 h. After heating, the supernatant and the Bennu sample particles were transferred to a 1.5 mL polytetrafluoroethylene (PTFE, Teflon) vial followed by centrifugation for 1 min at 10,000 rpm. The supernatant was transferred to the sample extract vial. The residue was washed twice with 300 μL of ultrapure water, and the rinse was mixed with the supernatant. The mixed supernatant was freeze-dried under reduced pressure.

The dried extract was dissolved in 0.5 mL of 0.1 M HCl for performing a desalting procedure using an improved method of cation-exchange chromatography[62,84]. In brief, 0.5 mL of AG 50W-X8 cation-exchange resin (Bio-Rad Laboratories, Inc.; analytical grade, 200–400 mesh, hydrogen form) was placed in a Pasteur glass pipet and rinsed with solvents in the following order: 1.5 mL of 1 M HCl, ultrapure water, 1 M NaOH, ultrapure water, 1 M HCl, and ultrapure water. The extract was loaded onto the cation-exchange chromatography column. The cation-exchange resins were washed with 2.5 mL of ultrapure water to recover acidic, neutral, and weakly basic compounds referred to as "H_2O fraction". Subsequently, 2.5 mL of 10% NH_4OH was loaded onto the H_2O-washed cation-exchange resins to elute basic compounds, including most nucleobases referred to as "NH_4OH fraction". The H_2O and NH_4OH fractions were freeze-dried and reconstituted into 50 μL of ultrapure water. Simultaneously, we prepared a procedural blank with baked sea sand powder using the same protocol as that applied to the Bennu samples; we analyzed the blank to validate the background signal during the procedures.

HPLC/ESI-HRMS analyses. The H_2O and NH_4OH fractions from the Bennu sample and the procedural blank, and the authentic standards of the targeted molecules were analyzed using an online HPLC/ESI-HRMS system comprising an UltiMate 3000 and Q Exactive™ Plus Hybrid Quadrupole-Orbitrap™ mass spectrometer (Thermo Fischer Scientific Inc., Waltham, MA, USA) with a mass resolution of 140,000 at a mass-to-charge ratio m/z = 200[36,62,85]. The HPLC instrument was outfitted with a reversed-phase separation column maintained at 40°C. For the detection and quantification of most purine nucleobases, we employed the following isocratic HPLC eluent program with an InertSustain PFP column (1.0 mm × 250 mm, particle size = 3 μm, GL Sciences Inc., Tokyo, Japan): solvent A (water) and solvent B (acetonitrile with 0.1% formic acid) = 90:10, held for 20 min with a flow rate of 0.05 mL min^{-1}. For pyrimidine nucleobase analyses, we used the following gradient HPLC eluent program with the HyperCarb™ column (2.1 mm × 150 mm, particle size = 3 μm, Thermo Fischer Scientific Inc., Waltham, MA, USA): solvent A (water + 0.1% formic acid) and solvent B (acetonitrile + 0.1% formic acid) = 99:1 at t = 0 min, followed by a linear gradient of A:B = 70:30 at 20 min with a flow rate of 0.2 mL min^{-1}.

We then introduced the compound solution separated using either the PFP or HyperCarb™ column into a HESI-II probe (Thermo Fischer Scientific Inc., Waltham, MA, USA) and heated it at 280°C for desolvation. The spray voltage and capillary temperature of the ion-transfer system were 3.5 kV and 295°C, respectively. To detect various organic molecules in the hot-water (HW) and HCl extracts, we recorded the mass spectra of the vaporized compounds in the positive ions over an m/z range of 111–155 or 50–500 (Supplementary Figs, 11–13), with the mass determined to an accuracy better than 5 ppm, as defined by [(measured m/z) – (calculated m/z)]/(calculated m/z) × 10^6 (ppm). The

mass accuracy was occasionally calibrated using the exact masses of protonated tyrosine (m/z = 182.0812), *tert*-butylamine (m/z = 74.0964), and a fragment ion of *tert*-butylamine (m/z = 57.0699). A positive ion with m/z = 83.0604, corresponding to an acetonitrile dimer, was used as the lock mass. For robust identification and quantification of the nucleobases, we performed the tandem mass spectrometry (MS/MS) experiments using the same ionization conditions as those used for the full-scan analyses. We subjected the targeted positive ions isolated by the quadrupole (using an isolation window of 0.4 m/z) to high-energy collisions with N_2 gas to produce fragmented ions and monitored specific mass ranges using an Orbitrap MS with a mass resolution of 140,000 at m/z = 200. Furthermore, we identified guanine in the Bennu extract based on their chromatographic retention times, exact masses, and mass-fragmentation patterns in the MS/MS measurements (Supplementary Fig. 13).

Wet-chemistry pyrolysis GC-QqQ-MS analyses of amino acids and N-heterocycles

Sample preparation and reagents. All glassware used to handle the samples and the pyrolysis tubes themselves were previously ashed at 550°C for ~16 h in air. Prior to pyrolysis, two ~1 mg Bennu aggregate samples (OREX-501029-0 and OREX-803004-0) and equivalent sample masses of Murchison used for the wet-chemistry pyrolysis experiments were prepared inside a chemical fume hood by adding 5 μL *N*-(*tert*-butyldimethylsilyl)-*N*-methyltrifluoroacetamide (MTBSTFA):*N,N*-dimethylformamide (4:1 v/v) solution (MTBSFTA from Sigma Aldrich, >97% purity; DMF from Sigma Aldrich, anhydrous, 99.8% purity) to the sample inside the pyrolysis tubes. The pyrolysis tubes containing sample and reagent were then placed inside a secondary 2 mL vial and capped with special attention to create a seal between the pyrolysis tube and polytetrafluoroethylene (PTFE) liner. Samples were then placed inside a stainless-steel heating block at 85°C for 1.5 h and vortexed every 15 min. Once complete, samples were passively cooled at room temperature for 10 min and immediately transferred to the pyroprobe chamber for pyrolysis.

Pyrolysis gas chromatography–triple quadrupole mass spectrometry (PyGC-QqQ-MS). Experiments were conducted using a CDS Analytical 6200 pyroprobe configured for manual loading with flash (10°C ms^{-1}) pyrolysis ramps of the solid samples with the MTBSTFA and DMF reagents. These derivatized samples were heated rapidly from 50 to 250°C to volatilize and thermally desorb derivatized (silylated) amino acids and N-heterocycles. The pyroprobe housing and valves were held at 300°C, and volatiles were transferred via a heated transfer line (300°C) directly into a Thermo Scientific TRACE 1600 gas chromatograph (GC) coupled to an Thermo Scientific 9610 triple quadrupole mass spectrometer (TSQ) system. The inlet temperature was held at 300°C and operated with a 2:1 split for wet-chemistry pyrolysis. The GC was fitted with an Rtx-5MS fused silica capillary column (30 m × 0.25 mm × 0.25 μm), He carrier flow at 1.5 mL min^{-1}, and MS transfer line set to 300°C. The GC oven was programmed with the following method: 40°C hold for 5 min, followed by a 3.5°C min^{-1} ramp to 300°C, then a final isothermal hold at 300°C for 8.5 min (~88 min total).

The MS source was held at 300°C and was operated in electron impact (EI) mode at 70 eV in simultaneous multiple reaction monitoring (MRM) and full scan in the m/z 50–

550 range. The wet-chemistry pyrolysis GC-QqQ-MS runs included MRM transitions targeting silylated protein amino acids and N-heterocycles previously identified in meteorites and interstellar ice analogs as determined by the pyrolysis of standards. Pyrolysis blanks preceded all standard pyrolysis experiments to control the cleanliness of the analytical set-up and prevent potential cross-contamination. Pyrolysis of reagent blanks (only 5 µL MTBSTFA:DMF) preceded wet-chemistry pyrolysis experiments to characterize persisting background contamination of standards. Results were analyzed using Chromeleon 7.3.1 software. Compound identification was conducted via comparison with retention time and three MRM transitions of standards (Supplementary Table 11). The total-ion chromatograms (full scan & multiple reaction monitoring, MRM) showing the detection of amino acids and N-heterocycles after wet-chemistry pyrolysis GC-QqQ-MS analyses of OREX-501029-0, OREX-803004-0, the Murchison meteorite, and the standards are shown in Supplementary Fig. 14. Comparisons of the protein amino acids and N-heterocycles detected by GC-QqQ-MS after wet chemistry and pyrolysis compared to those identified in the hot water and HCl extracts by LC-MS are shown in Supplementary Tables 12 and 13, respectively.

Nontargeted molecular profiling of soluble organic matter using FTICR-MS

Sample preparation. The 3.3 mg sample used for FTICR-MS analysis was OREX-803006-0. A subsample from OREX 803006-0 (OREX-803141-0) was analyzed with SEM-EDS and laser Raman spectroscopy. For FTICR-MS, we gently washed the sample rapidly with methanol and crushed the grains in a mortar with 400 µL methanol. The slurry was sonicated for 30 s and centrifuged. The supernatant was used for direct injection analysis.

FTICR-MS. FTICR/MS equipped with a 12-Tesla superconducting magnet in negative and positive mode ESI(–), ESI(+), and positive atmospheric pressure photoionization (APPI(+)) in direct sample injection was used at the Helmholtz Munich. The same conditions were used for the Ryugu sample to enable direct comparison, and a detailed description of the analysis and data evaluation was described earlier[86]. The FTICR-MS mass spectrum at nominal mass m/z 319 with annotated mass signals of the Bennu (OREX-803006-0) and Murchison (CM2) methanol extracts compared to similar analyses of a methanol extract of Ryugu (A0106) is shown in Supplementary Fig. 15.

Optical microscopy and micro two-step laser mass spectrometry (µ-L^{2}MS) imaging

Sample preparation. A subsample of the QL aggregate (OREX-501006-0) was prepared, under a laminar flow bench, by dispersing approximately a dozen grains (~ 100 µm diameter) onto a 1-inch diameter potassium bromide (KBr) window. These particles were then gently compacted into the KBr surface using an optically flat sapphire window. No further processing of the sample was required for any of the subsequent measurements.

Optical and UV fluorescence imaging. Optical and UV fluorescence imaging were performed using an Olympus BX-60 microscope equipped with a BX-FLA reflected light

fluorescence source (high-pressure 100W Hg-arc lamp). High resolution (*i.e.*, pixel resolution < Abbe diffraction limit) through focus image stacks of individual grains were acquired using a 50×/0.80 or 100×/0.95 UMPanF objective in combination with a 5.9 megapixel (2880 × 2048 pixel) Nikon DS-Fi3 CMOS image sensor and Nikon NIS Elements software. Image stacks were subsequently post-processed[87] to render composite extended depth-*of*-field images. For native fluorescence imaging acquisition times were typically 300 ms using a 330–385 nm excitation, 420 nm long-pass emission filter cube.

μ-L²MS analyses. The general operation of two-step laser mass spectrometry and its application to analysis of aromatic moieties in astromaterials has been previously described[88-92]. In the first step, laser desorption is used to release neutral organic molecules from the surface of the sample into vacuum while in the second step, a separate laser is used to photoionize the desorbed organics which are injected into a reflectron time-of-flight mass spectrometer. For the analyses performed herein two modifications to the basic instrument setup were employed: (1) a pulse shaping plasma shutter was used[93] to clip the duration of the infra-red (IR) CO_2 laser (Laser Science Inc., PRF-150) desorption pulse to ~ 100 ns and improve the spatial beam profile to ensure a 5 μm analysis spot size when focused onto the sample using a Cassegrain microscope objective; and, (2) a coherent vacuum ultraviolet (VUV) radiation source was used for non-resonant single photon ionization (SPI). This was achieved by the non-linear frequency tripling of the 3rd harmonic (λ 355 nm) of a mode-locked Q-switched picosecond Nd:YAG laser (EKSPLA PL2250) in a Xe-Ar gas cell (Xe:Ar 1:10; 80 Torr) to produce 118.2 nm (λ 10.5 eV) radiation[94,95]. Since the first ionization potentials for nearly all organic molecules lie in the range of 5–10 eV[96], single photon ionization with VUV radiation is capable of soft ionization of virtually all organic compound classes[94,95,97-100].

Prior to analysis of the Bennu aggregate, a reference / calibration sample was used to establish a consistent, comparable set of operating conditions. This was composed of finely powdered and homogenized Allende (CV3) matrix that was pressed in Au foil that provides a congruous reproducible well documented spectrum. The VUV photoionization step was first optimized by gas-phase ionization of a 1:1:1 mixture of acetone (CH_3COCH_3), cyclohexane (C_6H_{12}) and toluene ($C_6H_5CH_3$) introduced into the vacuum chamber via a manual SS sapphire-sealed variable leak valve. After which the IR laser desorption was maximized, subject to no concomitant ionization, through control of the cavity discharge voltage in combination with a wire grid polarizer / attenuator. These conditions were subsequently maintained through continuous monitoring of laser powers and shot-to-shot stability.

For the Bennu aggregate analysis the KBr mounted sample was attached to a 1-inch stainless steel (ss) sample platter using two thin strips of vacuum compatible adhesive tape (PELCO Tabs™ Carbon Conductive Tabs); the sample platter was previously cleaned by ultrasonication in isopropanol and acetone, and then vacuum dried. After loading the sample platter into the μ-L²MS main vacuum chamber, it was allowed to degas during which time the gas phase background was periodically monitored by taking μ-L²MS spectra with the infrared desorption laser blocked. Direct sample analysis began only after the vacuum chamber pressure had returned to its normal operating range ($<10^{-7}$ Torr; 1.3 $\times10^{-5}$ Pa) and there was no gas phase background interference. Spatial mapping of a

sample was then performed by rastering the sample platter under the focus of the IR desorption laser in 5 µm steps and acquiring mass spectra at each location. The signal intensity of a given molecular species is a product of its photoionization cross-section and abundance.

References

65. Foustoukos, D. I., Alexander, C. M. O'D. & Cody, G. D. H and N systematics in thermally altered chondritic insoluble organic matter: An experimental study. *Geochim. Cosmochim. Acta* **300**, 44–64 (2021).

66. Vacher, L. G., Marrocchi, Y., Verdier-Paoletti, M. J., Villeneuve, J. & Gounelle, M. Inward radial mixing of interstellar water ices in the solar protoplanetary disk. *Astrophys. J. Lett.* **827**, L1 (2016).

67. Marrocchi, Y., Villeneuve, J., Batanova, V., Piani, L. & Jacquet, E. Oxygen isotopic diversity of chondrule precursors and the nebular origin of chondrules. *Earth Planet. Sci. Lett.* **496**, 132–141(2018).

68. Nakamura E., et al. On the origin and evolution of the asteroid Ryugu: A comprehensive geochemical perspective. *Proceedings of the Japan Academy, Series B* **98**, 227-282 (2022).

69. Grady M. M., et al. Comparison between carbon and nitrogen in surface and sub-surface materials from asteroid (162173) Ryugu. *Meteorit. Planet. Sci*. **58**, A6194 (2023).

70. Okazaki R., et al. Noble gases and nitrogen in samples of asteroid Ryugu record its volatile sources and recent surface evolution. *Science* **379**, eabo0431 (2023).

71. Yokoyama T., et al. Samples returned from the asteroid Ryugu are similar to Ivuna-type carbonaceous meteorites. *Science* **379**, eabn7850 (2023).

72. Sessions, A. L., Burgoyne, T. W. & Hayes, J. M. Correction of H_3^+ contributions in hydrogen isotope-ratio-monitoring mass spectrometry. *Anal. Chem.* **73**, 192–199 (2001).

73. Alexander, C. M. O'D., Fogel, M., Yabuta, H. & Cody, G. D. The origin and evolution of chondrites recorded in the elemental and isotopic compositions of their macromolecular organic matter. *Geochim. Cosmochim. Acta* **71**, 4380–4403 (2007).

74. Alexander, C. M. O'D., Bowden, R., Fogel, M. L. & Howard, K. T. Carbonate abundances and isotopic compositions in carbonaceous chondrites. *Meteorit. Planet. Sci.* **50**, 810–833 (2015).

75. Dworkin, J. P. et al. OSIRIS-REx Contamination control strategy and implementation. *Space Sci. Rev.* **214**, 19 (2018).

76. Pizzarello, S. The nitrogen isotopic composition of meteoritic HCN. *Astrophys. J.* **796**(2), L25 (2014).

77. Fujiya W. et al. Migration of D-type asteroids from the outer Solar System inferred from carbonate in meteorites. *Nat. Astron.* **3***,* 910–915 (2019).

78. Fujiya, W. et al. Comprehensive study of carbon and oxygen isotopic compositions, trace element abundances, and cathodoluminescence intensities of calcite in the Murchison CM chondrite. *Geochim. Cosmochim. Acta* **161**, 101–117 (2015).

79. Bierhaus, E. B. et al. The OSIRIS-REx spacecraft and the touch-and-go sample acquisition mechanism (TAGSAM). *Space Sci. Rev.* **214**, 1–46 (2018).

80. de Castro, A., Alegre, D. & Tabarés, F. L. Physisorption of ammonia on AISI 304 L stainless steel at different surface temperature under high vacuum conditions. *Nucl. Mater. Energy* **9**, 1–5 (2016).

81. Vinogradoff, S. et al. Influence of phyllosilicates on the hydrothermal alteration of organic matter in asteroids: Experimental perspectives. *Geochim. Cosmochim. Acta* **269**, 150–166 (2020).

82. Aponte J. C., Dworkin, J. P., & Elsila, J. E. Assessing the origins of aliphatic amines in the Murchison meteorite from their compound-specific carbon isotopic ratios and enantiomeric composition. *Geochim. Cosmochim. Acta* **141**, 331–345 (2014).

83. Aponte, J. C. et al. Extraterrestrial organic compounds and cyanide in the CM2 carbonaceous chondrites Aguas Zarcas and Murchison. *Meteorit. Planet. Sci.* **55**, 1509–1524 (2020).

84. Takano, Y., Kashiyama, Y., Ogawa, N. O., Chikaraishi, Y. & Ohkouchi, N. Isolation and desalting with cation-exchange chromatography for compound-specific nitrogen isotope analysis of amino acids: Application to biogeochemical samples. *Rapid Commun. Mass Spectrom.* **24**, 2317–2323 (2010).

85. Oba Y., Takano Y., Dworkin J. P. and Naraoka H. Ryugu asteroid sample return provides a natural laboratory for primordial chemical evolution. *Nat. Commun.* **14**, 3107 (2023).

86. Schmitt-Kopplin, P. et al. Soluble organic matter Molecular atlas of Ryugu reveals cold hydrothermalism on C-type asteroid parent body. *Nat. Commun.* **14**, 6525 (2023).

87. Forster, B., Van De Ville, D., Berent, J., Sage, D. and Unser, M. Complex wavelets for extended depth-of-field: A new method for the fusion of multichannel microscopy images. *Microsc. Res. Tech.* **65**, 33–43 (2004).

88. Hahn, J. H., Zenobi, R., Bada, J. L. and Zare, R. N. Application of two-step laser mass spectrometry to cosmogeochemistry: Direct analysis of meteorites. *Science* **239**, 1523–1525 (1988).

89. Clemett, S. J., Maechling, C. R., Zare, R. N., Swan, P. D. and Walker, R. M. Identification of complex aromatic molecules in individual interplanetary dust particles. *Science* **262,** 721-725 (1993).

90. Clemett, S. J., Sandford, S. A., Nakamura-Messenger, K., Hörz, F. and McKay, D. S. Complex aromatic hydrocarbons in Stardust samples collected from comet 81P/Wild 2. *Meteorit. Planet. Sci.* **45,** 701-722 (2010).

91. Clemett, S. J. and Zare, R. N. Microprobe two-step laser mass spectrometry as an analytical tool for meteoritic samples. In *IAU Symp. 178: Molecules in Astrophysics: Probes & Processes,* p. 305 (1996).

92. Bernstein, M. P., Sandford, S. A., Allamandola, L. J., Gillette, J. S., Clemett, S. J. and Zare, R. N. UV irradiation of polycyclic aromatic hydrocarbons in ices: Production of alcohols, quinones, and ethers. *Science* **283,** 1135-1138 (1999).

93. Hurst, N. and Harilal, S. S. Pulse shaping a transversely excited CO_2 laser using a simple plasma shutter. *Rev. Sci. Instrum.* **80**, 035101 (2009).

94. Lockyer, N. P. and Vickerman, J. C. Single photon ionization mass spectrometry using laser-generated vacuum ultraviolet photons. *Laser Chem.* **17**, 139–159 (1997).

95. Shi, Y. J. and Lipson, R. H. An overview of organic molecule soft ionization using vacuum ultraviolet laser radiation. *Can. J. Chem.* **83**, 1891–1902 (2005).

96. King, B. V., Pellin, M. J., Moore, J. F., Veryovkin, I. V., and Tripa, C. E. Estimation of useful yield in surface analysis using single photon ionization. *Appl. Surf. Sci.* **203-204**, 244–247 (2003).

97. Ferge, T., Mühlberger, F. and Zimmermann, R. Application of infrared laser desorption vacuum-UV single-photon ionization mass spectrometry for analysis of organic compounds from particulate matter filter samples. *Anal. Chem.* **77**, 4528–4538 (2005).

98. Kanno, N. and Tonokura, K. Vacuum ultraviolet photoionization mass spectra and cross-sections for volatile organic compounds at 10.5 eV. *Appl. Spectrosc.*

8, 896–902 (2007).

99. Zimmermann, R., Welthagen, W. and Gröger, T. Photo-ionisation mass spectrometry as detection method for gas chromatography: Optical selectivity and multidimensional comprehensive separations. *J. Chromatogr. A* **1184**, 296–308 (2008).

100. Hanley, L. and Zimmermann, R. Light and molecular ions: The emergence of vacuum UV single-photon ionization in mass spectrometry. *Anal. Chem.* **81**, 4174–4182 (2009).

101. Alexander, C. M. O'D., Bowden, R., Fogel, M. L. & Howard, K., Carbonate abundances and isotopic compositions in chondrites *Meteorit. Planet. Sci.* 50, 810–833 (2014).

102. Kotra, R. K. et al. Amino acids in a carbonaceous chondrite from Antarctica. *J. Mol. Evol.* **13**, 179–183 (1979).

Supplementary Figures

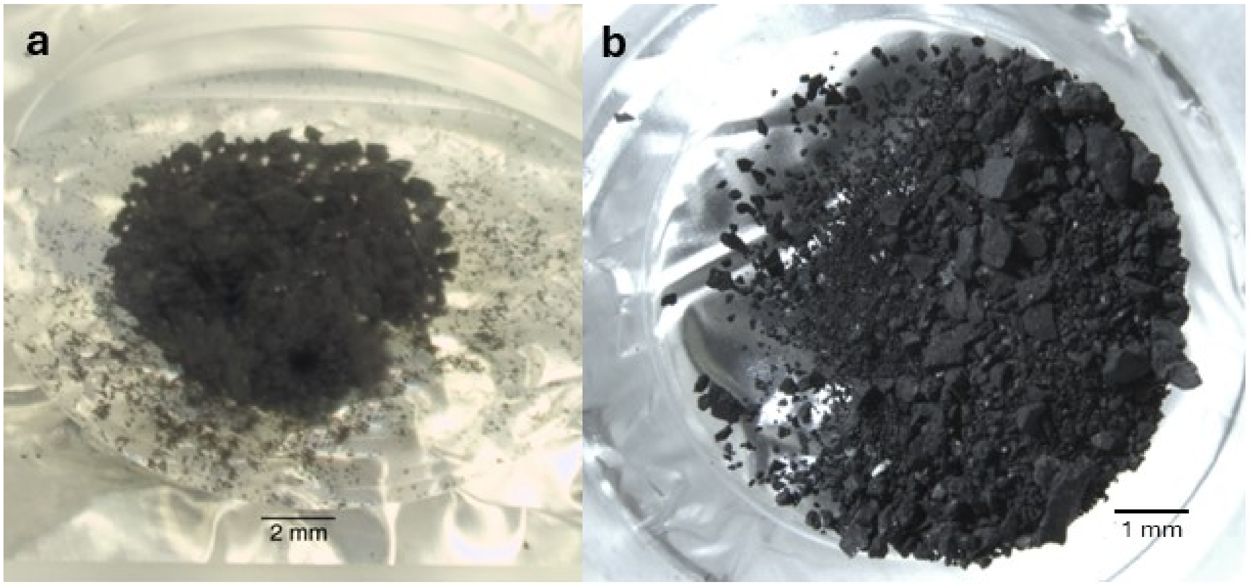

Supplementary Figure 1. Optical images of the Bennu aggregate samples from the TAGSAM head that were placed in sample tray A4 and then allocated for this study. a, Photo of OREX-800031-0 while sealed under nitrogen in between two glass concavity slides before subdivision at GSFC. At the bottom of the image a 2-mm bar is shown for scale. Reflected light from the glass slide in the original photo was digitally removed. Image taken by J. Dworkin at GSFC. **b,** Photo of OREX-800044-101 on a glass slide before extraction. The scale bar corresponds to 1 mm. Image taken by H. Naraoka at KU.

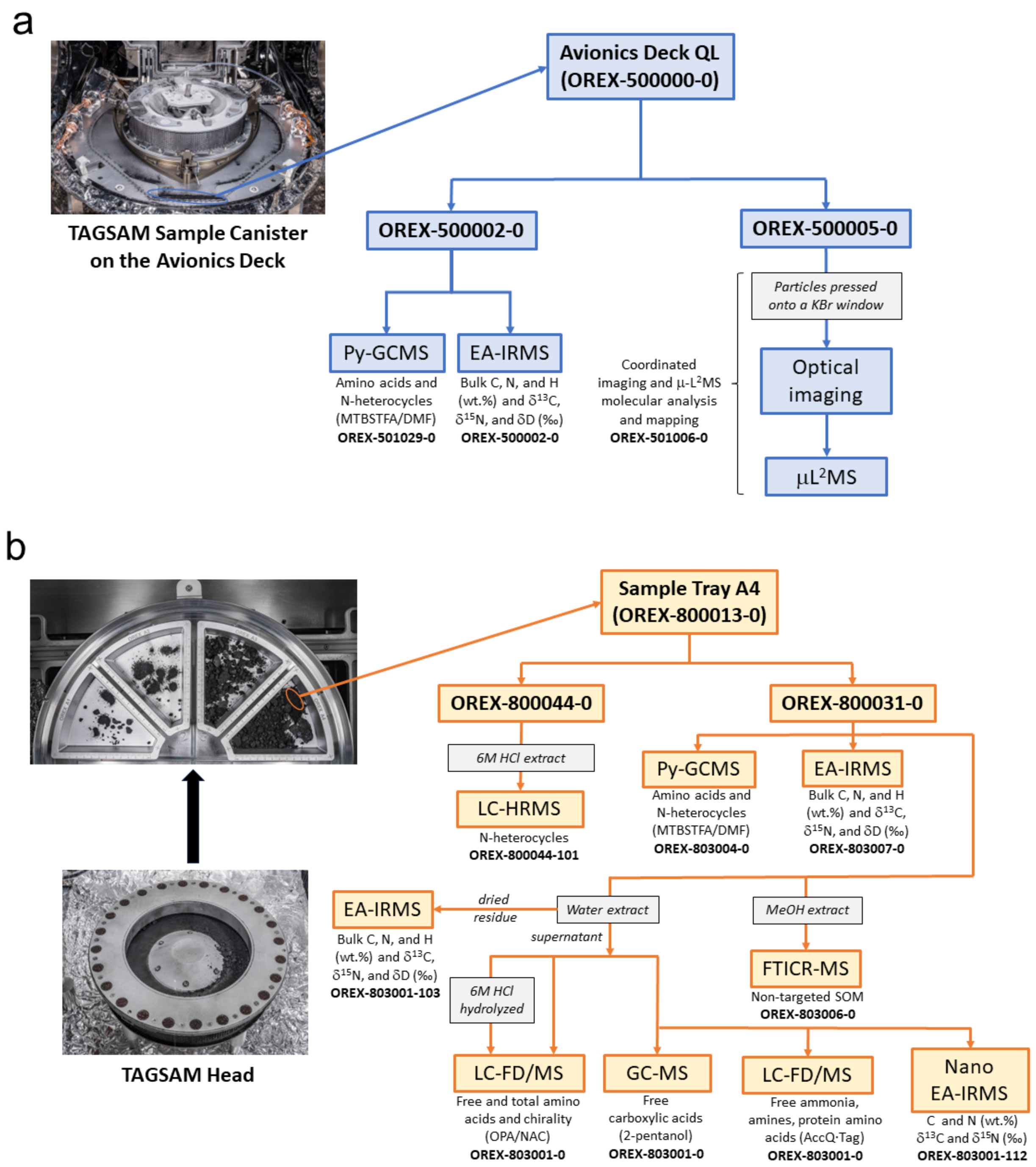

Supplementary Figure 2. Diagram of the processing and analytical scheme for the Bennu aggregate samples used in this investigation. a, Summary of the measurements described in this study that were made on the OREX-500002-0 and OREX-500005-0 aggregate samples that were collected from the surface of the avionics deck outside of the TAGSAM sample collector head and used for "Quick-Look" (QL) analyses and subsample numbers. **b,** Diagram of the analytical flow and measurements made in this study for aggregate samples OREX-800044-0 and OREX-800031-0 that were removed from underneath the Mylar flap of the TAGSAM head and placed in Tray "OREX A4" as OREX-800013-0. Image credits: NASA/Erika Blumenfeld and Joseph Aebersold.

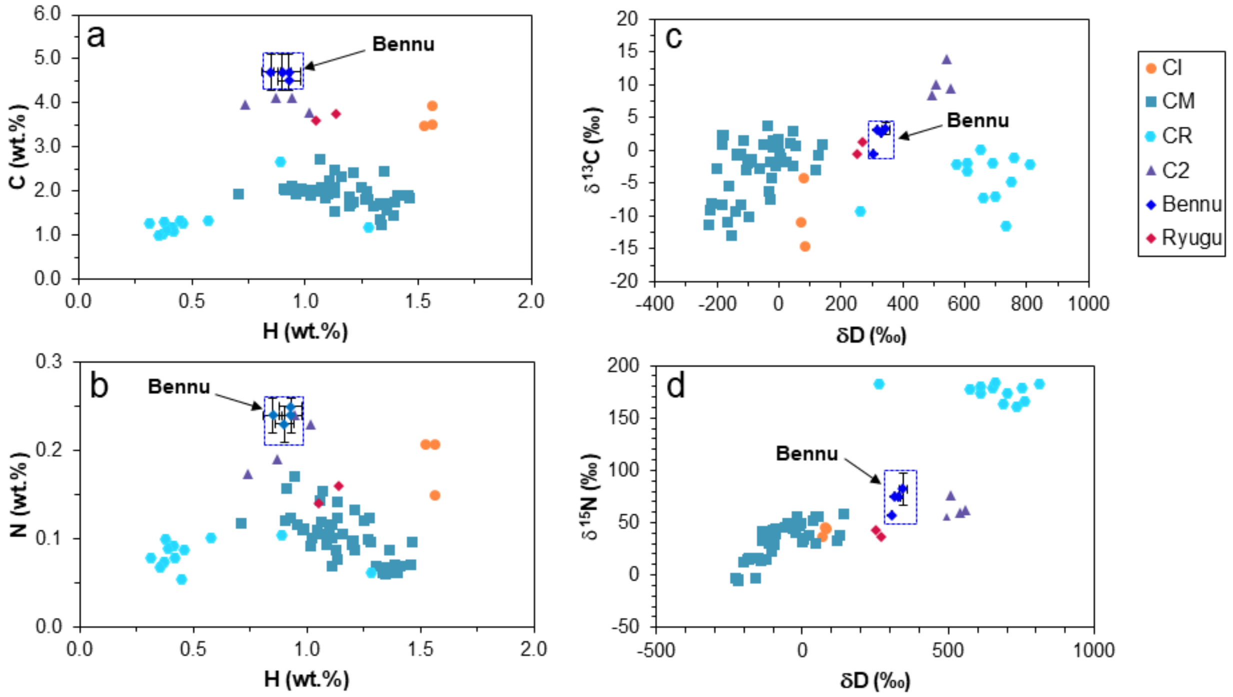

Supplementary Figure 3. Bulk C, N, and H contents and their stable isotopic compositions for the Bennu aggregate samples compared with carbonaceous chondrites and Ryugu. All elemental abundances in wt.% and stable isotopic compositions in ‰ (δ^{13}C, VPDB; δ^{15}N, AIR; δD, VSMOW). **a,** C vs. H (wt.%), **b,** N vs. H (wt.%), **c,** δ^{13}C vs. δD (‰), **d,** δ^{15}N vs. δD (‰). The average values for the four Bennu aggregate samples (OREX-500034/35/38-0, OREX-500036/37/39-0, OREX-500040/41-0, and OREX-803007-0)[1], and data for the carbonaceous chondrites[56,74,101] are based on replicate measurements made using EA-IRMS at CIS (Extended Data Table 1). The data from individual measurements made on subsamples of the Bennu aggregates are given in Supplementary Table 2. Two of the Bennu aggregate samples were heated at 120°C for 48 h under Ar prior to EA-IRMS analysis, and two were not heated, but the differences in C, N and H abundance and isotope values were small (Extended Data Table 1, Supplementary Table 2). Since OREX-803001-103 was extracted in water at 100°C for 24 h and therefore processed differently than the other samples prior to EA-IRMS analyses, the data from this Bennu sample are not included in the plots but are shown in Extended Data Table 1. The average bulk values for Ryugu are from samples A0106 and C0107[20,33]. Standard deviation error bars are shown only for the Bennu data, and the dashed blue box bounds the uncertainty in the measurements. Symbols shown in the legend correspond to different carbonaceous chondrite groups: Ivuna-type (CI), Mighei-type (CM), Renazzo-type (CR), and the ungrouped carbonaceous chondrites ($C2_{ung}$) Tagish Lake and Tarda. Data for the CI, CM, CR, and $C2_{ung}$ chondrites from Alexander et al.[19,56,61] and data first reported in this study from the $C2_{ung}$ Tarda meteorite are also included. The bulk H, C, and N data for these carbonaceous chondrites were obtained from unheated samples.

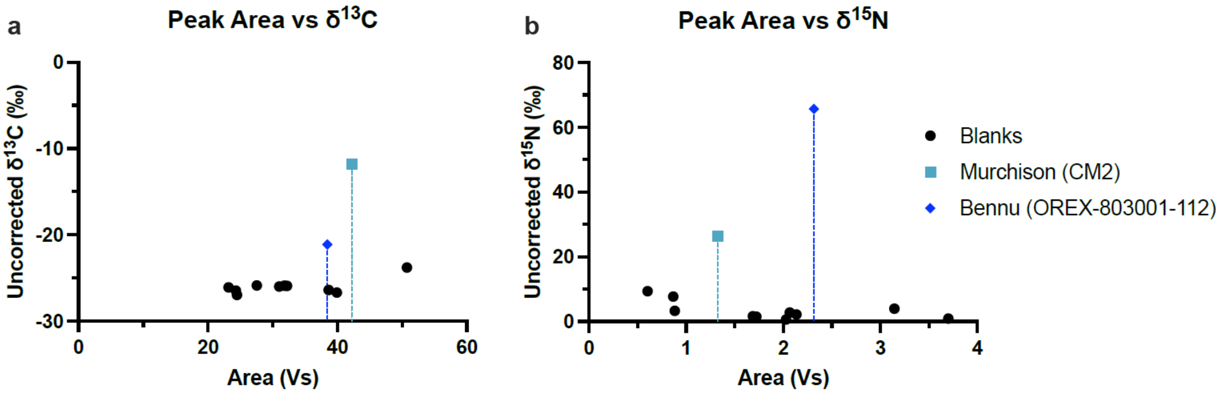

Supplementary Figure 4. Carbon and nitrogen isotope data from the nano EA-IRMS analysis of the Bennu and Murchison water extracts and the blanks. a, Carbon and **b,** nitrogen peak area and $\delta^{13}C$ and $\delta^{15}N$ values isotope results for the nano EA-IRMS system. The uncorrected isotope values are plotted against peak area for the blanks (black circles), CM2 Murchison UIC (aquamarine blue squares), and OREX-803001-112 (blue diamonds). Blanks plotting to the left of the dashed lines (those smaller than the sample) were used for the blank correction.

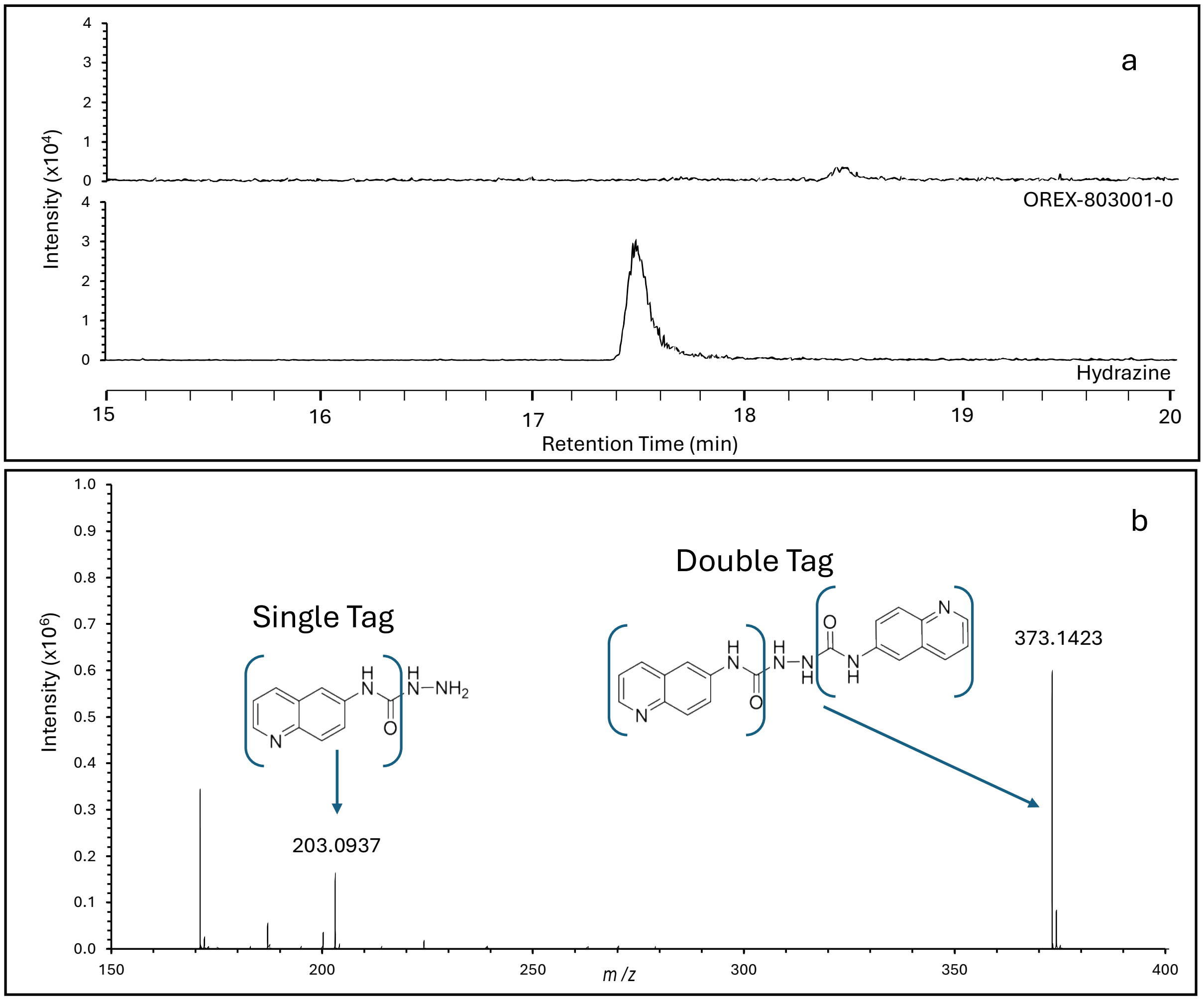

Supplementary Figure 5. LC-MS chromatograms of the 15- to 20-min region of the Bennu (OREX-803001-0) hot-water extract and a hydrazine standard after AccQ·Tag derivatization (10 min). Peaks were not identified by fluorescence since this analyte does not fluoresce with the predetermined excitation and emission wavelengths for AccQ·Tag. Peaks were identified by both single and double "AccQ·tagged" hydrazine. **a,** Mass chromatograms at *m/z* = 373.1423 with a 5-ppm exact mass window at the monoisotopic mass corresponding to the AccQ·Tag derivative of hydrazine for the OREX-803001-0 water extract and the 0.25-μM standard, and **b,** the mass spectrum at 17.45 min for the hydrazine standard with the single AccQ·Tag derivative of hydrazine at *m/z* 203.0937 and the double AccQ·Tag derivative of hydrazine at *m/z* 373.1423. No hydrazine was detected in the Bennu (OREX-803001-0) water extract above the 0.1 nmol/g level (Extended Data Table 2).

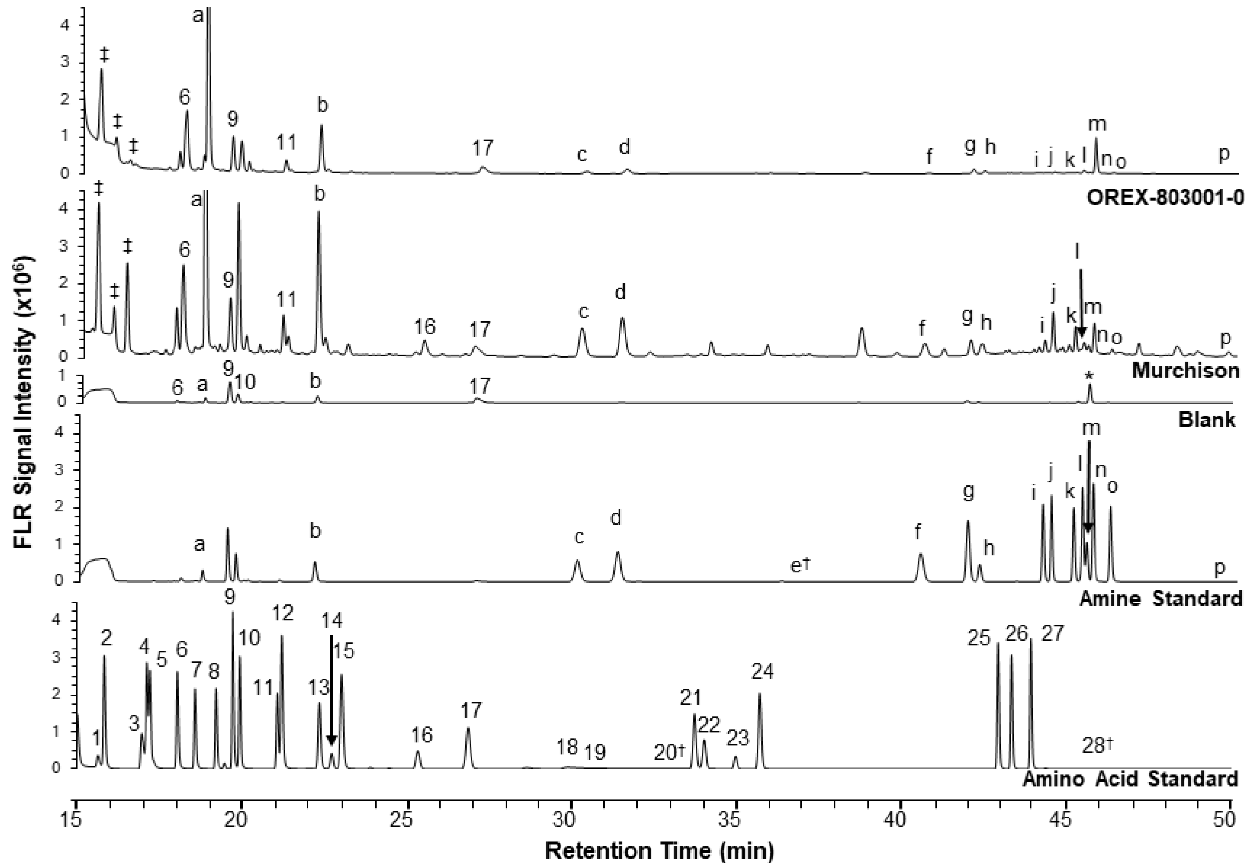

Supplementary Figure 6. Liquid chromatography with UV fluorescence detection chromatograms of free amino acids and amines in the standards and the hot-water extracts of OREX-803001-0, Murchison, and the procedural blank after derivatization (10 min) with AccQ·Tag. Chromatograms of the 15–50 min region from the LC-FD/QqQ-MS analyses. Peaks were identified by comparison to UV fluorescence retention time and molecular mass to those in the standards analyzed on the same day designated by number for amino acids and by letter for the amines. Amino acid peak identifications as follows: (1) histidine, (2) asparagine, (3) arginine, (4) glutamine, (5) serine, (6) glycine, (7) aspartic acid, (8) glutamic acid, (9) β-alanine, (10) threonine, (11) alanine, (12) γ-amino-*n*-butyric acid, (13) β-amino-*n*-butyric acid, (14) proline, (15) β-aminoisobutyric acid, (16) α-aminoisobutyric acid, (17) α-aminobutyric acid, (18) cysteine, (19) lysine, (20) tyrosine, (21) ε-amino-*n*-caproic acid, (22) isovaline, (23) methionine, (24) valine, (25) leucine, (26) isoleucine, (27) phenylalanine, and (28) tryptophan. Amine peak identifications as follows: (a) methylamine, (b) ethylamine, (c) isopropylamine, (d) propylamine, (e) *sec*-butylamine, (f) isobutylamine, (g) *n*-butylamine, (h) *tert*-butylamine, (i) 3-aminopentane, (j) 2-amino-3-methylbutylamine, (k) *sec*-pentylamine, (l) 2-methylbutylamine, (m) *tert*-pentylamine, (n) isopentylamine, (o) *n*-pentylamine, and (p) *n*-hexylamine. †Indicates that the compound does not fluoresce at the excitation and emission wavelengths used for this analysis, however the peak could still be identified and quantified by mass. ‡AccQ·Tag derivatives that were not identified.

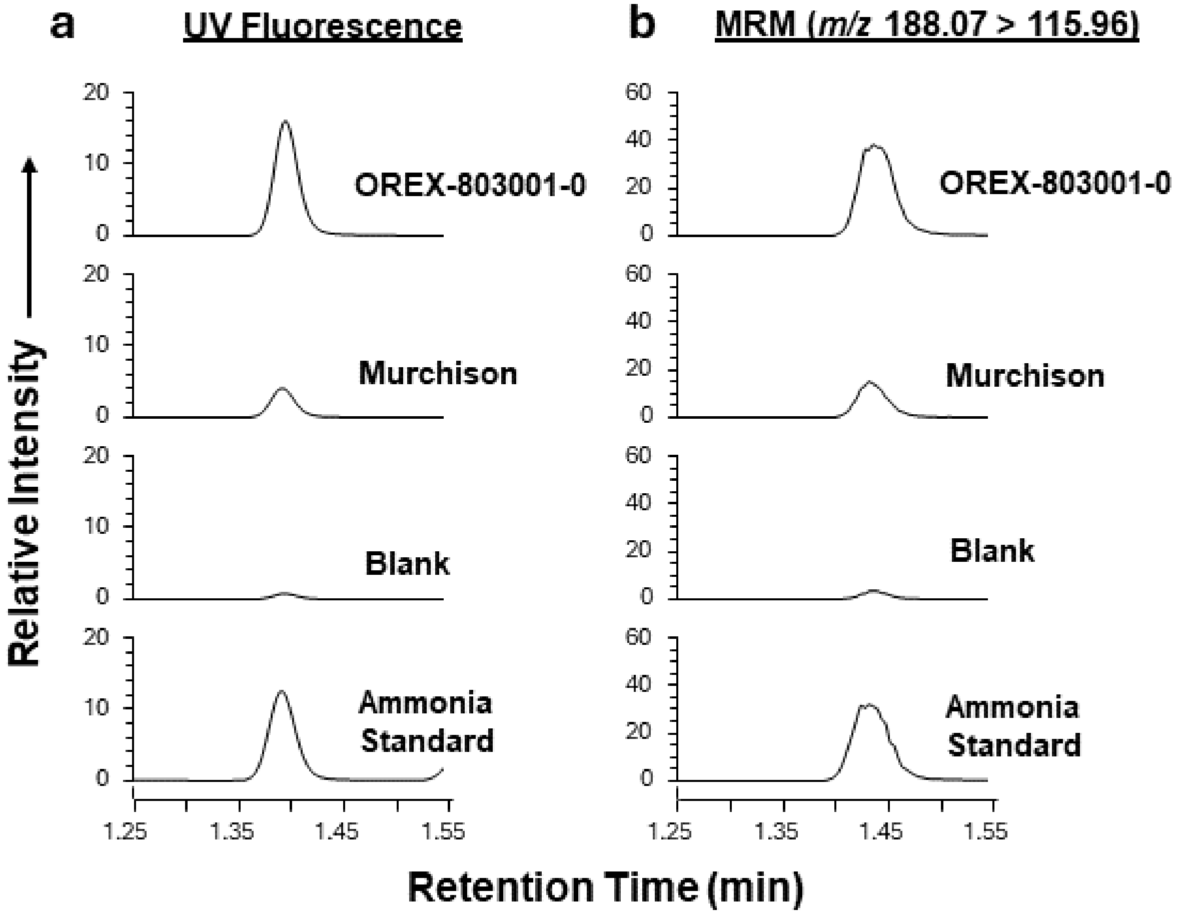

Supplementary Figure 7. LC-FD/QqQ-MS chromatograms showing the identification of the AccQ·Tag derivative of ammonia in the standard and in the hot-water extracts of the procedural blank, the CM2 meteorite Murchison, and Bennu (OREX-803001-0). **a,** UV fluorescence (λ_{ex} = 266 and λ_{em} = 473) traces with a peak at a retention time of ~1.4 min corresponding to ammonia. **b,** Multiple reaction monitoring (MRM) transition (*m/z* 188.07 to 115.96) peak also corresponding to ammonia. Chromatograms only show the 1.25 to 1.55 min region from the 10 min run (other peaks corresponding to AccQ·Tag amine derivatives were detected outside of this range and are discussed elsewhere). The ammonia derivative peaks in the water extracts were identified by comparison of the UV fluorescence retention time and parent to daughter mass transitions of the standard. The MRM data was used for ammonia abundance quantifications.

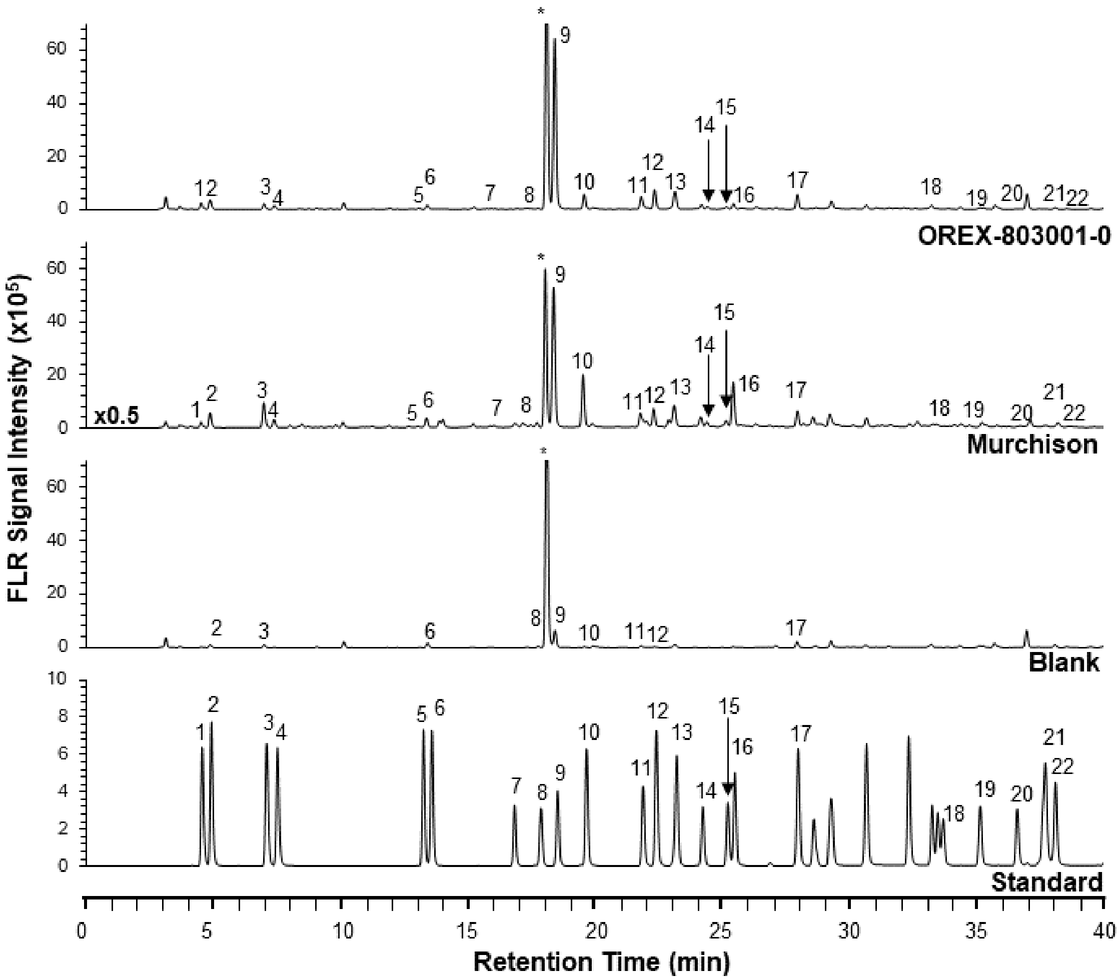

Supplementary Figure 8. Liquid chromatography with UV fluorescence detection chromatograms of amino acids in the standard and the 6 M HCl-hydrolyzed, hot-water extracts of OREX-803001-0, Murchison, and the procedural blank after derivatization (15 min) with *o*-phthaldialdehyde/*N*-acetyl-L-cysteine (OPA/NAC). No peaks were observed beyond a retention time of 40 min. The relative intensity of the Murchison trace was divided by half. Similar chromatograms were also obtained for the non-hydrolyzed water extracts. Peaks were identified by comparison to the fluorescence retention time to those in the amino acid standard analyzed on the same day and are designated by peak number as follows: (1) D-aspartic acid, (2) L-aspartic acid, (3) L-glutamic acid, (4) D-glutamic acid, (5) D-serine, (6) L-serine, (7) D-threonine, (8) L-threonine, (9) glycine, (10) β-alanine, (11) γ-aminobutyric acid, (12) D-alanine, (13) L-alanine, (14) D-β-amino-*n*-butyric acid, (15) L-β-amino-*n*-butyric acid, (16) α-aminoisobutyric acid, (17) D,L-α-amino-*n*-butyric acid, (18) ε-amino-*n*-caproic acid, (19) L-isoleucine, (20) D-isoleucine, (21) D-leucine, and (22) L-leucine. *Analytical artifact peak from the cation exchange desalting resin which did not interfere with the separation and quantification of glycine.

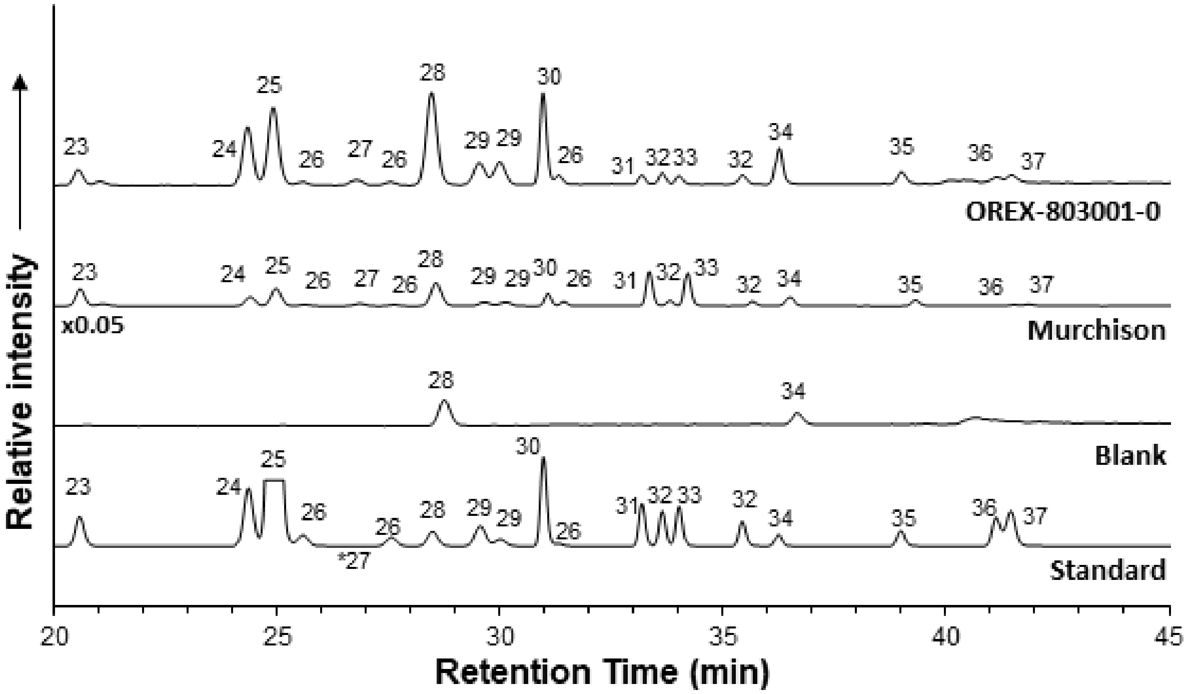

Supplementary Figure 9. LC-ToF-MS chromatograms showing separation of the C_5 amino acids identified in the procedural blank, Bennu (OREX-803001-0), and Murchison meteorite acid-hydrolyzed, hot-water extracts. The 15–40 min region of the LC-ToF-MS single ion mass chromatograms of the C_5 amino acids (m/z = 379.1328) with a mass tolerance of 10 ppm. *o*-phthaldialdehyde/*N*-acetyl-L-cysteine (OPA/NAC) derivatization (15 min) of amino acids in the standard and of the 6 M HCl-hydrolyzed, hot-water extracts of the procedural blank, Murchison (intensity divided by 20), and OREX-803001-0. Similar chromatograms were obtained for the non-hydrolyzed water extracts. Peaks were identified by comparison of the single ion mass chromatogram retention time to those in the amino acid standard analyzed on the same day and are designated by peak number as follows: (23) 3-amino-2,2-dimethylpropanoic acid, (24) D,L-4-aminopentanoic acid, (25) D,L-4-amino-3-methylbutanoic acid, (26) D,L- and D,L-*allo*-3-amino-2-methylbutanoic acid, (27) D,L-3-amino-2-ethylpropanoic acid, (28) 5-aminopentanoic acid, (29) D,L-4-amino-2-methylbutanoic acid, (30) 3-amino-3-methylbutanoic acid, (31) D-isovaline, (32) (R)-3-aminopentanoic acid, (33) L-isovaline, (34) (S)-3-aminopentanoic acid, (35) L-valine, (36) D-valine, (37) D-norvaline, and (38) L-norvaline.

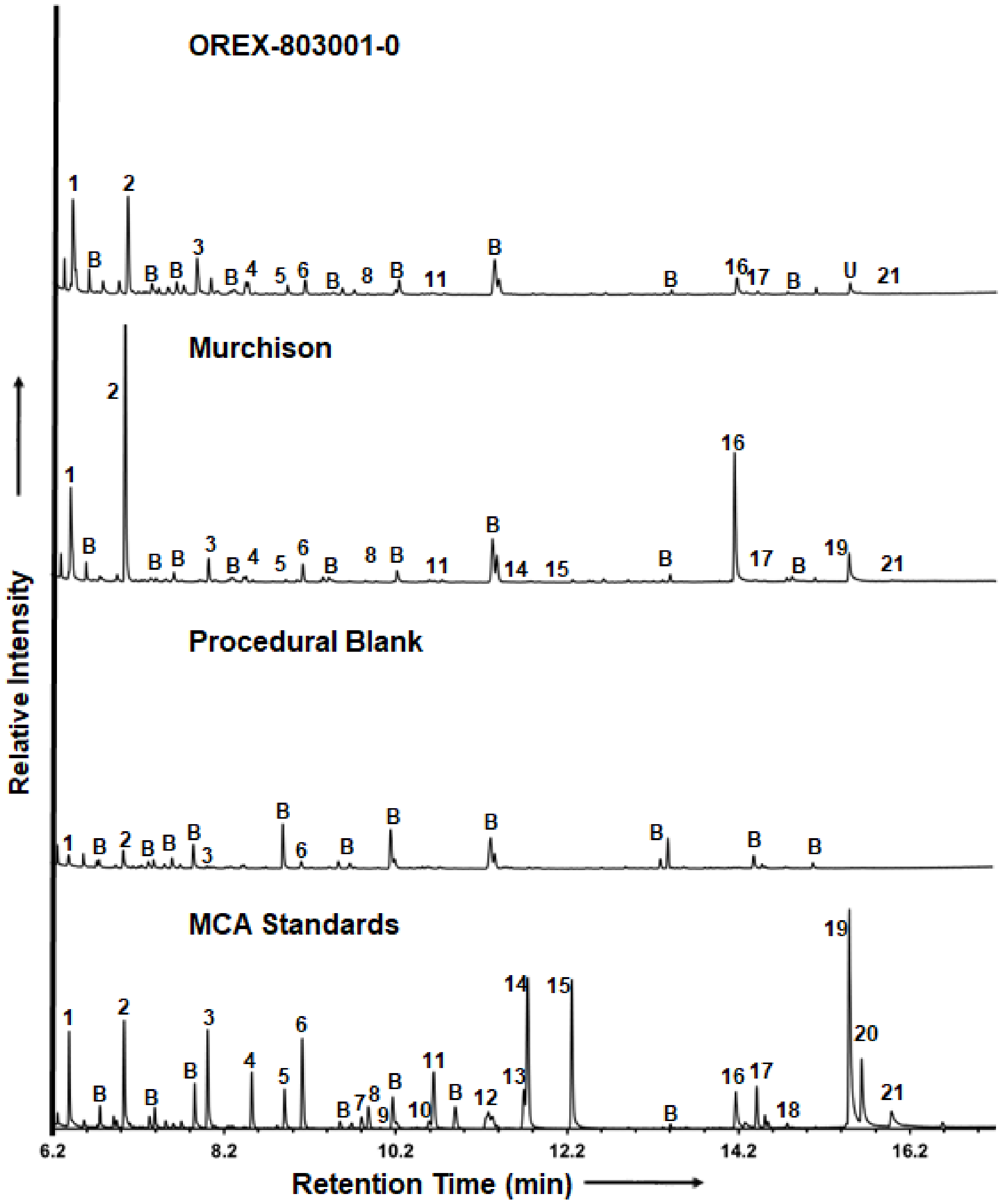

Supplementary Figure 10. GC-QqQ-MS mass chromatograms of carboxylic acids identified in the Bennu and Murchison hot-water extracts. Positive electron-impact GC-QqQ-MS chromatogram (6.2 –17.2 min region, *m/z* = 55 + 60 + 70 + 81 + 89 + 99 + 101 + 105 + 169) of 2-pentanol derivatized carboxylic acids from the hot-water extract of OREX-803001-0, the CM2 Murchison meteorite, a procedural blank, and commercially available standards (all traces excepting standards are on the same intensity scale). Peak identifications as follows: (1) formic acid, (2) acetic acid, (3) propanoic acid, (4) isobutyric acid, (5) 2,2-dimethylpropanoic acid, (6) butyric acid, (7) 2-methylbutyric acid, (8) isopentanoic acid, (9) 2,2-dimethylbutyric acid, (20) 3,3-dimethylbutyric acid, (11) pentanoic acid, (12) 2-ethylbutyric and 2-methylpentanoic acids, (13) 3-methylpentanoic acid, (14) 4-methylpentanoic acid, (15) hexanoic acid, (16) oxalic acid, (17) benzoic acid, (18) malonic acid, (19) succinic acid, (20) fumaric/maleic acid, (21) glutaric acid, (B) reaction byproduct (ethers formed from the excess alcohol used for esterification), and (U) unknown compound.

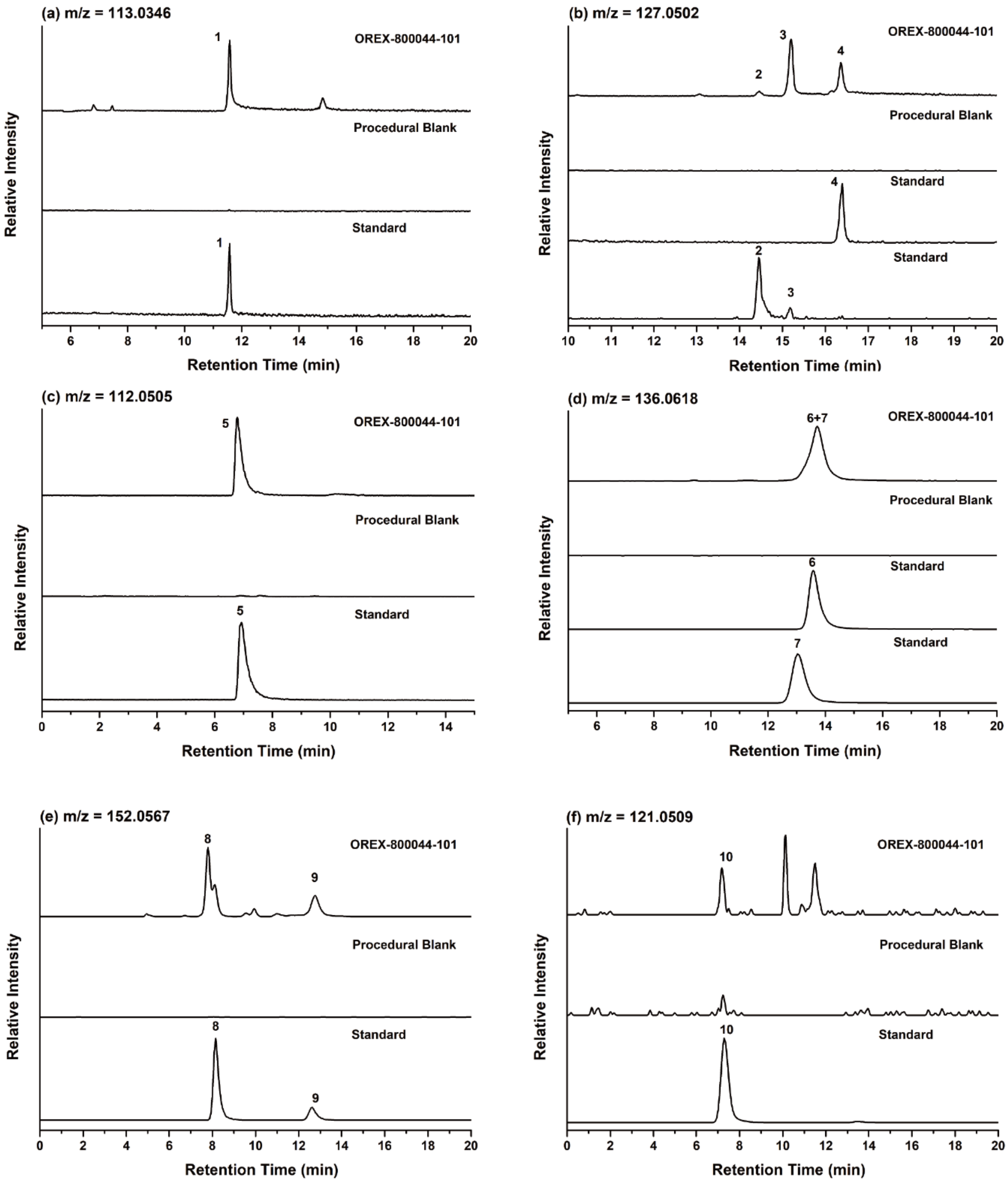

Supplementary Figure 11. High-resolution mass chromatograms of nitrogen heterocycles in the Bennu sample OREX-800044-101 and blank compared to standards. The mass-to-charge (*m*/*z*) ratios correspond to: (a) uracil, (b) thymine, (c) cytosine, (d) adenine, (e) guanine, and (f) purine. Peaks were identified in the samples by comparison of the retention time and molecular mass to those in standards analyzed on the same day and are designated by peak number as follows: (1) uracil, (2) 1-methyluracil, (3) 6-methyluracil, (4) thymine, (5) cytosine, (6) adenine, (7) 8-aminpurine, (8) guanine, (9) isoguanine, and (10) purine. The presence of guanine was also confirmed by the MS/MS measurement (Supplementary Fig. 13).

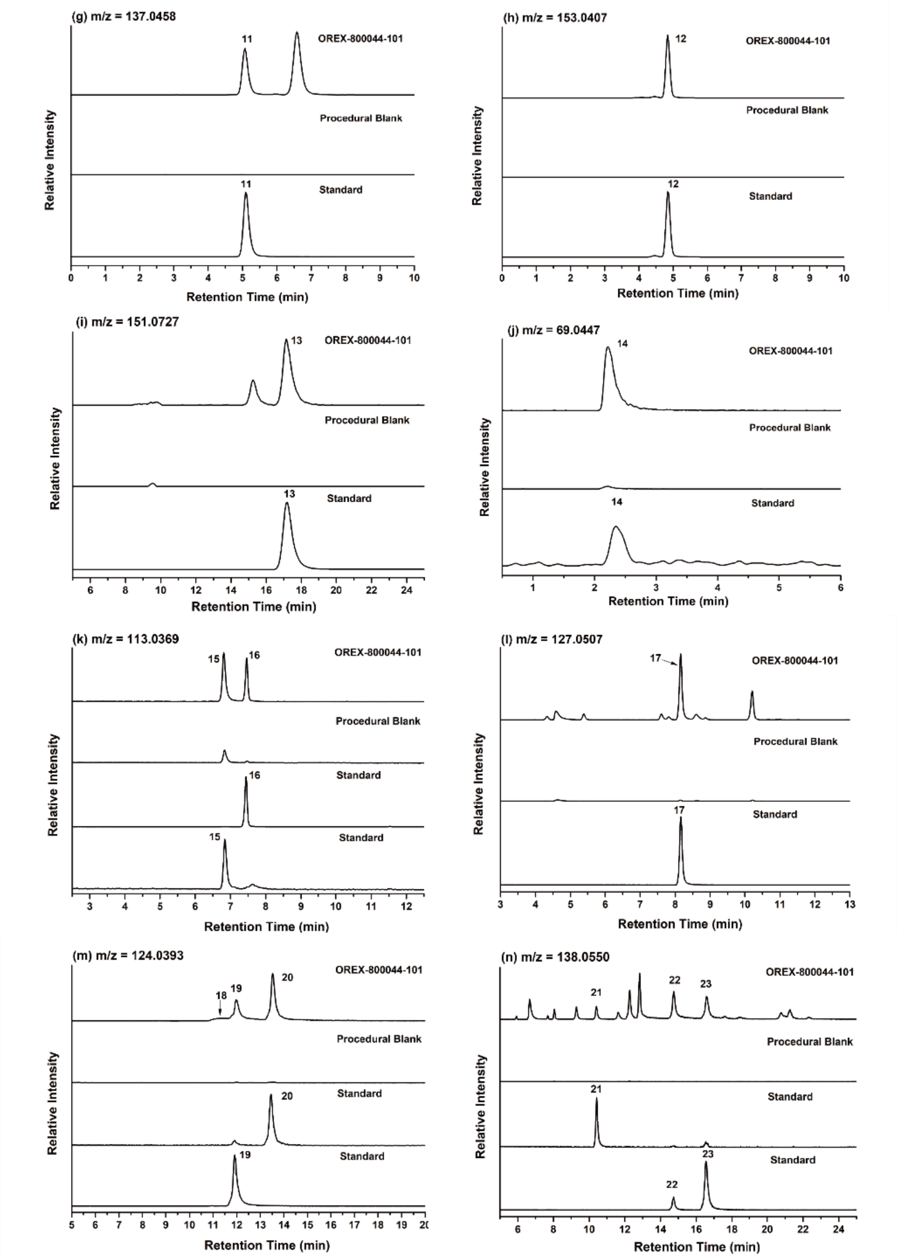

Supplementary Figure 12. High-resolution mass chromatograms of nitrogen heterocycles in the Bennu sample OREX-800044-101, the blanks, and standards. The mass-to-charge (*m/z*) ratios shown correspond to: (g) hypoxanthine, (h) xanthine, (i) diaminopurines, (j) imidazole, (k) imidazole carboxylic acids, (l) methylimidazole carboxylic acids, (m) nicotinic acid, and (n) methylnicotinic acid. Most peaks were identified in the samples by comparison of the retention time and molecular mass to those in standards analyzed on the same day and are designated by peak number as follows: (11) hypoxanthine, (12) xanthine, (13) 2,6-diaminopurine, (14) imidazole, (15) 4-imidazolecarboxylic acid, (16) 2-imidazolecarboxylic acid, (17) 2-methyl-1H-imidazole-4-carboxylic acid, (18) picolinic acid, (19) isonicotinic acid, (20) nicotinic acid, (21) 2-methylnicotinic acid, (22) 6-methylnicotinic acid, and (23) 5-methylnicotinic acid. The imidazole and picolinic acid standards were not analyzed on the same day as the Bennu samples, but the retention times were consistent with those measured previously under the same analytical conditions[63]. The 2,6-diaminopurine peak (13) may co-elute with 6,8-diaminopurine[63].

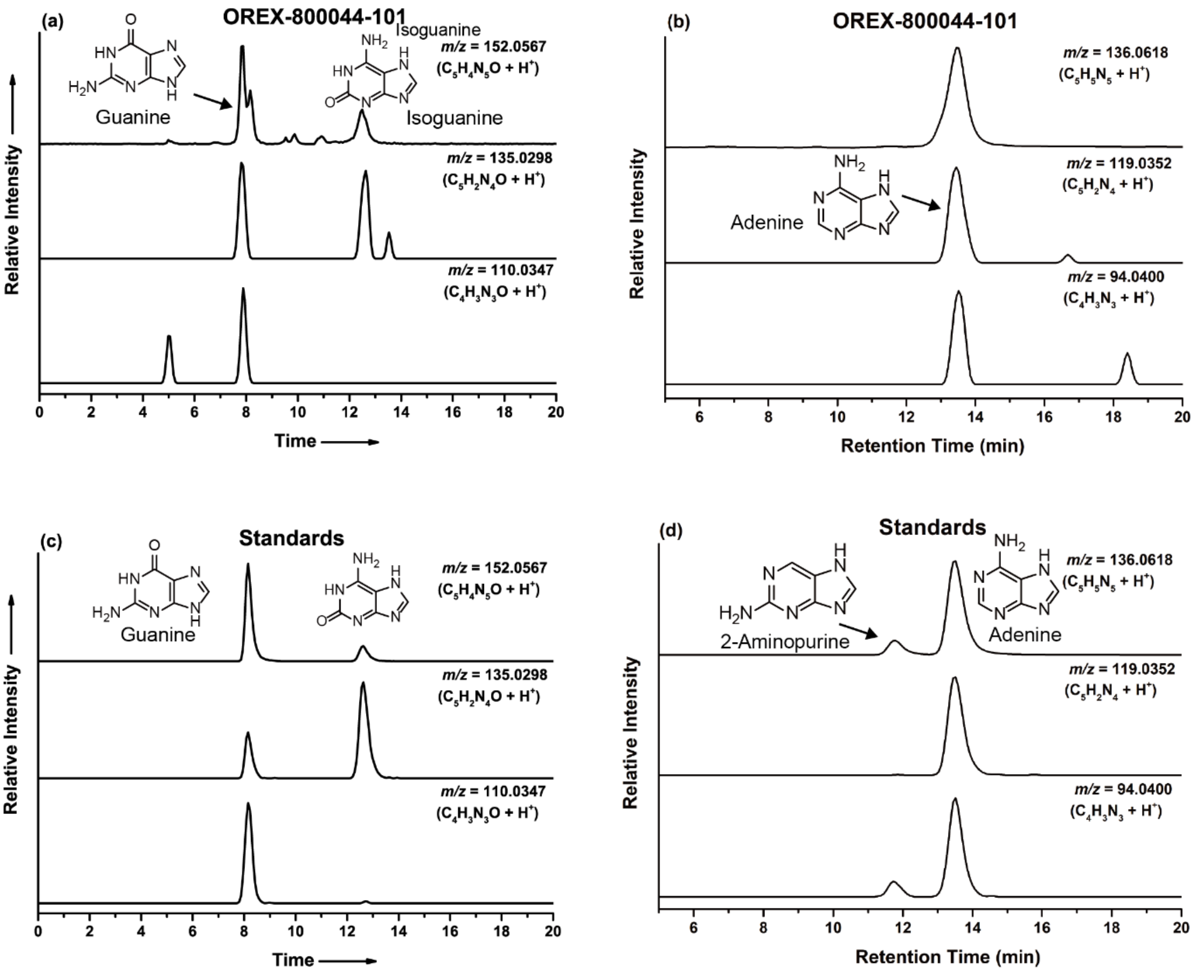

Supplementary Figure 13. High-resolution mass chromatograms of selected purines in the Bennu sample OREX-800044-101, the blanks, and standards. Mass chromatograms at the mass-to-charge (*m/z*) ratio corresponding to the parent ion of **a** guanine and isoguanine and **b** adenine and 2-aminopurine, as well as their daughter in the Bennu sample OREX-800044-101. Those for the **c** guanine and **d** adenine standards are also shown for comparison.

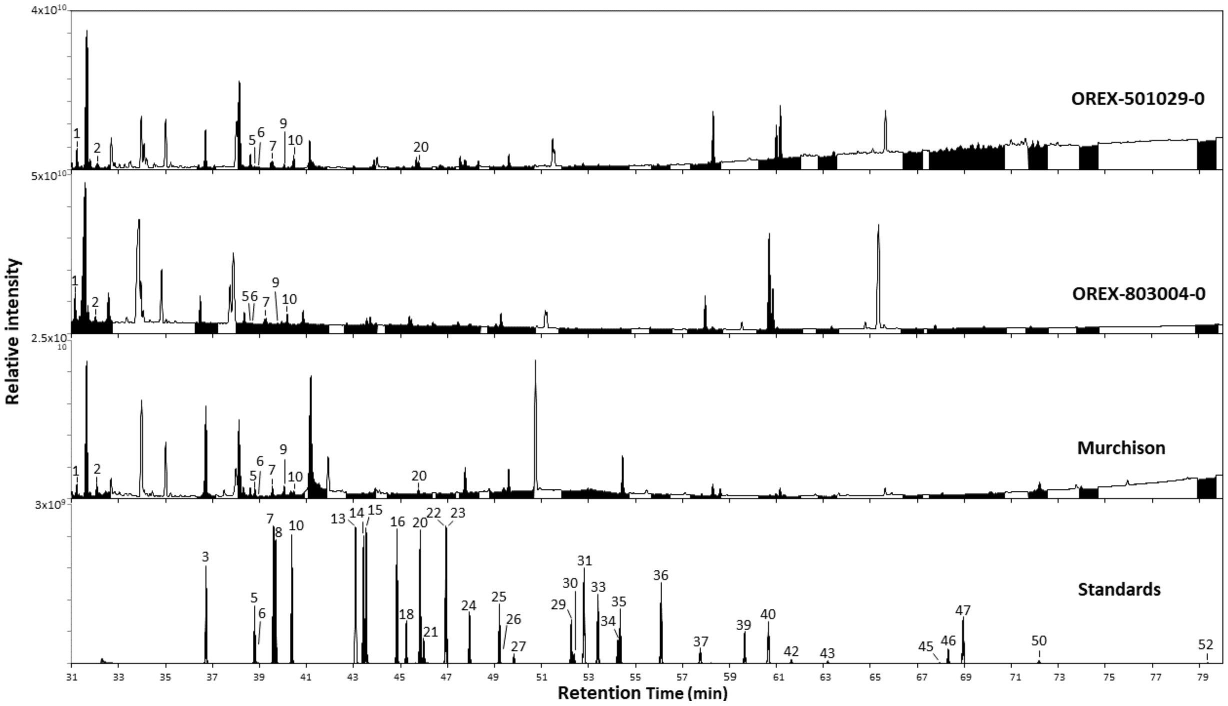

Supplementary Figure 14. Total-ion chromatograms (full scan and multiple reaction monitoring, MRM) showing the detection of amino acids and N-heterocycles after wet-chemistry pyrolysis GC-QqQ-MS analyses of OREX-501029-0, OREX-803004-0, the Murchison meteorite, and the standards. All samples were heated in a solution of MTBSFTA/DMF (4:1 v/v) at 85°C for 90 min prior to pyrolysis GC-QqQ-MS analyses. The amino acid and N-heterocycles in the samples were identified as their *tert*-butyldimethylsilyl (*t*BDMS) derivatives from the peak retention times and individual MRM scans compared to standards as described in Supplementary Table 2 and as follows: (1) 4(3*H*)-pyrimidinone, (2) imidazole, (3) 1-methyl-1*H*-pyrazole-5-carboxylic acid, (4) 2-ethyl-4-methylimidazole, (5) isonicotinic acid, (6) isocytosine, (7) alanine, (8) nicotinic acid/picolinic acid, (9) 2-methylalanine, (10) glycine, (11) 2-aminobutanoic acid, (12) β-alanine, (13) picolinamide, (14) urea, (15) valine, (16) leucine, (17) nicotinamide, (18) isonicotinamide, (19) 2,4-diaminopyrimidine, (20) isoleucine, (21) purine, (22) proline, (23) uracil, (24) 6-methyluracil, (25) thymine, (26) 1-methyluracil, (27) isocytosine, (28) 1*H*-imidazole-2-carboxylic acid, (29) cytosine, (30) pyroglutamic acid, (31) methionine, (32) 5-methylcytosine, (33) serine, (34) threonine, (35) 2,4-diaminopyrimidine, (36) phenylalanine, (37) aspartic acid, (38) 4-imidazole-carboxylic acid, (39) hypoxanthine, (40) glutamic acid, (41) asparagine, (42) adenine, (43) lysine, (44) 2,6-diaminopurine (2-*t*BDMS), (45) histidine, (46) xanthine, (47) tyrosine, (48) tryptophan (2-*t*BDMS), (49) guanine, (50) 2,6-diaminopurine (3-*t*BDMS), (51) tryptophan (3-*t*BDMS), and (52) cysteine.

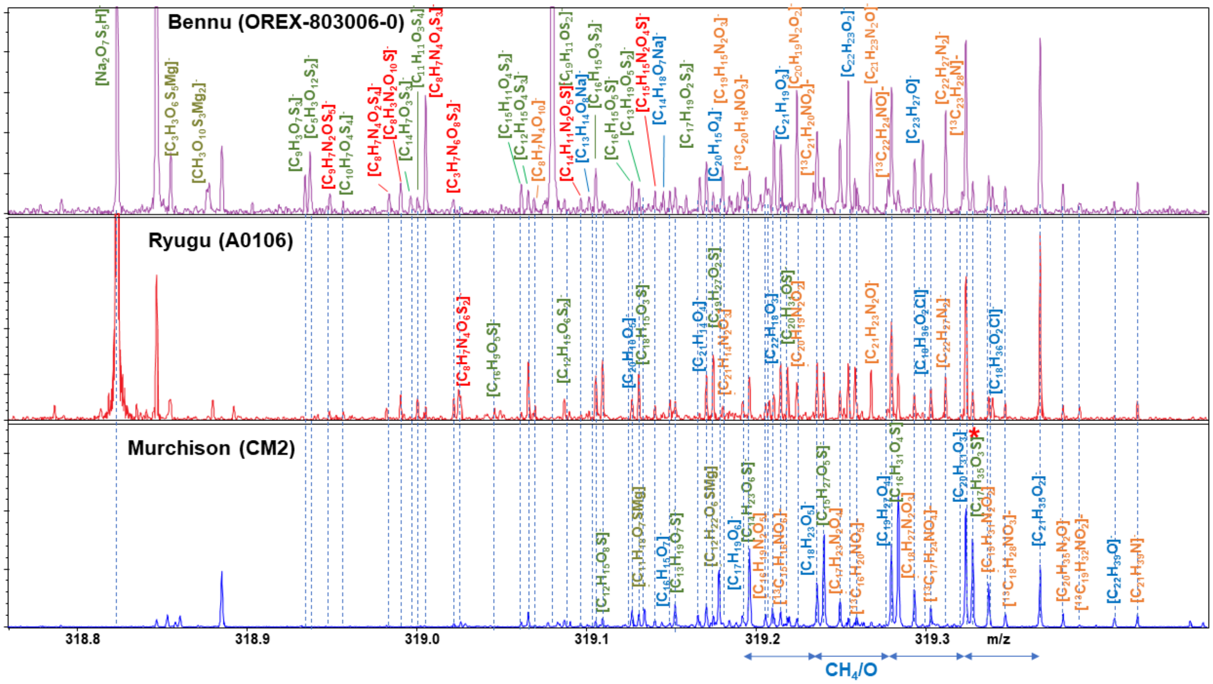

Supplementary Figure 15. FTICR-MS mass spectrum at nominal mass *m/z* 319 with annotated mass signals of Bennu (OREX-803006-0), Ryugu (A0106), and the Murchison (CM2) meteorite. The high signal density and systematic homologous series typical of complex organic mixtures is shown. Such a homologous series of sulfur-containing CHOS, for example, ends from 8 oxygen ($[C_{12}H_{15}O_8S]^-$) down to 3 oxygen ($[C_{17}H_{35}O_3S]^-$), indicating that these may be $-SO_3$ substituted molecules. Color code: blue, CHO; green, CHOS; orange, CHNO; and red, CHNOS.

Supplementary Tables

Supplementary Table 1. Description of the Bennu parent aggregate samples and subsamples and the sample preparation and analytical techniques used in this study.

Sample ID (mass) and Parent ID	Sample Description & Container	Subsample or split ID (mass)	Sample Preparation	Techniques Used
**OREX-500002-0**<br>(22 mg)<br>Parent:<br>OREX-500000-0<br>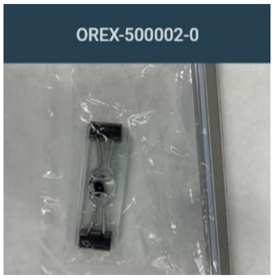 	Avionics deck aggregate mixture of mostly dark fines (<100 μm) and some intermediates (100–500 μm), some bright and highly reflective particles, others with a metallic luster, and numerous (>5) fibers; sealed under N_2 in concavity slide	OREX-501029-0 (1.1 mg)	Added 5 μl MTBSTFA:DMF (4:1 v/v) and heat at 85°C for 1.5 h; pyrolysis at 250°C	GC-QqQ-MS
		Multiple subsamples of OREX 500002-0: OREX-501034-0 to OREX-501041-0 (0.9–5.5 mg ea.)	Heated at 120°C for 24 h under Ar or under Ar at room temperature for 66 h	EA-IRMS
**OREX-500005-0**<br>(88 mg)<br>Parent:<br>OREX-500000-0<br>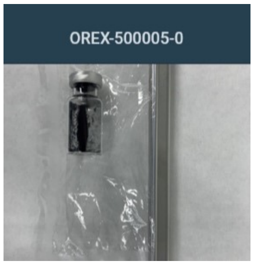 	Avionics deck aggregate mixture of mostly dark fines with average grain size <100 μm, with some particles up to 500 μm. Some bright and highly reflective particles mixed in; sealed under N_2 in glass vial with Viton stopper and crimped Al lid.	OREX-501006-0 (<1 mg)	Several <100 μm grains pressed onto a KBr window	Coordinated optical and UV fluorescence imaging and μ-L^2MS molecular analysis and mapping

**OREX-800044-0**<br>(109 mg)<br>Parent:<br>OREX-800013-0<br>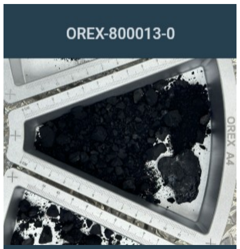<br>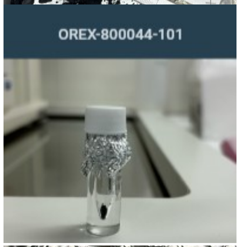<br>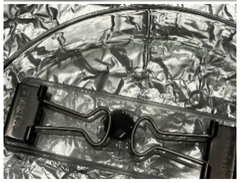	Bulk aggregate parent sample removed from underneath the TAGSAM Mylar flap and placed in deep Tray OREX A4. Mixture of mostly dark fines (<100 μm) and intermediates (100–500 μm) with some coarse particles (>500 μm) and several mm-sized particles. This sample has mostly black particles, but rare light-colored particles are present; sealed under N_2 in glass concavity slide placed inside an Eagle Stainless.	OREX-800044-101 (17.75 mg)	110°C for 12 h extraction in 6 M HCl under N_2	LC-HRMS
**OREX-800031-0**<br>(52 mg)<br>Parent:<br>OREX-800013-0<br>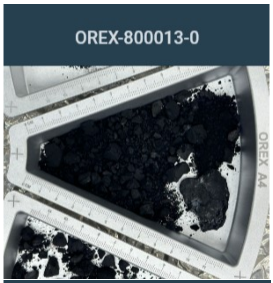<br>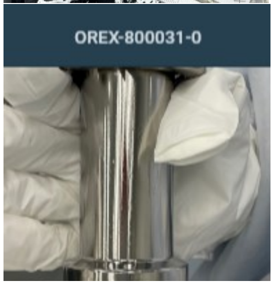	Bulk aggregate parent sample removed from underneath the TAGSAM Mylar flap and placed in deep Tray OREX A4. Composed primarily of fine (<100 μm) and intermediate (100–500 μm) sized particles with some coarse particles (>500 μm). This sample includes mostly black particles with rare (but present in low abundance) light-colored particles; sealed under N_2 in a glass concavity slide placed in an Eagle Stainless container.	OREX-803001-0 (25.6 mg)	100°C for 24 h extraction in water, water extract split for coordinated SOM analyses	LC-MS, LC-HRMS, GC-QqQ-MS
		OREX-803001-112 (extract, n/a)	~2.6% split of water extract, added 2 μL 6 M HCl to water extract and dried under vacuum at room temperature in tin capsule	NanoEA-IRMS
		OREX-803001-103 (19.2 mg)	Dried residue after water extraction, heated at 120°C for 24 h under Ar	EA-IRMS
		OREX-803004-0 (1.0 mg)	Added 5 μL MTBSTFA:DMF (4:1 v/v) and heat at 85°C for 1.5 h; pyrolysis at 250°C under He	GC-QqQ-MS
		OREX-803006-0 (3.3 mg)	Sample crushing in methanol at room temperature	FTICR-MS
		OREX-803007-0 (23.6 mg)	Heated at 120°C for 24 h under Ar	EA-IRMS

Supplementary Table 2. Comparison of the bulk C, N, and H contents and their stable isotopic compositions of the individual subsamples of the Bennu aggregates. Measurement of the elemental abundances (wt.%) and stable isotopic compositions (‰ $\delta^{13}C$ vs. VPDB; $\delta^{15}N$ v. AIR; δD vs. VSMOW) of the bulk samples were conducted by using EA-IRMS at the Carnegie Institution for Science (CIS). The masses of each subsample used for the measurements are also shown. The reported errors are 1-sigma and correspond to the highest variability observed in the standards.

Bennu Aggregate Subsample	Mass (mg)	C (wt.%)	$\delta^{13}C$ (‰)	N (wt.%)	$\delta^{15}N$ (‰)	H (wt.%)	δD (‰)
**Bennu (OREX-500002-0, Avionics Deck)**							
**OREX-501034-0**[a] (fines, <0.1 mm)	0.877					0.84 ± 0.05	332 ± 2
**OREX-501035-0**[a] (fines, <0.1 mm)	0.878					0.86 ± 0.05	328 ± 2
**OREX-501036-0**[b] (fines, <0.1 mm)	0.930					0.90 ± 0.05	314 ± 2
**OREX-501037-0**[b] (fines, <0.1 mm)	0.934					0.89 ± 0.05	315 ± 2
**OREX-501040-0**[b] (intermediate, 0.2 mm)	0.992					0.93 ± 0.05	305 ± 2
**OREX-501038-0**[a] (fines, <0.1 mm)	5.238	4.7 ± 0.4	2.7 ± 0.1	0.24 ± 0.02	74.6 ± 0.1		
**OREX-501039-0**[b] (fines, <0.1 mm)	5.533	4.7 ± 0.4	3.2 ± 0.1	0.23 ± 0.02	75.5 ± 0.1		
**OREX-501041-0**[b] (intermediate, 0.2 mm)	2.281	4.7 ± 0.4	–0.5 ± 0.1	0.24 ± 0.02	57.1 ± 0.1		
**Bennu (OREX-803007-0, TAGSAM aggregate, <0.5 mm)**							
**OREX-803040-0**[a]	1.420					0.93 ± 0.03	325 ± 2
**OREX-803041-0**[a]	1.343					0.95 ± 0.03	359 ± 2
**OREX-803042-0**[a]	1.440					0.93 ± 0.03	344 ± 2
**OREX-803043-0**[a]	1.359					0.92 ± 0.03	365 ± 2
**OREX-803002-0**[a]	2.161					0.95 ± 0.03	327 ± 2
**OREX-803044-0**[a]	5.244	4.6 ± 0.2	4.4 ± 0.2	0.25 ± 0.01	70.4 ± 0.2		
**OREX-803045-0**[a]	5.411	4.4 ± 0.2	1.9 ± 0.2	0.25 ± 0.01	68.3 ± 0.2		
**OREX-803046-0**[a]	5.176	4.7 ± 0.2	3.5 ± 0.2	0.25 ± 0.01	106.0 ± 0.2		
**OREX-803001-103 (residue after water extraction @ 100°C 24h)**							
**OREX-803001-104**[a]	1.179					0.99 ± 0.03	288 ± 2
**OREX-803001-105**[a]	1.501					0.99 ± 0.03	295 ± 2
**OREX-803001-106**[a]	1.523					1.05 ± 0.03	297 ± 2
**OREX-803001-107**[a]	0.990					1.03 ± 0.03	295 ± 2
**OREX-803001-108**[a]	4.755	4.3 ± 0.2	–3.0 ± 0.2	0.20 ± 0.01	58.8 ± 0.2		
**OREX-803001-109**[a]	4.994	4.3 ± 0.2	–1.3 ± 0.2	0.20 ± 0.01	56.8 ± 0.2		
**OREX-803001-110**[a]	4.297	4.2 ± 0.2	–2.3 ± 0.2	0.20 ± 0.01	58.5 ± 0.2		

[a]Sample heated at 120°C for 48 h under Ar (<0.1 ppm H_2O and O_2) in a glovebox, and then kept there at room temperature for 66 hours without exposure to atmosphere prior to EA-IRMS analysis.
[b]Sample under Ar in glovebox at room temperature without any exposure to atmosphere prior to EA-IRMS analysis.

Supplementary Table 3. Summary of previously reported Ryugu bulk C, N, and H contents and stable isotopic compositions of aggregate samples and their calculated mass weighted average values. Measurement of the elemental abundances (wt.%) and stable isotopic compositions (‰ $\delta^{13}C$ vs. VPDB; $\delta^{15}N$ v. AIR; δD vs. VSMOW) of the samples were conducted by EA-IRMS. The masses of each sample used to calculate the weighted average values for each set of published data are also shown.

Ryugu Aggregate Sample	Mass (mg)[a]	C (wt.%)	$\delta^{13}C$ (‰)	N (wt.%)	$\delta^{15}N$ (‰)	Mass (mg)[b]	H (wt.%)	δD (‰)
**Oba et al.[33]**								
**C0107**	0.0450	3.36	-7.4	0.12		0.5276	1.12	255
**C0107**	0.1305	3.24	-1.3	0.13	39.0	0.3272	1.10	281
**C0107**	0.1600	3.47	-2.1	0.14	32.6	0.2642	0.94	270
**C0107[c]**	0.1696	4.27	15.7	0.15	38.8			
Weighted average		***3.67***	***4.6***	***0.14***	***36.7***		***1.07***	***266***
**Naraoka et al.[20]**								
**A0106**	0.1820	3.69	-2.7	0.16	39.1	0.1348	1.05	240
**A0106**	0.1019	3.93	1.4	0.17	53.2	0.0965	1.15	265
**A0106**	0.1279	3.68	-0.4	0.16	36.7	0.4241	1.22	250
Weighted average		***3.75***	***-0.9***	***0.16***	***42.0***		***1.17***	***250***
**Okazaki et al.[70]**								
**A0105-07**	0.118	6.8		0.070	1.7			
**C0106-07**	0.119	6.4		0.084	0.0			
**A0105-05**	0.140			0.089	18.1			
**C0106-06**	0.168			0.086	19.5			
Weighted average		***6.6***		***0.08***	***11.5***			
**Yokoyama et al.[71]**								
**A0040**	0.91	4.67				0.91	0.94	
Weighted average		***4.67***					***0.94***	
**Grady et al.[69]**								
**A0219[d]**	2	6	11	0.22	43			
**C0208**	1.7	2.9	0.0	0.0923	36.5			
**C0209**	2.5	3.8	-1.7	0.1491	29.4			
Weighted average		***3.44***	***-1.1***	***0.13***	***31.5***			
**Nakamura et al.[68]**								
**A0022**	0.557	4.02	14.1	0.12	40.5	0.137	1.11	178
**A0033**	0.532	5.39	-2.4	0.17	17.8	0.172	0.694	202
**A0035**	0.131	4.12	7.1	0.19	52.1	0.074	1.30	158
**A0048**	0.578	3.39	-10.3	0.13	35.0	0.119	1.12	218
**A0073**	0.113	3.40	-9.7	0.19	52.3			
**A0078**	0.757	3.48	-9.4	0.18	50.9	0.124	1.03	183
**A0085**						0.057	0.98	301
**C0008**	0.765	3.70	-3.0	0.16	46.2	0.317	0.974	341
**C0019**	0.583	3.17	-6.3	0.10	26.8	0.121	1.02	100
**C0027**	0.538	3.16	-7.3	0.10	22.7	0.147	1.07	123
**C0039**	0.173	3.34	-7.7	0.11				
**C0047**	0.103	2.79	-10.5	0.10				
**C0053**						0.135	1.22	210
**C0079**	0.571	3.24	-9.0	0.11	22.9	0.145	1.14	159
**C0081**	0.568	5.22	-9.8	0.22	53.0	0.130	1.07	345
**C0082**	0.650	5.34	-15.3	0.19	0.4	0.126	0.937	108
Weighted average		***3.95***	***-6.1***	***0.15***	***33.2***		***1.03***	***212***
Weighted average of all values		***3.81***	***-3.5***	***0.13***	***33.1***		***1.04***	***236***

[a]Sample mass used for C and N measurements.
[b]Sample mass used for H measurements.
[c]Carbonate-rich.
[d]Data excluded from mass weighted average due to large (± 30%) uncertainties in sample mass and measured abundances.

Supplementary Table 4. Comparison of the total C and N contents and their stable isotopic compositions of the Bennu aggregate and CM2 meteorite Murchison water extracts. All elemental abundances in nmol and stable isotopic compositions in ‰ ($\delta^{13}C$ vs. VPDB; $\delta^{15}N$ vs. AIR) were measured on the nano-EA-IRMS system at The Pennsylvania State University (PSU). The reported measured isotope values have been corrected for blank contribution using Equation 2, and the associated errors were determined using Equation 3. The Murchison nitrogen peak was below the detection limit. The calculated elemental abundances and stable isotope values were determined by mass balance using the elemental abundances and stable isotope values of Bennu and Murchison aggregate powder measured by EA-IRMS at CIS before and after hot-water extraction (see Extended Data Table 1). The amount of carbon lost is the difference between the calculated and measured values, and the $\delta^{13}C$ value of the carbon lost was calculated by mass balance using the measured and calculated abundances and isotope values. Uncertainties in the calculated values and carbon loss were determined by standard error propagation.

Subsample	$\delta^{13}C$ (‰, VPDB)		C (nmol)		Carbon lost	
	measured	calculated	measured	calculated	C (nmol)	$\delta^{13}C$ (‰, VPDB)
**OREX-803001-112** (hot-water extract from aggregate, <0.5 mm)	–9 ± 3	80 ± 77	25 ± 53	166 ± 155	141 ± 164	96 ± 169
**Murchison (CM2)** (hot-water extract from powder)	23 ± 9	61 ± 35	28 ± 53	107 ± 62	79 ± 82	78 ± 109

Subsample	$\delta^{15}N$ (‰, AIR)		N (nmol)	
	measured	calculated	measured	calculated
**OREX-803001-112** (hot-water extract from aggregate, <0.5 mm)	180 ± 47	178 ± 86	6 ± 35	24 ± 5
**Murchison (CM2)** (hot-water extract from powder)	55 ± 8[a]	80 ± 31	4 ± 35[a]	13 ± 4

[a]Below detection limit of nano-EA-IRMS system, therefore low confidence in value.

Supplementary Table 5. Uncorrected nitrogen and carbon peak areas and stable isotope values of the blanks and the dried water extracts of the Bennu aggregate (OREX-803001-112) and Murchison meteorite. All peak areas (Vs) and stable isotopic values (‰; uncorrected) were measured on the nano-EA-IRMS system at Pennsylvania State University. The mean peak area and mean isotope value of the capsule + water + acid blanks smaller than samples were used for data correction.

Sample	$\delta^{13}C$ (‰)	Peak Area (Vs)	$\delta^{15}N$ (‰)	Peak Area (Vs)
Empty Capsule Blank	–25.8	33.2	4.8	2.8
Empty Capsule Blank	–25.5	36.0	10.0	1.5
Capsule + Water + Acid	–25.9	32.2	0.7	2.0
Capsule + Water + Acid	–27.0	24.5	2.2	2.1
Capsule + Water + Acid	–26.0	31.0	9.4	0.6
Capsule + Water + Acid	–26.4	38.7	7.7	0.9
Capsule + Water + Acid	–25.9	31.8	2.8	2.1
Capsule + Water + Acid	–26.7	39.9	3.3	0.9
Capsule + Water + Acid	–25.9	27.6	4.0	3.1
Capsule + Water + Acid	–23.8	50.7	0.9	3.7
Capsule + Water + Acid	–26.5	24.3	1.7	1.7
Capsule + Water + Acid	–26.1	23.2	1.5	1.7
Fused silica FS120	–26.4	42.8	8.5	0.9
Procedural Blank	–26.6	26.6	4.3	0.7
Murchison (CM2) (hot-water extract of powder)	–11.8	42.2	26.4	1.3
OREX-803001-112 (hot-water extract from of aggregate, <0.5 mm)	–21.1	38.4	65.7	2.3

Supplementary Table 6. Detection metrics observed when analyzing a mixed amino acid standard using the analytical technique described in this study for hydrazine. After derivatization with AccQ·Tag, the mass shifted by either 171 or 171×2 Da.

	Molecular Weight of untagged analyte (g/mol)	Chemical Formula	Theoretical *m/z*	Experimental *m/z*
Hydrazine (Single Tag)	32.0452	$C_{10}H_{11}N_4O$	203.0928	203.0937
Hydrazine (Double Tag)	32.0452	$C_{20}H_{17}N_6O_2$	373.1408	373.1423

Supplementary Table 7. Multiple reaction monitoring (MRM) parameters used for the LC-QqQ-MS peak identifications and quantifications of the AccQ·Tag derivatives of amino acids. All three traces were used for compound identifications, and the first precursor ion to product ion (*m/z*) mass transition (Quant. Trace) was used for quantification.

RT (min)	Name	Quant. Trace (m/z)	1° Trace (m/z)	2° Trace
15.17	Histidine	326.10 > 156.10	326.13 > 155.97	An3 - FLR
16.01	Asparagine	303.10 > 171.10	303.10 > 116.10	An3 - FLR
17.09	Arginine	345.10 > 175.10	345.20 > 70.00	An3 - FLR
17.27	Glutamine	317.10 > 171.10	317.10 > 145.10	An3 - FLR
17.38	Serine	276.07 > 170.94	276.10 > 116.10	An3 - FLR
18.20	Glycine	246.10 > 171.10	246.10 > 89.10	An3 - FLR
18.72	Aspartic Acid	304.05 > 171.01	304.10 > 116.10	An3 - FLR
19.34	Glutamic Acid	318.10 > 171.00	318.10 > 116.20	An3 - FLR
19.86	Alanine	260.07 > 170.93	260.07 > 116.10	An3 - FLR
20.07	Threonine	290.13 > 170.95	290.13 > 115.96	An3 - FLR
21.20	β-Alanine	260.07 > 170.93	260.07 > 116.10	An3 - FLR
21.36	γ-Amino-*n*-butyric Acid	274.10 > 171.11	274.10 > 116.10	An3 - FLR
22.48	β- Amino-*n*-butyric Acid	274.10 > 171.11	274.10 > 116.10	An3 - FLR
22.85	Proline	286.13 > 170.90	286.13 > 115.91	An3 - FLR
23.15	β-Aminoisobutyric Acid	274.10 > 171.11	274.10 > 116.10	An3 - FLR
25.46	α-Aminoisobutyric Acid	274.10 > 171.11	274.10 > 116.10	An3 - FLR
26.97	α-Amino-*n*-butyric Acid	274.10 > 171.11	274.10 > 116.10	An3 - FLR
28.68	Cysteine	581.10 > 171.10	581.10 > 145.10	An3 - FLR
29.84	Lysine	487.13 > 171.04	487.13 > 116.17	An3 - FLR
33.84	Tyrosine	352.13 > 170.94	352.13 > 116.07	[a]
33.84	ε-Amino-*n*-caproic Acid	302.13 > 170.94	302.13 > 116.01	An3 - FLR
34.14	Isovaline	288.13 > 170.95	288.10 > 89.10	An3 - FLR
35.09	Methionine	320.13 > 170.93	320.13 > 116.01	An3 - FLR
35.83	Valine	288.13 > 170.95	288.10 > 116.10	An3 - FLR
43.07	Leucine	302.13 > 170.94	302.13 > 116.01	An3 - FLR
43.48	Isoleucine	302.13 > 170.94	302.13 > 116.01	An3 - FLR
44.06	Phenylalanine	336.10 > 171.10	336.10 > 116.10	An3 - FLR
44.50	Tryptophan	375.10 > 171.10	375.10 > 89.10	[a]

[a]AccQ·Tag derivative peak not observed at the UV detector excitation and emission wavelengths (λ_{ex} = 266 nm; λ_{em} = 473 nm). FLR = fluorescence signal.

Supplementary Table 8. Multiple reaction monitoring (MRM) parameters used for the LC-QqQ-MS peak identifications and quantifications of the AccQ·Tag derivatives of ammonia and the amines. All three traces were used for compound identifications, and the first precursor ion to product ion (*m/z*) mass transition (Quant. Trace) was used for quantification.

RT (min)	Name	Quant. Trace (*m/z*)	1° Trace (*m/z*)	2° Trace
15.01	Ammonia	188.07 > 115.96	188.07 > 89.02	An3 - FLR
18.91	Methylamine	202.13 > 170.91	202.13 > 115.97	An3 - FLR
22.31	Ethylamine	216.20 > 115.95	216.20 > 89.00	An3 - FLR
30.33	Isopropylamine	230.20 > 170.89	230.20 > 115.96	An3 - FLR
31.56	Propylamine	230.20 > 170.89	230.20 > 115.96	An3 - FLR
36.44	*sec*-Butylamine	244.20 > 170.91	244.20 > 115.97	[a]
40.68	Isobutylamine	244.20 > 170.91	244.20 > 115.97	An3 - FLR
42.04	*n*-Butylamine	244.20 > 170.91	244.20 > 115.97	An3 - FLR
42.39	*tert*-Butylamine	244.20 > 170.91	244.20 > 115.97	An3 - FLR
44.28	3-Aminopentane	258.20 > 170.94	258.20 > 115.95	An3 - FLR
44.54	2-Amino-3-methylbutylamine	258.20 > 170.94	258.20 > 115.95	An3 - FLR
45.20	*sec*-Pentylamine	258.20 > 170.94	258.20 > 115.95	An3 - FLR
45.48	2-Methylbutylamine	258.20 > 170.94	258.20 > 115.95	An3 - FLR
45.80	*tert*-Pentylamine	258.20 > 170.94	258.20 > 115.95	An3 - FLR
45.81	Isopentylamine	258.20 > 170.94	258.20 > 115.95	An3 - FLR
46.32	*n*-Pentylamine	258.20 > 170.94	258.20 > 115.95	An3 - FLR
51.64	*n*-Hexylamine	272.13 > 170.95	272.13 > 115.96	An3 - FLR

[a]AccQ·Tag derivative peak not observed at the UV detector excitation and emission wavelengths (λ_{ex} = 266 nm; λ_{em} = 473 nm). FLR = fluorescence signal.

Supplementary Table 9. Detection metrics observed for selected C2 to C6 amino acids using the LC-FD/ToF-MS analytical technique. As a result of derivatization with OPA/NAC, 261 Da is added to the measured mass of each amino acid. Mass error was calculated using the following equation:

$$mass\ error = \frac{experimental\ mass - theoretical\ mass}{theoretical\ mass} * 1e^{6}$$

Analyte	FD RT (min)	MS RT (min)	$[M+H]^+$ Chemical Formula	Theoretical *m/z*	Experimental *m/z*	Mass Error (ppm)
D-Aspartic acid	4.55	4.69	$C_{17}H_{19}N_2O_7S$	395.0913	395.0916	0.7593
L-Aspartic acid	4.92	5.06	$C_{17}H_{19}N_2O_7S$	395.0913	395.0915	0.5062
L-Glutamic acid	7.08	7.23	$C_{18}H_{21}N_2O_7S$	409.1069	409.1068	0.2444
D-Glutamic acid	7.52	7.65	$C_{18}H_{21}N_2O_7S$	409.1069	409.1072	0.7333
D-Serine	13.25	13.4	$C_{16}H_{19}N_2O_6S$	367.0964	367.0962	0.5448
L-Serine	13.58	13.73	$C_{16}H_{19}N_2O_6S$	367.0964	367.0966	0.5448
D-Threonine	16.83	16.98	$C_{17}H_{21}N_2O_6S$	381.1120	381.1117	0.7872
L-Threonine	17.87	18.01	$C_{17}H_{21}N_2O_6S$	381.1120	381.1118	0.5248
Glycine	18.53	18.67	$C_{15}H_{17}N_2O_5S$	337.0858	337.0845	3.8565[a]
β-Ala	19.67	19.81	$C_{16}H_{19}N_2O_5S$	351.1015	351.1014	0.2848
γ-ABA	21.88	22.03	$C_{17}H_{21}N_2O_5S$	365.1171	365.1179	2.1911[a]
D-Alanine	22.42	22.56	$C_{16}H_{19}N_2O_5S$	351.1015	351.1023	2.2785[a]
L-Alanine	23.2	23.35	$C_{16}H_{19}N_2O_5S$	351.1015	351.1021	1.7089
D-β-ABA	24.23	24.38	$C_{17}H_{21}N_2O_5S$	365.1171	365.1172	0.2739
L-β-ABA	25.22	25.37	$C_{17}H_{21}N_2O_5S$	365.1171	365.1172	0.2739
α-AIB	25.5	25.64	$C_{17}H_{21}N_2O_5S$	365.1171	365.1168	0.8217
D,L-α-ABA	27.98	28.13	$C_{17}H_{21}N_2O_5S$	365.1171	365.1166	1.3694
ε-ACA	29.28	29.46	$C_{19}H_{25}N_2O_5S$	393.1484	393.1484	0.0000
L-Isoleucine	35.15	35.3	$C_{19}H_{25}N_2O_5S$	393.1484	393.1477	1.7805
D-Isoleucine	36.6	36.76	$C_{19}H_{25}N_2O_5S$	393.1484	393.1478	1.5261
D-Leucine	37.7	37.85	$C_{19}H_{25}N_2O_5S$	393.1484	393.1477	1.7805
L-Leucine	38.1	38.27	$C_{19}H_{25}N_2O_5S$	393.1484	393.1477	1.7805

[a]Glycine, D-alanine, and γ-ABA all have peaks in the mass spectra that are leading to higher mass errors. These peaks are fully separated by the Xevo G2 XS time of flight in the mass spectra and in all FWHM resulting chromatograms, but the mass experimental *m/z* calculation completed by the Masslynx software takes these smaller peaks into account.

Supplementary Table 10. Detection metrics observed for the C5 amino acids using the LC-FD/ToF-MS analytical technique. After derivatization with OPA/NAC, 261 Da is added to the measured mass of each amino acid. Mass error was calculated using the following equation:

$$mass\ error = \frac{experimental\ mass - theoretical\ mass}{theoretical\ mass} * 1e^{6}$$

Analyte	FD RT (min)	MS RT (min)	$[M+H]^+$ Chemical Formula	Theoretical *m/z*	Experimental *m/z*	Mass Error (ppm)
3-A-2,2-DMPA	20.47	20.59	$C_{18}H_{23}N_2O_5S$	379.1328	379.1330	0.5275
D,L-4-APA	24.22	24.36	$C_{18}H_{23}N_2O_5S$	379.1328	379.1326	0.5275
D,L-4-A-3-MBA	24.78	24.95	$C_{18}H_{23}N_2O_5S$	379.1328	379.1333	1.3188
D,L-and *allo*-3-A-2-MBA	25.45	25.60	$C_{18}H_{23}N_2O_5S$	379.1328	379.1324	1.0550
D,L-3-A-2-EPA	26.50	26.50	$C_{18}H_{23}N_2O_5S$	379.1328	n.d.	-
δ-AVA	28.33	28.51	$C_{18}H_{23}N_2O_5S$	379.1328	379.1324	1.0550
D,L-4-A-2-MBA	29.40	29.57	$C_{18}H_{23}N_2O_5S$	379.1328	379.1325	0.7913
3-A-3-MBA	30.82	30.98	$C_{18}H_{23}N_2O_5S$	379.1328	379.1327	0.2638
D-Iva	33.03	33.20	$C_{18}H_{23}N_2O_5S$	379.1328	379.1325	0.7913
L-3-APA	33.48	33.65	$C_{18}H_{23}N_2O_5S$	379.1328	379.1321	1.8463
L-Iva	33.85	34.00	$C_{18}H_{23}N_2O_5S$	379.1328	379.1322	1.5826
D-3-APA	35.27	35.42	$C_{18}H_{23}N_2O_5S$	379.1328	379.1321	1.8463
L-Val	36.05	36.25	$C_{18}H_{23}N_2O_5S$	379.1328	379.1326	0.5275
D-Val	38.82	39.01	$C_{18}H_{23}N_2O_5S$	379.1328	379.1321	1.8463
D-Nva	40.95	41.12	$C_{18}H_{23}N_2O_5S$	379.1328	379.1321	1.8463
L-Nva	41.28	41.46	$C_{18}H_{23}N_2O_5S$	379.1328	379.1325	0.7913

n.d. = not determined due to degradation of the standard which is not commercially available. Therefore, the neighboring peak consisting of D,L- and *allo*-3-A-2-MBA analyte was used for quantitation of this amino acid in the samples.

Supplementary Table 11. Summary of the wet-chemistry pyrolysis GC-QqQ-MS peak identifications of the amino acid and N-heterocycles identified in the Bennu samples and Murchison. Identification of the *tert*-butyldimethylsilyl (tBDMS) derivatives was made based on retention times (min) and precursor to product ion (*m/z*) mass transitions used in multiple reaction monitoring (MRM) mode for the Murchison meteorite, reagent blank, and Bennu aggregate samples (OREX-803004-0 and OREX-501029-0).

Peak #	Analyte	Retention Time (min)	Precursor Mass *m/z*	Product Mass *m/z*	Murchison	Reagent Blank	Blank	OREX-803004-0	OREX-501029-0
1	4(3H)-Pyrimidinone, 1-tBDMS	31.5 ± 0.8	99.1	45.0	+	+	-	+	+
			153.1	99.0	+	+	-	+	+
			154.1	100.1	+	+	-	+	+
2	Imidazole, 1-tBDMS	32.3 ± 0.8	125.1	98.1	+	-	-	+	+
			155.2	140.1	+	-	-	+	+
			182.2	126.1	-	-	-	+	+
3	1-Methyl-1H-pyrazole-5-carboxylic acid, 1-tBDMS	36.7 ± 0.8	109.1	54.1	n.d.	+	-	n.d.	n.d.
			139.1	59.1	n.d.	+	-	n.d.	n.d.
			183.1	139.1	n.d.	+	-	n.d.	n.d.
4	2-ethyl-4-methylimidazole, 1-tBDMS	38.5 ± 0.8	167.1	109.1	-	-	-	+	+
			168.1	113.1	+	-	-	-	-
			224.2	168.2	+	-	-	+	-
5	Isonicotinic acid, 1-tBDMS	38.8 ± 0.8	106.0	78.1	+	+	-	+	+
			180.1	106.0	+	+	-	+	+
			180.1	136.1	+	+	-	+	+
6	Isocytosine, 1-tBDMS	38.8 ± 0.8	168.1	74.1	n.d.	-	-	+	n.d.
			168.1	99.1	n.d.	-	-	+	n.d.
			168.1	126.1	n.d.	-	-	+	n.d.
7	Alanine, 2-tBDMS	39.6 ± 0.8	158.2	73.1	+	+	-	+	+
			260.2	158.2	+	+	-	+	+
			260.2	232.2	+	+	-	+	+
8	Nicotinic acid+Picolinic acid, 1-tBDMS	39.7 ± 0.8	136.1	94.1	+	-	-	+	+
			180.1	106.0	+	-	-	+	+
			180.1	136.1	+	-	-	+	+
9	2-Methylalanine, 2-tBDMS	40.3 ± 0.8	246.2	147.1	+	+	-	+	+
			274.2	147.1	+	+	-	+	+
			274.2	246.2	+	+	-	+	+
10	Glycine, 2-tBDMS	40.4 ± 0.8	218.2	147.1	+	+	-	+	+
			246.1	147.1	+	+	-	+	+
			246.1	218.2	+	+	-	+	+
11	2-Aminobutanoic acid, 2-tBDMS	40.5 ± 0.8	246.2	147.1	+*	-	-	+	+*
			274.2	147.1	+*	-	-	+	+*
			274.2	246.2	+*	-	-	+	+*
12	β-Alanine, 2-tBDMS	41.5 ± 0.8	218.2	147.1	-	+	-	+	-
			260.2	117.1	-	+	-	-	-
			260.2	218.2	-	+	-	+	-
13	Picolinamide, 1-tBDMS	43.1 ± 0.8	179.1	75.1	+	+	-	+	+
14	Urea, 2-tBDMS	43.4 ± 0.8	147.1	131.1	n.d.	-	-	n.d.	n.d.
			231.1	147.0	n.d.	-	-	n.d.	n.d.
15	Valine, 2-tBDMS	43.5 ± 0.8	186.2	130.1	-	+	-	-	+
			260.2	147.1	-	+	-	-	+
			288.2	260.2	-	+	-	-	+
16	Leucine, 2-tBDMS	44.9 ± 0.8	274.2	147.1	+	+	-	+	+
			302.2	200.2	+	+	-	+	+
			302.2	274.2	+	+	-	+	+
17	Nicotinamide, 1-tBDMS	44.9 ± 0.8	136.1	108.1	-	-	-	-	-
			179.1	105.1	-	-	-	-	-
			179.1	136.1	-	-	-	-	-
18	Isonicotinamide, 1-tBDMS	45.3 ± 0.8	136.1	108.0	-	-	-	-	-
			179.1	136.1	-	-	-	-	-
			180.1	137.1	-	-	-	-	-
19	2,4-Diaminopyrimidine, 1-tBDMS	45.7 ± 0.8	167.1	98.1	-	-	-	-	-
			167.1	125.1	-	-	-	-	-

			167.1	150.1	-	-	-	-	-
20	Isoleucine, 2-tBDMS	45.8 ± 0.8	274.2	147.1	+	+	-	+	+
			302.2	147.1	+	+	-	+	+
			302.2	274.2	+	+	-	+	+
21	Purine, 1-tBDMS	46.0 ± 0.8	177.1	123.1	-	-	-	-	-
			178.1	136.0	-	-	-	-	-
			178.1	163.1	-	-	-	-	-
22	Proline, 2-tBDMS	46.9 ± 0.8	184.2	73.1	-	-	-	+	+
			258.2	147.1	-	-	-	n.d.	+
23	Uracil, 2-tBDMS	46.9 ± 0.8	283.1	73.0	-	+	-	+	+
			283.1	99.1	-	+	-	+	+
			283.1	147.1	-	+	-	+	+
24	6-Methyluracil, 3-tBDMS	47.9 ± 0.8	297.2	147.1	+	+	-	+	+
			298.2	148.1	+	+	-	+	+
			298.2	241.1	-	-	-	+	n.d.
25	Thymine, 2-tBDMS	49.2 ± 0.8	297.1	113.0	-	-	-	+	+
			297.1	147.1	-	-	-	+	+
			297.1	255.2	-	-	-	+	+
26	1-Methyluracil, 1-tBDMS	49.2 ± 0.8	100.0	72.0	-	-	-	n.d.	-
			183.1	72.0	-	-	-	n.d.	-
			183.1	100.0	-	-	-	n.d.	-
27	Isocytosine, 2-tBDMS	49.8 ± 0.8	282.1	125.1	-	-	-	+	+
			282.1	171.1	-	-	-	+	+
			283.2	172.1	-	-	-	+	+
28	2-imidazole-carboxylic acid, 2-tBDMS	50.3 ± 0.8	283.2	73.1	-	+	-	+	+
			283.2	239.2	-	-	-	+	+
29	Cytosine, 2-tBDMS	52.3 ± 0.8	282.1	170.1	-	-	-	-	+
			282.1	212.2	-	-	-	-	+
			283.2	213.2	-	-	-	-	+
30	Pyroglutamic acid, 2-tBDMS	52.4 ± 0.8	147.1	131.1	-	-	-	+	+
			272.2	147.1	-	-	-	+	+
			300.1	272.2	-	-	-	+	+
31	Methionine, 2-tBDMS	52.8 ± 0.8	218.1	170.2	-	-	-	-	+
			292.2	147.1	-	-	-	-	+
			320.2	292.2	-	-	-	-	+
32	5-Methylcytosine, 2-tBDMS	53.1 ± 0.8	296.2	112.1	-	-	-	-	+
			296.2	182.1	-	-	-	-	+
			296.2	226.2	-	-	-	-	+
33	Serine, 3-tBDMS	53.4 ± 0.8	362.2	147.1	-	-	-	+	+
			390.2	230.2	-	-	-	+	+
			390.2	362.2	-	-	-	+	+
34	Threonine, 3-tBDMS	54.3 ± 0.8	303.2	148.1	-	-	-	-	+
			303.2	202.1	-	-	-	-	+
			303.2	287.2	-	-	-	-	+
35	2,4-Diaminopyrimidine, 2-tBDMS	54.4 ± 0.8	281.2	125.1	-	-	-	-	-
			281.2	170.1	-	-	-	+	+
			281.2	212.2	+	-	-	+	+
36	Phenylalanine, 2-tBDMS	56.1 ± 0.8	234.2	178.1	-	-	-	-	+
			308.2	147.1	-	-	-	-	+
			336.2	308.2	-	-	-	-	+
37	Aspartic acid, 3-tBDMS	57.8 ± 0.8	302.2	147.1	-	-	-	-	+
			390.2	147.1	-	-	-	-	+
			390.2	346.3	-	-	-	-	+
38	4-imidazole-carboxylic acid, 2-tBDMS	58.3 ± 0.8	169.1	75.1	-	-	-	+	+
			169.1	125.1	-	-	-	+	+
			283.2	73.1	-	+	-	+	+
39	Hypoxanthine, 2-tBDMS	69.7 ± 0.8	193.1	111.0	-	-	-	-	-
			307.2	193.1	-	-	-	-	-
			307.2	251.1	-	-	-	-	-
40	Glutamic acid, 3-tBDMS	60.7 ± 0.8	272.2	147.1	-	-	-	+	+
			330.2	170.1	-	-	-	-	+
			432.3	272.2	-	-	-	+	+
41	Asparagine, 2-tBDMS	61.5 ± 0.8	302.2	147.1	-	-	-	+	-
			417.2	147.1	-	-	-	+	+
			417.2	400.2	-	-	-	-	-
42	Adenine, 2-tBDMS	61.6 ± 0.8	192.1	165.1	-	-	-	-	-
			306.2	192.1	-	-	-	-	-
			307.2	193.1	-	-	-	-	-

43	Lysine, 3-tBDMS	63.2 ± 0.8	300.2	147.1	-	-	-	-	-
			300.2	168.1	-	-	-	-	-
			300.2	272.2	-	-	-	-	-
44	2,6-Diaminopurine, 2-tBDMS	66.8 ± 0.8	321.2	73.1	-	-	-	-	-
			321.2	263.1	-	-	-	-	-
			321.2	305.2	-	-	-	-	-
45	Histidine, 2-tBDMS	67.9 ± 0.8	338.3	197.2	-	-	-	-	-
			440.3	280.1	-	-	-	-	-
			440.3	412.2	-	-	-	-	-
46	Xanthine, 3-tBDMS	68.3 ± 0.8	437.2	147.1	-	-	-	-	-
			437.2	363.2	-	-	-	-	-
			437.2	436.1	-	-	-	-	-
47	Tyrosine, 3-tBDMS	69.0 ± 0.8	302.2	147.1	-	-	-	-	-
			302.2	218.2	-	-	-	-	-
			302.2	245.1	-	-	-	-	-
48	Tryptophan, 2-tBDMS	69.9 ± 0.8	302.2	73.1	-	-	-	-	-
			302.2	147.1	-	-	-	-	-
			302.2	218.2	-	-	-	-	-
49	Guanine, 3-tBDMS	70.4 ± 0.8	436.3	264.1	-	-	-	-	-
			436.3	322.1	-	-	-	-	-
			436.3	435.4	-	-	-	-	-
50	2,6-Diaminopurine, 3-tBDMS	72.2 ± 0.8	435.3	263.1	+	-	-	-	-
			435.3	377.1	+	-	-	-	+
			435.3	419.2	+	-	-	-	-
51	Tryptophan, 3-tBDMS	74.3 ± 0.8	244.2	73.1	-	-	-	-	-
			244.2	188.1	-	-	-	-	-
			245.2	189.2	-	-	-	-	-
52	Cystine, 4-tBDMS	79.4 ± 0.8	348.2	106	-	-	-	-	-
			348.2	188.1	-	-	-	-	-
			348.2	302.2	-	-	-	-	-

n.d. = not determined due to MRM crosstalk from analogous ions.
*scan window insufficient to capture the entire peak.

Supplementary Table 12. A comparison of the standard protein amino acids detected in the Bennu aggregate samples compared to those reported in the meteorite literature. Amino acids with a green check mark indicate they were detected. The red X indicates the amino acid was not detected in the current study or has not been published in the literature[13].

Standard Protein Amino Acids	Meteorite Literature	OREX-803004-0 (TAGSAM) MTBSTFA PyGC-MS	OREX-501029-0 (Avionics Deck) MTBSTFA PyGC-MS	OREX-803001-0 (TAGSAM) OPA/NAC LC-MS	OREX-803001-0 (TAGSAM) AccQ-TAG LC-MS
glycine	✓	✓	✓	✓	✓
alanine	✓	✓	✓	✓	✓
proline	✓	✓	✓	n/a	✓
valine	✓	X	✓	✓	✓
leucine	✓	X	✓	✓	✓
isoleucine	✓	X	✓	✓	✓
methionine	✓[a]	X	tentative	n.d.	X
phenylalanine	✓	X	✓	n.d.	✓
threonine	✓	✓	✓	✓	✓
serine	✓	✓	✓	✓	✓
aspartic acid	✓	X	✓	✓	✓
glutamic acid	✓	✓	✓	✓	✓
asparagine	✓[b]	✓	✓	n.d.[c]	tentative[c]
glutamine	X	X	X	n.d.[c]	X[c]
tyrosine	✓	X	X	n.d.	tentative
tryptophan	X	X	X	n.d.	X
lysine	X	X	X	n.d.	X
arginine	X	X	X	n.d.	X
histidine	X	X	X	n.d.	X
cysteine	X	X	X	n.d.[c]	X[c]

[a]Single report of a weak identification in the CM2 carbonaceous chondrites ALHA 77306 and Murchison meteorite[102].
[b]First reported detection in the CM2 Murchison meteorite[54].
[c]Unstable in hot water and may have decomposed during extraction.
n.d. = not determined.
n/a = not detectable with the OPA/NAC derivatization method that does not label secondary amines.
Tentative = peak observed above background levels, but near limit of detection.

Supplementary Table 13. Qualitative comparison of the N-heterocycle detections in Bennu aggregate samples. Detections are indicated by a green check mark, and a red x indicates the compound was not detected. Previously published data from Ryugu (A0106 and C0107) and the CM2 Murchison meteorite are also shown for comparison.

Compound	OREX-501029-0	OREX-803004-0	OREX-800044-101	Ryugu[a]	Murchison[b]
Uracil	✓	✓	✓	✓	✓
Thymine	✓	✓	✓	X	✓
Cytosine	✓	X	✓	X	✓
1-Methyluracil	X	tentative	✓	X	✓
6-Methyluracil	✓	✓	✓	X	✓
5-Methylcytosine	✓	X	X	X	X
Isocytosine	✓	✓	X	X	X
2,4-Diaminopyrimidine	tentative	tentative	X	X	X
Adenine	X	X	✓	X	✓
Guanine	X	X	✓	X	✓
Purine	X	X	✓	X	✓
Hypoxanthine	X	X	✓	X	✓
Xanthine	X	X	✓	X	✓
Isoguanine	n.d.	n.d.	✓	X	✓
Diaminopurine (2,6- or 6,8-)	tentative	X	X	X	✓
Nicotinic acid	tentative	tentative	✓	✓	✓
Isonicotinic acid	✓	✓	✓	✓	✓
2-Methylnicotinic acid	n.d.	n.d.	✓	X	X
5-Methylnicotinic acid	n.d.	n.d.	✓	X	X
6-Methylnicotinic acid	n.d.	n.d.	✓	X	X
Picolinamide	tentative	tentative	tentative	X	tentative
Imidazole	✓	✓	tentative	n.d.	tentative
2-Imadazole carboxylic acid	✓	✓	✓	✓	✓
4-Imadazole carboxylic acid	✓	✓	✓	✓	✓
2-Methyl-1H-imidazole carboxylic acid	n.d.	n.d.	✓	n.d.	n.d.

[a]Data from ref. [33].
[b]Data from refs. [33,34,63].
n.d. = not determined.
Tentative = peak observed above background levels, but near limit of detection.

Supplementary Table 14. List of the measurement data products from the Bennu samples analyzed in this study and corresponding DOIs available at https://astromat.org.

EA-IRMS Data

DOI	Product Name	Product Type
**EA-IRMS**	OREX-501033-0, OREX-501034-0, OREX-501035-0, OREX-501036-0, OREX-501037-0, OREX-501038-0, OREX-501039-0, OREX-501040-0, OREX-501041-0, OREX-803002-0, OREX-803040-0, OREX-803041-0, OREX-803042-0, OREX-803043-0, OREX-803044-0, OREX-803045-0, OREX-803046-0, OREX-803001-104, OREX-803001-105, OREX-803001-106, OREX-803007-108, OREX-803001-109, OREX-803001-110	
**10.60707/g1fx-9s05**	20231210_EAIRMS_CIS_multiSample_2_EAIRMSCollection_1.zip	EAIRMSCollection
**10.60707/ndf3-qn80**	20231210_EAIRMS_CIS_multiSample_1_EAIRMSCollection_1.zip	EAIRMSCollection
**10.60707/0g1m-4v39**	20231209_EAIRMS_CIS_multiSample_1_EAIRMSCollection_1.zip	EAIRMSCollection
**10.60707/m5mw-kj32**	20231208_EAIRMS_CIS_multiSample_1_EAIRMSCollection_1.zip	EAIRMSCollection
**10.60707/6c5n-e486**	20231005_EAIRMS_CIS_multiSample_1_EAIRMSCollection_1.zip	EAIRMSCollection
**10.60707/wg35-6e70**	20231005_EAIRMS_CIS_OREX-501033-0_1_EAIRMSCollection_1.zip	EAIRMSCollection
**10.60707/t5ac-es57**	20231004_EAIRMS_CIS_multiSample_2_EAIRMSCollection_1.zip	EAIRMSCollection
**Nano EA-IRMS**	OREX-803001-112	
**10.60707/7h3b-xk29**	20240423_EA-IRMS_PSU_OREX-803001_112_1_EAIRMSCollection_1.zip	EAIRMSCollection

VIS-UV imaging/μL^{2}MS Data

DOI	Product Name	Product Type
**VIS-UV imaging**	OREX-501006-0	
**10.60707/wspc-wg10**	20231002_UVFM_JSC-ARES_OREX-501006-0_1_UVFMImage_1.tif	UVFMImage
**μL^{2}MS**	OREX-501006-0	
**10.60707/srdv-7b95**	20231127_uL2MS_JSC-ARES_OREX-501006-0_1_L2MSCube_11.h5	L2MSCube

GCMS Data

DOI	Product Name	Product Type
**PyGCMS**	OREX-501028-0, OREX-501029-0, OREX-803003-0, OREX-803004-0	
**10.60707/579m-1256**	20231003_GC-MS_GSFC_OREX-501028-0_1_GCMSCollection_1.zip	GCMSCollection
**10.60707/me36-7c97**	20231003_GC-MS_GSFC_OREX-501029-0_1_GCMSCollection_1.zip	GCMSCollection
**10.60707/yv1f-jb20**	20231121_GC-MS_GSFC_OREX-803003-0_1_GCMSCollection_1.zip	GCMSCollection
**10.60707/9ww1-7a05**	20231109_GC-MS_GSFC_OREX-803004-0_1_GCMSCollection_1.zip	GCMSCollection
**GCMS**	OREX-803001-0	
**10.60707/5me5-cm54**	20231201_GC-MS_GSFC_OREX-803001-0_1_GCMSCollection_315.zip	GCMSCollection

LCMS Data

DOI	Product Name	Product Type
**LCMS**	OREX-803001-0	
**10.60707/3gcb-z762**	20240131_LC-MS_GSFC_OREX-803001-0_1_LCMSCollection_1.zip	LCMSCollection
**10.60707/cw79-c829**	20231219_LC-MS_GSFC_OREX-803001-0_1_LCMSCollection_1.zip	LCMSCollection
**10.60707/dt7a-vp76**	20240201_LC-MS_GSFC_OREX-803001-0_1_LCMSCollection_1.zip	LCMSCollection
**10.60707/81ff-sx95**	20231114_LC-MS_GSFC_OREX-803001-0_1_LCMSCollection_1.zip	LCMSCollection
**10.60707/q6zw-mb66**	20231115_LC-MS_GSFC_OREX-803001-0_1_LCMSCollection_1.zip	LCMSCollection
**10.60707/pwm6-na19**	20231116_LC-MS_GSFC_OREX-803001-0_1_LCMSCollection_1.zip	LCMSCollection
**10.60707/bt5a-4e54**	20231117_LC-MS_GSFC_OREX-803001-0_1_LCMSCollection_1.zip	LCMSCollection
**10.60707/c0v3-d379**	20240123_LC-MS_GSFC_OREX-803001-0_1_LCMSCollection_1.zip	LCMSCollection
**10.60707/gyq2-mq38**	20240124_LC-MS_GSFC_OREX-803001-0_1_LCMSCollection_1.zip	LCMSCollection
**LCMS**	OREX-800044-101	
**10.60707/m53m-r760**	20240301_LC-MS_KU_OREX-800044-101_1_LCMSCollection_10.zip	LCMSCollection
**10.60707/xafm-3b57**	20240301_LC-MS_KU_OREX-800044-101_1_LCMSCollection_1.zip	LCMSCollection
**10.60707/m48s-xe07**	20240301_LC-MS_KU_OREX-800044-101_1_LCMSCollection_11.zip	LCMSCollection
**10.60707/92y7-at49**	20240301_LC-MS_KU_OREX-800044-101_1_LCMSCollection_12.zip	LCMSCollection
**10.60707/nz85-cr04**	20240301_LC-MS_KU_OREX-800044-101_1_LCMSCollection_13.zip	LCMSCollection
**10.60707/s4e0-tk28**	20240301_LC-MS_KU_OREX-800044-101_1_LCMSCollection_14.zip	LCMSCollection
**10.60707/1h12-7408**	20240301_LC-MS_KU_OREX-800044-101_1_LCMSCollection_15.zip	LCMSCollection

**10.60707/3mpq-xb27**	20240301_LC-MS_KU_OREX-800044-101_1_LCMSCollection_2.zip	LCMSCollection
**10.60707/vswb-pw60**	20240301_LC-MS_KU_OREX-800044-101_1_LCMSCollection_4.zip	LCMSCollection
**10.60707/3brh-2b78**	20240301_LC-MS_KU_OREX-800044-101_1_LCMSCollection_5.zip	LCMSCollection
**10.60707/b9mm-0r98**	20240301_LC-MS_KU_OREX-800044-101_1_LCMSCollection_6.zip	LCMSCollection
**10.60707/jyjn-yh28**	20240301_LC-MS_KU_OREX-800044-101_1_LCMSCollection_7.zip	LCMSCollection
**10.60707/2x5w-0w88**	20240301_LC-MS_KU_OREX-800044-101_1_LCMSCollection_8.zip	LCMSCollection
**10.60707/1f3g-x731**	20240301_LC-MS_KU_OREX-800044-101_1_LCMSCollection_9.zip	LCMSCollection

FTICR-MS Data

DOI	Product Name	Product Type
**FTICR-MS**	OREX-803006-0	
**10.60707/tc63-1847**	20240530_FTICR-MS_HMGU_OREX-803006-0_1_FTICRMSCube_1.mzml	FTICRMSCube
**10.60707/ff9b-4j27**	20240530_FTICR-MS_HMGU_OREX-803006-0_1_FTICRMSCube_2.mzml	FTICRMSCube
**10.60707/4ceq-2x52**	20240530_FTICR-MS_HMGU_OREX-803006-0_1_FTICRMSCube_3.mzml	FTICRMSCube
**10.60707/gk8m-0h97**	20240530_FTICR-MS_HMGU_OREX-803006-0_1_FTICRMSCube_4.mzml	FTICRMSCube
**10.60707/1knn-kw48**	20240530_FTICR-MS_HMGU_OREX-803006-0_1_FTICRMSCube_5.mzml	FTICRMSCube
**10.60707/f6fk-dt75**	20240530_FTICR-MS_HMGU_OREX-803006-0_1_FTICRMSCube_6.mzml	FTICRMSCube
**10.60707/66gm-xn41**	20240530_FTICR-MS_HMGU_OREX-803006-0_1_FTICRMSCube_7.mzml	FTICRMSCube
**10.60707/q8jg-v633**	20240530_FTICR-MS_HMGU_OREX-803006-0_1_FTICRMSCube_8.mzml	FTICRMSCube
**10.60707/r8hy-7y50**	20240530_FTICR-MS_HMGU_OREX-803006-0_1_FTICRMSTabular_1.csv	FTICRMSTabular
**10.60707/ampq-z880**	20240530_FTICR-MS_HMGU_OREX-803006-0_1_FTICRMSTabular_2.csv	FTICRMSTabular
**10.60707/7aye-1h38**	20240530_FTICR-MS_HMGU_OREX-803006-0_1_FTICRMSTabular_5.csv	FTICRMSTabular
**10.60707/4jp0-cb92**	20240530_FTICR-MS_HMGU_OREX-803006-0_1_FTICRMSTabular_4.csv	FTICRMSTabular
**10.60707/48ne-8t35**	20240530_FTICR-MS_HMGU_OREX-803006-0_1_FTICRMSTabular_3.csv	FTICRMSTabular
**10.60707/5pt4-kr47**	20240530_FTICR-MS_HMGU_OREX-803006-0_1_FTICRMSTabular_6.csv	FTICRMSTabular
**10.60707/jk2s-6k42**	20240530_FTICR-MS_HMGU_OREX-803006-0_1_FTICRMSTabular_7.csv	FTICRMSTabular
**10.60707/3290-8d50**	20240530_FTICR-MS_HMGU_OREX-803006-0_1_FTICRMSTabular_8.csv	FTICRMSTabular
